# A deep joint-learning proteomics model for diagnosis of six conditions associated with dementia

**DOI:** 10.1038/s41591-026-04303-y

**Published:** 2026-03-31

**Authors:** Lijun An, Alexa Pichet Binette, Ines Hristovska, Gabriele Vilkaite, Yu Xiao, Romina Zendehdel, Zijian Dong, Bart Smets, Rowan Saloner, Shinya Tasaki, Ying Xu, Varsha Krish, Farhad Imam, Shorena Janelidze, Danielle van Westen, The Global Neurodegenerative Proteomics Consortium (GNPC), Lijun An, Alexa Pichet Binette, Bart Smets, Rowan Saloner, Shinya Tasaki, Ying Xu, Varsha Krish, Farhad Imam, Niklas Mattsson-Carlgren, Oskar Hansson, Jacob W. Vogel, Erik Stomrud, Christopher D. Whelan, Sebastian Palmqvist, Rik Ossenkoppele, Niklas Mattsson-Carlgren, Oskar Hansson, Jacob W. Vogel

**Affiliations:** 1 Department of Clinical Sciences Malmö, SciLifeLab, Lund University, Lund, Sweden; 2 Clinical Memory Research Unit, Department of Clinical Sciences Malmö, Lund University, Lund, Sweden; 3 Department of Physiology and Pharmacology, Université de Montréal, Montreal, Quebec Canada; 4 Centre de Recherche de l’institut Universitaire de Gériatrie de Montréal, Montreal, Quebec Canada; 5 Centre for Sleep and Cognition and Centre for Translational MR Research, Yong Loo Lin School of Medicine, National University of Singapore, Singapore, Singapore; 6 Department of Electrical and Computer Engineering, National University of Singapore, Singapore, Singapore; 7 Johnson & Johnson, Beerse, Belgium; 8 Memory and Aging Center, Department of Neurology, Weill Institute for Neurosciences, University of California, San Francisco, CA USA; 9 Rush Alzheimer’s Disease Center, Rush University Medical Center, Chicago, IL USA; 10 Department of Psychiatry, Washington University School of Medicine, St. Louis, MO USA; 11 NeuroGenomics and Informatics Center, Washington University School of Medicine, St. Louis, MO USA; 12 Gates Ventures, Seattle, WA USA; 13 Department of Diagnostic Radiology, Clinical Sciences, Lund University, Lund, Sweden; 14 Image and Function, Skåne University Hospital, Lund, Sweden; 15 Memory Clinic, Skåne University Hospital, Malmo, Sweden; 16 RCSI University of Medicine and Health Sciences, Royal College of Surgeons in Ireland, Dublin, Ireland; 17 Johnson & Johnson, Cambridge, MA USA; 18 Amsterdam Neuroscience, Neurodegeneration, Vrije Universiteit Amsterdam, Amsterdam, the Netherlands; 19 Alzheimer Center Amsterdam, Neurology, Vrije Universiteit Amsterdam, Amsterdam UMC Location VUmc, Amsterdam, the Netherlands

**Keywords:** Neurodegenerative diseases, Predictive markers, Neurological disorders

## Abstract

Co-pathology is a common feature of neurodegenerative diseases that complicates diagnosis, treatment and clinical management. However, sensitive, specific and scalable biomarkers for in vivo pathological diagnosis are not available for most neurodegenerative neuropathologies. Here we present Proteomics-based Artificial Intelligence for Dementia Diagnosis (ProtAIDe-Dx), a deep joint-learning model on 17,187 patients and controls (age of 70.3 ± 11.5 years, 53.2% female), that uses plasma proteomics to provide simultaneous probabilistic diagnosis across 6 conditions associated with dementia in aging. ProtAIDe-Dx achieves cross-validated balanced classification accuracy of 70–95% and area under the curve of >78% across all conditions. The model’s diagnostic probabilities highlighted subgroups of patients with co-pathologies and were associated with pathology-specific biomarkers in an external memory clinic sample, even among individuals without cognitive impairment. Model interpretation revealed a suite of protein networks marking shared and specific biological processes across diseases and identified novel and previously described proteins discriminating each diagnosis. ProtAIDe-Dx significantly improved biomarker-based differential diagnosis in a memory clinic sample, pinpointing proteins leading to diagnostic decisions at an individual level. Together, this work highlights the promise of plasma proteomics to improve patient-level diagnostic workup with a single blood draw.

## Abstract (web summary)

ProtAIDe-Dx is a deep joint-learning model that uses plasma proteomics to provide simultaneous probabilistic diagnoses across six conditions associated with aging.

## Main

The past 5 years have seen multiple breakthroughs in the treatment of neurodegenerative diseases. Early disease-modifying therapies have emerged for Alzheimer’s disease (AD)^1,2^, and highly promising drug candidates are currently in clinical trials for AD^3^, Parkinson’s disease (PD)^4^ and amyotrophic lateral sclerosis (ALS)^5^. However, differential diagnosis and disease comorbidity continue to pose considerable challenges in these treatment efforts. Misdiagnosis rates are around 25–30% even in specialized dementia clinics and can exceed 50% in primary care^6–8^. Meanwhile, comorbidity is common in aging, with 70% of patients 80 years or older harboring multiple neurodegenerative pathologies simultaneously^9^. Misdiagnosis can make it difficult to select the right patients for a drug trial^10^, while comorbid neuropathologies can mask the positive effects of a putative therapy^11,12^. Once such treatments do become available, misdiagnosis and comorbidity can both lead to treatment mismanagement^13^. With the rapid pace at which promising new drugs are being tested, there is an urgent need for powerful tools for diagnosis and precise identification of underlying comorbid pathologies.

The first step toward mitigating the issues presented by misdiagnosis and comorbidity is the development of biomarkers to identify underlying neurodegenerative pathology with high specificity. Blood-based biomarkers have the potential to be highly accessible, inexpensive and minimally invasive, and the emergence of blood-based biomarkers for AD could facilitate accurate AD diagnosis even in primary care in the near future^7^. Despite the success of AD biomarkers, scalable, sensitive and specific biomarkers for other neurodegenerative diseases are lacking, and diagnosis can only be made with high confidence at autopsy. Plasma proteomics represent a promising tool toward this aim, allowing robust surveillance of thousands of potential biomarkers and relevant functional effectors with a single blood draw^14^. However, despite great promise, plasma proteomics data are not without challenges. Proteomics data are high rank, come with burdensome technological artifacts and probably represent complex nonlinear interactions^15,16^. In addition, the blood–brain barrier limits the number of brain-expressed proteins relevant in neurological conditions that can be detected in blood^17^.

**Fig. 1:**
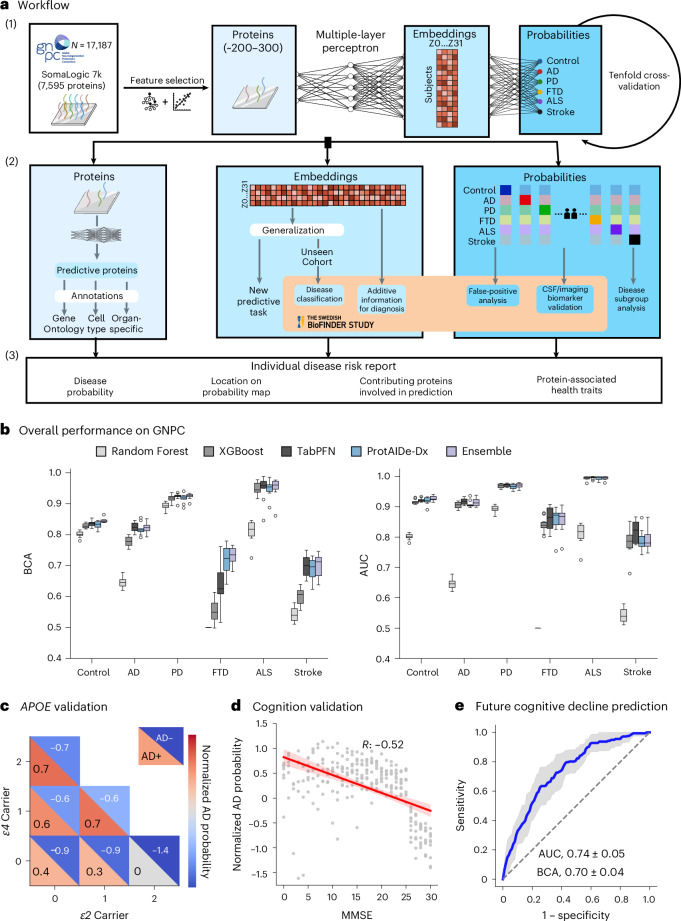
Workflow and overall performance of ProtAIDe-Dx on GNPC. **a**, Project workflow. (1) Model development on GNPC with tenfold cross-validation procedure. The joint learning framework allows learning of multiple neurodegenerative classification tasks jointly, facilitating shared information during training. (2) Model evaluation on GNPC (blue panels) and BioFINDER-2 cohort (orange panel). Model outputs include not just classifications but also probabilities for each class, and contributing proteins and embeddings can be probed to better understand model choices. (3) Individual neurological disease risk report based on the developed model. **b**, Overall model performance on GNPC. Left: BCA score for each diagnostic task. Right: AUC score for each diagnostic task. Box plots were drawn across 10 cross-validation folds, showing the median, interquartile range (IQR; 25th to 75th percentiles) and whiskers extending to 1.5× IQR. Two-sided corrected resample *t*-tests were applied; *P* values were FDR corrected. **c**, Normalized AD probabilities stratified by different *APOE* *ε2*/*ε4* groups, shown for participants with (lower diagonal) and without (upper diagonal) an AD diagnosis. **d**, Correlation between normalized AD probabilities and MMSE. Error bands represent the 95% confidence interval of the regression line. **e**, Receiver operating characteristic (ROC) curve of model generalization to a new task for predicting longitudinal clinical progression (from no cognitive impairment to future cognitive impairment). Shaded regions represent the mean ROC ± 1 s.d. across ten cross-validation folds. Schematic and logo in **a** created in BioRender; An, L. https://biorender.com/q2by4y5 (2026).

In the present study, we attempt to overcome these limitations by applying artificial intelligence (AI) to the Global Neurodegenerative Proteomics Consortium (GNPC)^18^ v1.3MS dataset, the largest neurodegenerative disease plasma proteomics dataset so far. We present a model called Proteomics-based AI for Dementia Diagnosis (ProtAIDe-Dx), a deep multi-task architecture to resolve differential and multiple neurodegenerative diagnoses with a single blood draw (Fig. 1a). We report the performance of ProtAIDe-Dx on multi-diagnosis prediction, evaluate its potential in identifying co-pathology, test its capability in differential diagnosis compared with other clinical markers and explore putative molecular networks contributing to its predictions. Finally, we present a proof of concept using ProtAIDe-Dx for personalized and interpretable diagnostic testing in a clinical scenario. With this approach, we hope to set a benchmark for future plasma-based multi-disease neurodegenerative diagnostic tools.

## Results

A subsample of 17,187 participants with SomaLogic 7k proteomics, sampled across 19 contributing sites, was selected from the GNPC v1.3MS dataset for subsequent analysis (Methods). Supplementary Table 1 presents sample and demographics across contributing sites and Supplementary Table 2 presents the frequency of six conditions across each site, namely, AD, PD, frontotemporal dementia (FTD), ALS, previous stroke/transient ischemic attack (TIA) and cognitive unimpairment. Given the high prevalence of vascular dementia (second only to AD^19^) but the lack of vascular dementia diagnoses in GNPC, the stroke/TIA group was chosen as a representation of patients with documented cerebrovascular disease.

We applied the ProtAIDe-Dx model to this sample, an architecture capable of generating several features of interest simultaneously: binary diagnosis of each condition, probabilities of each diagnosis and joint embeddings representing low-dimensional, nonlinear protein combinations used by the model for diagnosis (Fig. 1a). We specifically chose a multi-task, joint-learning approach (as opposed to a multi-class classification task) to allow the model to signal disease co-pathology (that is, positive for multiple disorders and probabilities for each disorder).

### Joint learning improves multi-diagnostic prediction of neurodegenerative diagnosis from blood in unbalanced samples

We applied ProtAIDe-Dx to the GNPC sample, using tenfold cross-validation stratified for each contributing site. Importantly, we used only proteomic information in the model—no site, demographic, cognitive or diagnostic information was used. We compared the diagnostic performance of ProtAIDe-Dx against multiple machine learning and state-of-the-art deep learning baselines, including Random Forest, XGBoost^20^ and TabPFN^21^. We also tested an ensemble model combining aspects of both XGBoost and ProtAIDe-Dx (Methods).

ProtAIDe-Dx emerged as the best-performing model overall, while XGBoost was the best non-deep learning model (Fig. 1b). ProtAIDe-Dx achieved a median balanced classification accuracy (BCA) performance above 90% for ALS (95%) and PD (92%) classification, 83% for control, 81% for AD, 72% for FTD and 70% for stroke/TIA (Fig. 1b). ProtAIDe-Dx significantly outperformed Random Forest across all tasks; XGBoost in AD (false discovery rate (FDR)-corrected *P* value of 5 × 10^−4^), FTD (FDR-corrected *P* value of 3 × 10^−^^4^) and stroke (FDR-corrected *P* of 0.004) classification; and significantly outperformed TabPFN in FTD classification (FDR-corrected *P* value of 0.047). With the exception of Random Forest, all models achieved area under the curve (AUC) >0.8 for all tasks other than stroke/TIA prediction and demonstrated comparable AUCs (Fig. 1b, Supplementary Table 3 and Supplementary Data 1), although AUC alone might convey overoptimistic implications in some imbalanced classification scenarios^22^ (Supplementary Fig. 1). We found that the ensemble model produced balanced accuracy scores and AUCs that significantly outperformed ProtAIDe-Dx for control and PD diagnosis. The ensemble model also significantly improved BCA scores over XGBoost for all tasks except ALS and PD prediction (Fig. 1b, Supplementary Table 3 and Supplementary Data 1).

As a sanity check, we extracted the probability of AD diagnosis across all individuals and compared them with factors known to be altered in AD. In patients both with and without an AD diagnosis, higher AD probabilities were associated with more copies of the *APOE* *ε4* allele and lower AD probabilities with more copies of the *ε2* allele (Fig. 1c). In addition, a negative correlation was observed between AD probabilities and Mini-Mental State Examination (MMSE) score, indicating worse cognition associated with higher AD probabilities (Fig. 1d). These analyses suggest that model-derived diagnostic probabilities can serve as continuous proteomic scores associated with indicators of disease progression.

### Diagnostic prediction model generalizes to new disease-relevant tasks

Low-dimensional nonlinear proteomic embeddings were extracted from the last layer of the ProtAIDe-Dx model (Fig. 1a). These embeddings should represent a compressed representation of plasma proteomic data, optimized toward tasks related to neurological diseases. To test this hypothesis, we used the embeddings to generalize the ProtAIDe-Dx model to a task that ProtAIDe-Dx was not trained specifically for, namely, prediction of longitudinal clinical progression in healthy controls (Methods). The model differentiated diagnostic progressors (that is, from clinical dementia rating (CDR) 0 to CDR 0.5 or 1; *N* = 218) from non-progressors (remained stably at CDR 0 over time; *N* = 1,445) with a BCA of 70% and an AUC of 74% (Fig. 1e). These results support ProtAIDe-Dx as a flexible and extensible model for neurodegenerative disease-related tasks.

### Diagnostic probabilities reveal disease heterogeneity and co-pathology

**Fig. 2:**
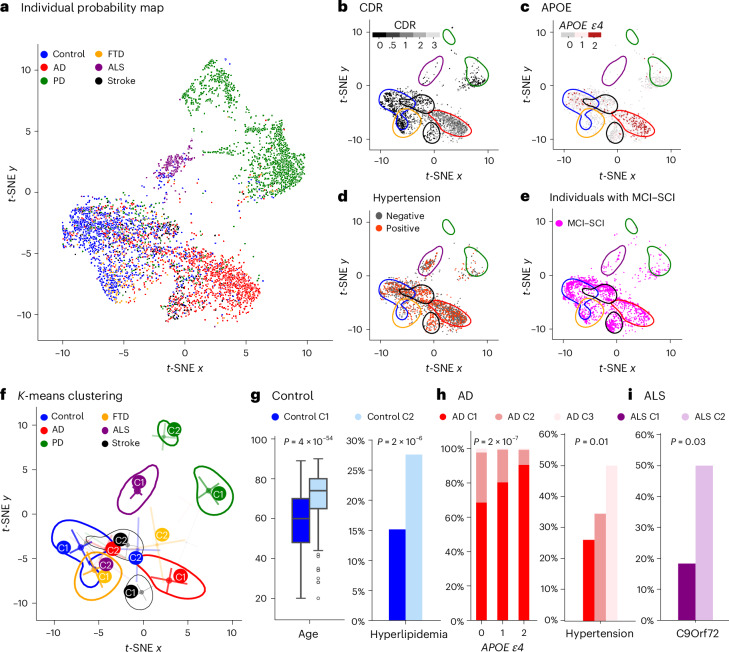
Diagnostic probability map derived by ProtAIDe-Dx reveals disease heterogeneity. **a**, Participants from the test set were selected for *t*-SNE to reduce dimensionality from six (predicted class probabilities) to two (nonlinear disease probability axes), colored by clinical diagnosis. **b**–**e**, Participants were projected onto the two-dimensional diagnostic probability map, colored by values of CDR (**b**), APOE (**c**), hypertension (**d**) and individuals diagnosed with MCI-SCI (**e**). **f**, *K*-means clustering was performed within each diagnostic category on the basis of their disease probabilities, where the top two clusters were annotated. The contour was drawn on the basis of Gaussian kernel density estimation with a threshold of 0.01. **g**–**i**, Distributions of variables vary across the control (**g**), AD (**h**) and ALS (**i**) clusters (see Extended Data Fig. 2 for further details on disease clusters). Age distributions in **g** are shown for control C1 (*n* = 1,132) and control C2 (*n* = 407) as box plots indicating the median (center), interquartile range (25th to 75th percentiles) and whiskers extending to 1.5× IQR. *P* values were FDR corrected.

**Extended Data Fig. 1:**
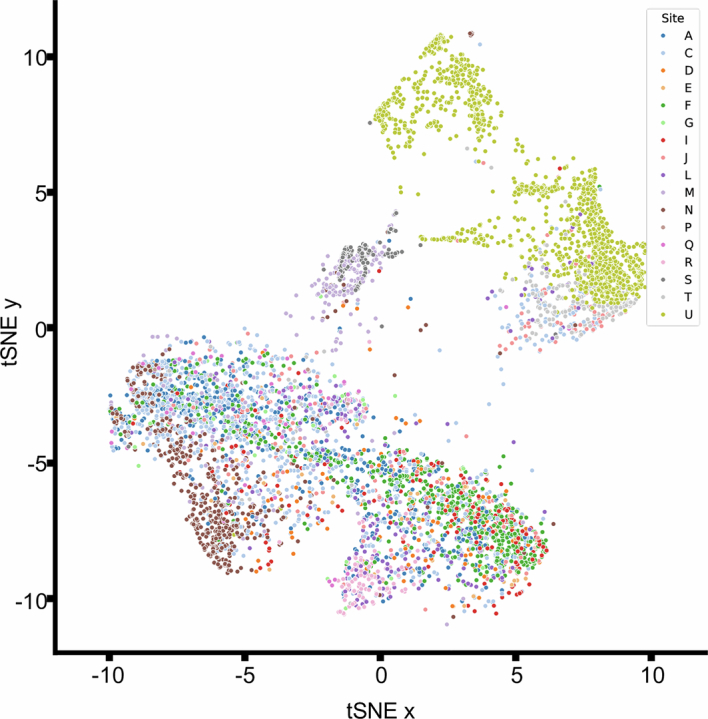
Site distribution of 6,332 test participants on the t-SNE diagnostic probability map. The test participants and t-SNE embedding were the same as in Fig. 2, while the participants were re-colored by sites.

ProtAIDe-Dx provides probabilities of each condition for each individual. We projected all individuals into a two-dimensional nonlinear embedding on the basis of their disease probabilities (Fig. 2a). As expected, individuals naturally clustered on the basis of their true clinical diagnosis and not by contributing site (Extended Data Fig. 1). Common phenotypic data in the GNPC distributed in expected patterns across the embeddings, with worse cognitive impairment and more *APOE* *ε4* carriers in AD regions, fewer *ε4* carriers in PD and ALS regions and more hypertension in the stroke/TIA region (Fig. 2b–d).

Next, we used ProtAIDe-Dx to predict etiological diagnoses of patients with ambiguous etiologies that were not used in model training, namely, patients diagnosed with subjective cognitive decline (SCD) or mild cognitive impairment (MCI; *N* = 3,116) and patients characterized as ‘HealthyAD’ (diagnosis of AD but cognitive scores in the healthy range), ‘ComputedDementia’ (no diagnosis but cognitive scores in the dementia range) and ‘Unknown’ (no diagnostic or cognitive information) groups (Methods). When projecting these cases onto the diagnostic embedding (Fig. 2e and Supplementary Fig. 2), the cases were distributed throughout the embedding, with cases falling neatly into regions corresponding to different conditions. This signals the potential for ProtAIDe-Dx to aid in the diagnosis of patients in early phases of impairment.

**Extended Data Fig. 2:**
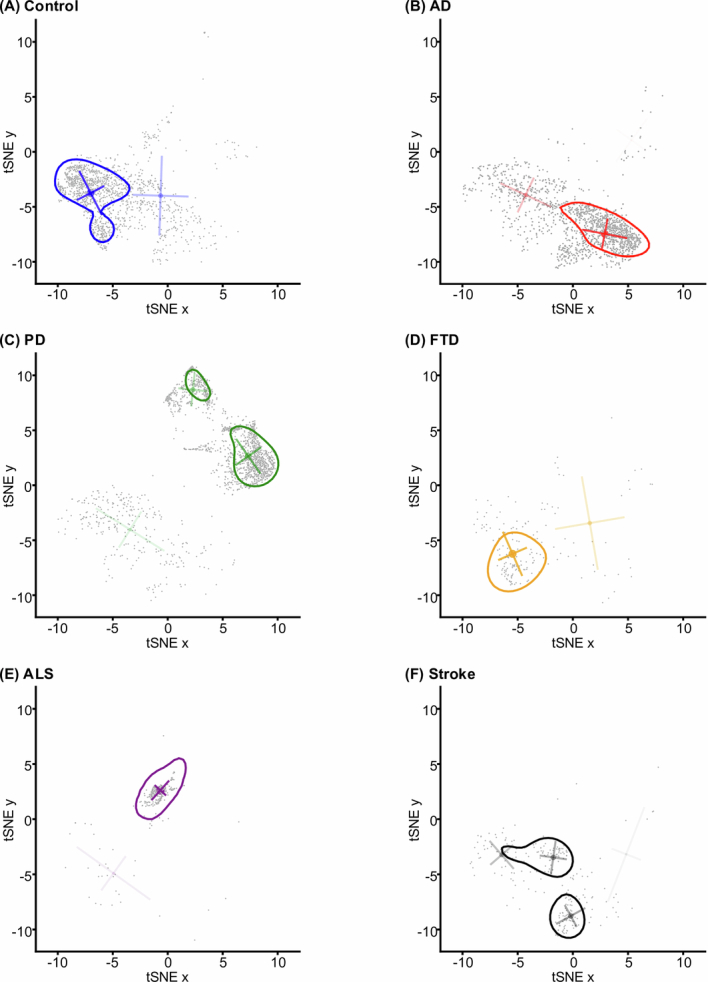
K-means clustering of participants within diagnosis on the t-SNE diagnostic probability map. The contour was drawn based on Gaussian kernel density estimation with a threshold of 0.01. (**A**) Control participants have two clusters. (**B**) AD patients have three clusters. (**C**) PD patients have four clusters. (**D**) FTD patients have two clusters. (**E**) ALS participants have two clusters. (**F**) Stroke/TIA participants have five clusters.

There were many cases distributed on the embedding into regions inconsistent with their clinical diagnosis, for example, AD cases distributed into stroke/TIA regions. This observation may result from failed model predictions, incorrect clinical diagnoses or conditions with overlapping molecular etiologies. Figure 2f shows contours onto the embeddings representing the highest density of cases for each diagnosis, as well as subclusters of case densities distributed outside of the primary density (Extended Data Fig. 2). A non-dominant cluster of healthy controls emerged at the intersection of shallow extremes of the AD and stroke/TIA regions and showed older age and higher rates of vascular/metabolic risk factors with worse cognition relative to the dominant cognitively unimpaired (CU) cluster (Fig. 2g and Supplementary Data 2). Two minor AD clusters also emerged, with one colocalizing in the stroke/TIA region and the other in the PD region, each showing distinct clinical profiles (Fig. 2h and Supplementary Data 2). Proteomically, the dominant AD cluster had higher abundance of proteins involved in cell death, damage response and mitochondrial activity but lower abundance of proteins involved in immune and defense response, whereas both minor AD clusters showed decreased abundance of proteins associated with energetic metabolism (Supplementary Fig. 3a and Supplementary Data 3 and 4). Perhaps most interestingly, a minor ALS cluster emerged closer to the FTD region and showed higher rates of *C9orf72* mutations and MCI, with differential abundance patterns consistent with upregulation of proteins relating to cell death and downregulation of proteins relating to metabolism and immunity (Fig. 2i, Supplementary Figs. 3b,c and 4, and Supplementary Data 2–4). Differential abundance and characteristics for PD, stroke/TIA and FTD are provided in Supplementary Data 3 and 4. Additional details are provided in Supplementary Results 1.

### Model interpretation highlights disease-specific networks and key discriminative proteins

**Fig. 3:**
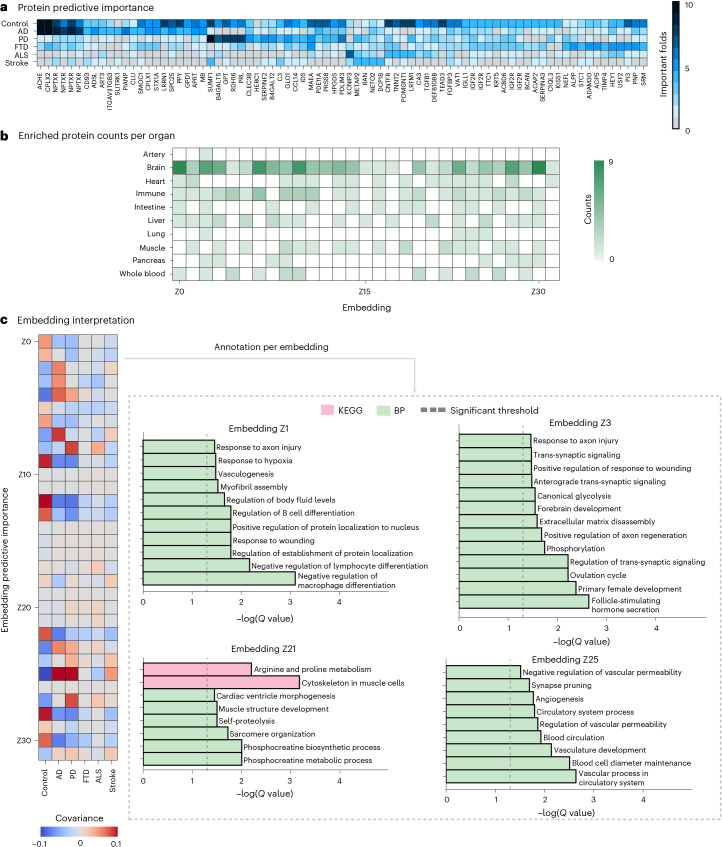
Model interpretations reveal proteomic content underlying model diagnostic predictions. **a**, Predictive importance of proteins by fold counts. Proteins were visualized if they showed significant feature importance in more than four cross-validation folds for at least one of the diagnostic tasks. **b**,**c**, Interpretations for model-derived embeddings, nonlinear low-dimensional ensembles of proteins that contribute to predictions. Frequency of organ-specific enrichment among embedding-specific proteins (**b**). Left: importance of embeddings for diagnostic tasks using covariance, a reliable feature importance metric for linear models proposed by Haufe et al.^56^. Right: GO enrichment analysis on selected embedding-specific proteins (**c**). Z1 shows signature for brain health or resilience, Z3 shows signature for AD, Z21 shows signature for both ALS and PD, and Z25 may signal vascular dysfunction.

**Extended Data Fig. 3:**
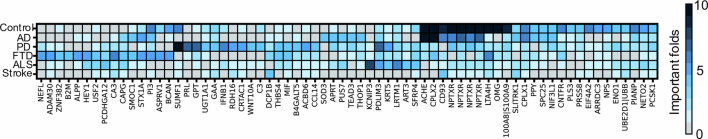
Predictive importance of proteins by cross-validation fold counts with MCI-SCI patients in the model development. Proteins were visualized if they showed significant feature importance in more than 4 folds for at least one of the diagnostic tasks.

While deep learning models are not trivial to interpret, understanding the underlying biological trends driving predictions made by ProtAIDe-Dx is essential for clinical adoption and biological insight. We used a feature permutation approach at the inference stage^23^ to identify the most discriminative proteins used by our model (Fig. 3a and Extended Data Fig. 3). Several expected and previously described proteins emerged from this analysis, such as NEFL for FTD, CPLX2, CLU and SMOC1 for AD, SUMF1 for PD and multiple NPTXR aptamers for multiple neurodegenerative diseases. Several additional proteins emerged as discriminative for different disorders with interesting and highly relevant links to brain pathology, resilience and function, described in detail in Supplementary Results 2. This list included SERPINF2, PRL, C3, GPT2 and HERC1 for PD; CNTFR, TNNT2, PMGNT1 and LRTM1 for ALS; HEY1, SERPINA1, IGF2R, MAEA and STC for both FTD and ALS; and DCP1B, METAP2 and RAN for TIA/stroke (Supplementary Results 2). Certain proteins also emerged with known relationships to drugs commonly prescribed for neurodegenerative diseases. ACHE was unsurprisingly strongly discriminative for AD, consistent with common ACHE-inhibitor treatment^24^. KCNIP3 showed the strongest ALS signal^25^, consistent with recent work linking KCNIP3 expression to treatment with riluzole^25^, a common ALS treatment. Given these findings, we compiled a library of known associations between medications and discriminative proteins identified in this analysis (Supplementary Data 5). We mapped 52 different neurodegenerative or vascular drugs associated with 12 of our discriminative proteins, although 35/52 (67.3%) mapped specifically to ACHE. Perhaps the most interesting set of proteins are those that discriminated healthy controls from all other conditions, as they may inform candidate markers of general brain health and resilience. Several proteins with known relationships to brain function or cognitive reserve emerged from this group, including GLO1, TGFB1, VAT1, STX1A, PDE11A, IGF2 and OMG^26^ (see Supplementary Results 2 for details).

**Fig. 4:**
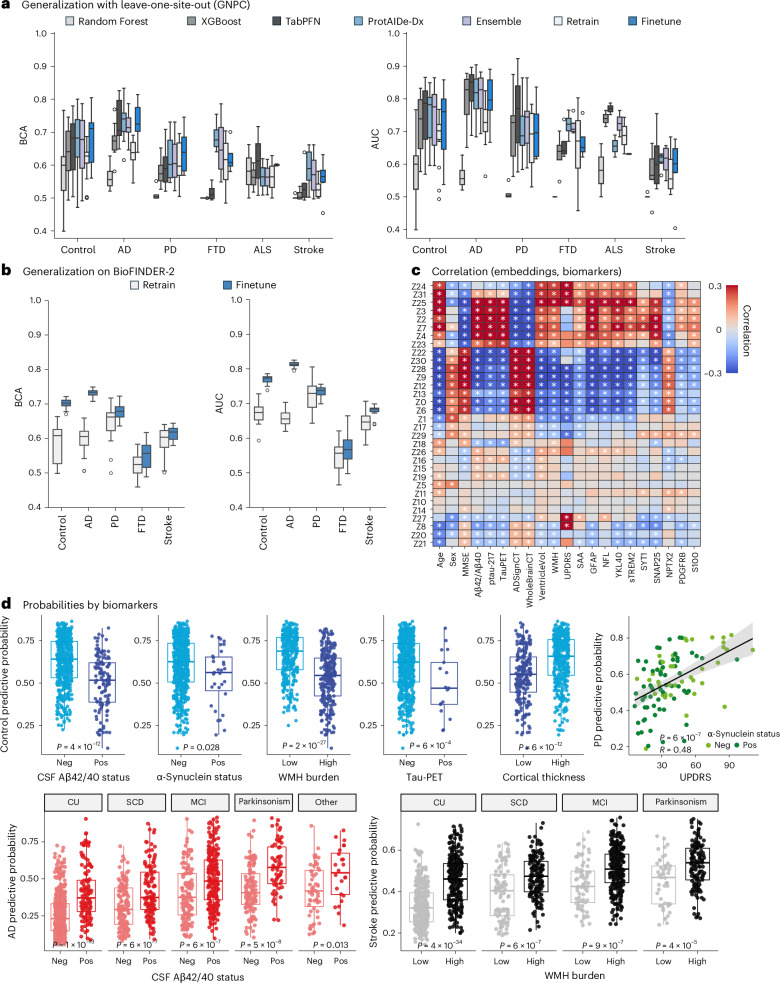
Model validation in the external BioFINDER-2 cohort. **a**, Leave-one-site-out BCAs (left) and AUCs (right) of ProtAIDe-Dx on GNPC across 14 test sites. Retrain: LR model was retrained on *K* = 100 participants’ proteins. FineTune: LR model was retrained on *K* = 100 participants’ proteomics embeddings. Two-sided corrected resample *t*-tests were applied; *P* values were FDR corrected. **b**, Model generalization performance in BioFINDER-2 cohort across 20 random *K*-shot repeats. Left: BCA. Right: AUC. Two-sided corrected resample *t*-tests were applied and *P* values were FDR corrected. **c**, Correlations between embeddings and biomarkers. A white star indicates a significant correlation after FDR correction across all comparisons. **d**, Diagnostic predictive probabilities for control (blue), AD (red), parkinsonism (PD; green) and stroke/TIA (black) diagnoses, associated with different biomarkers, stratified by biomarker-confirmed clinical diagnosis of 1,786 BioFINDER-2 participants. Neg, negative; pos, positive. The participant number of each clinical group is presented in Supplementary Table 12. Independent *t*-tests were used to compare group differences, and *P* values were corrected for multiple comparisons using FDR. All box plots in this figure show the median, interquartile range (25th to 75th percentiles) and whiskers extending to 1.5× IQR.

Next, to better understand the ProtAIDe-Dx model, we probed the proteomic composition of the model’s low dimensional embeddings. The embeddings should represent proteins that express unique nonlinear relationships in relation to neurodegenerative and/or neurological conditions and therefore may represent isolated disease-relevant molecular networks or processes (Supplementary Data 6 and 7). While we expected these embeddings to represent processes stemming from multiple organs, brain-specific proteins were highly prevalent across all embeddings (Fig. 3b). We therefore tested for enrichment of specific neural cell types (Supplementary Data 9 and 10) and triangulated this with disease discrimination (Fig. 3c) and biomarker associations in an external sample (Fig. 4c and Supplementary Data 8). We found evidence that embedding Z2 may represent neuronal functional decline, reflecting reduced resilience and synaptic dysregulation that contribute to cognitive impairment across aging and neurodegeneration. Meanwhile embedding Z23 may capture glial vulnerability pathways that link aging and sex to increased neurodegenerative disease risk (Supplemental Results 2). Other embeddings emerged with interpretable annotations helping to understand proteomic underpinnings of specific neurodegenerative diagnosis (Fig. 3c, Supplementary Data 6 and 7, and Supplementary Results 2).

### Out-of-sample generalization and validation by biomarkers of disease-specific neuropathology

**Extended Data Fig. 4:**
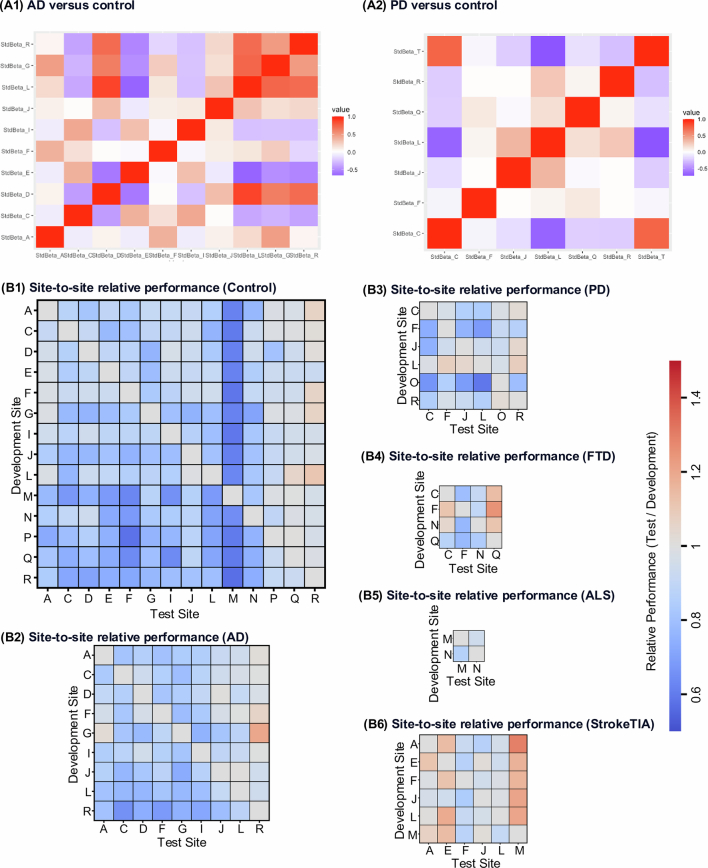
Site effects of proteomics quantified by univariate logistic regression and deep learning model TabPFN. (**A**) At each site, a univariate logistic regression was run for every protein to examine the effect of disease status (disease vs. control), with age, sex, and mean standardized protein level included as covariates; reported effect sizes correspond to the regression coefficients (β). For each pair of sites, we computed the similarity between sites by correlating their per-protein β vectors. The heatmaps show pairwise Pearson correlation values. (A1) AD versus control. (A2) PD versus control. (**B**) Site-to-site generalization performance using TabPFN. Heatmaps show the change in model performance when a TabPFN model trained on a development site is evaluated on a separate test site. Blue tones indicate poorer generalization; red tones indicate improved performance on the test site. (B1)-(B6) performances for Control, AD, PD, FTD, ALS, stroke/TIA, respectively.

We next wished to test how well ProtAIDe-Dx generalized to new datasets. Out-of-sample generalization is challenging given that within-sample performances tend to be optimistic. In a leave-one-site-out cross-validation approach, ProtAIDe-Dx continued to significantly outperform the Random Forest and XGBoost baselines and showed slight improvements over TabPFN in FTD and stroke prediction (Fig. 4a, Supplementary Table 4 and Supplementary Data 11). The ensemble model did not aid generalization performance. However, all models saw a substantial dip in performance in both balanced accuracy score and AUC compared with whole-sample cross-validation performance, probably driven by high variation in effect sizes of individual proteins across sites (Extended Data Fig. 4a,b). These performance deficits extended to additional generalization experiments (Supplementary Table 5 and Extended Data Fig. 4b). ProtAIDe-Dx’s performance was partially recovered by using finetuning (Methods).

**Extended Data Fig. 5:**
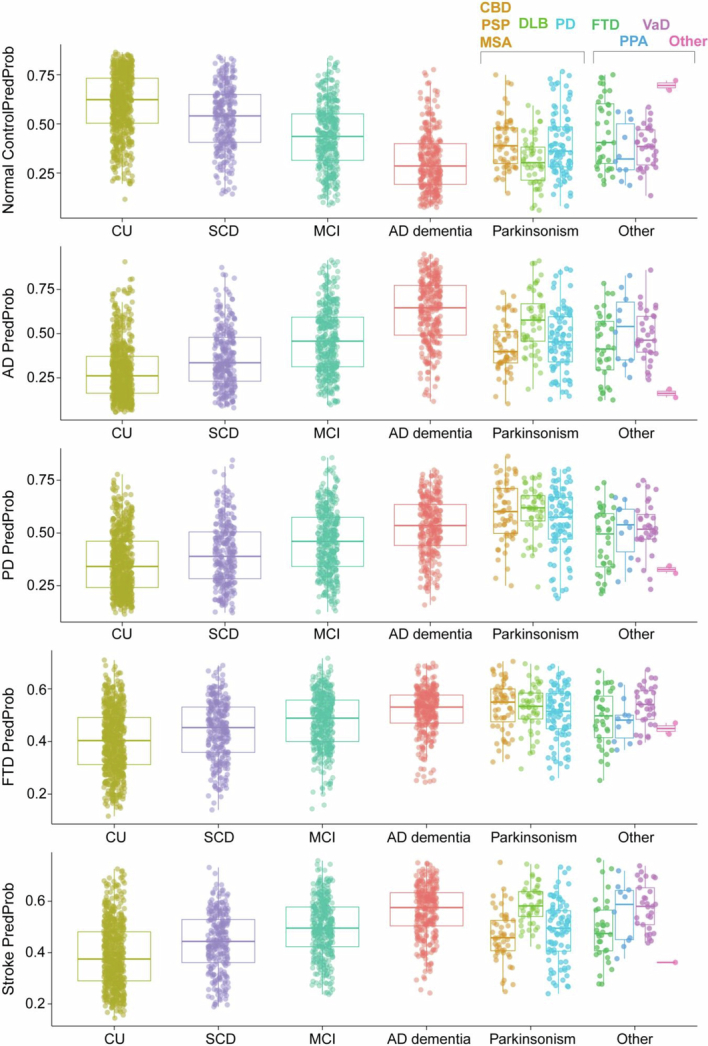
Probabilities by each diagnostic group in the BioFINDER-2 cohort. Box plots were stratified by clinical diagnostic groups among 1,786 BioFINDER-2 participants. CU (n = 609), SCD (n = 263), MCI (n = 381), AD dementia (n = 263), CBS/PSP/MSA (n = 55), DLB (n = 48), PD (n = 78), FTD (n = 33), PPA (n = 10), VaD (n = 32), Other (n = 5). Box plots show the median, interquartile range (25th–75th percentiles), and whiskers extending to 1.5×IQR.

**Extended Data Fig. 6:**
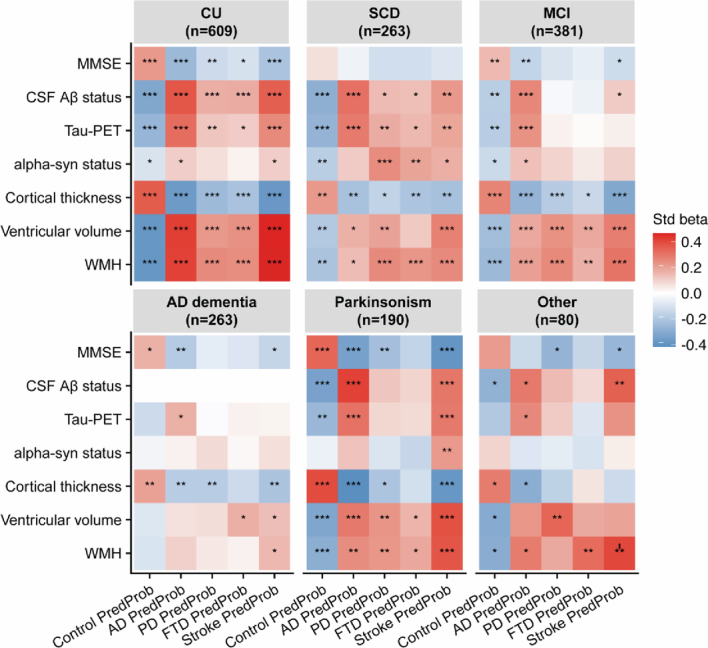
Correlations between predicted probabilities and biomarkers. Significant results from two-sided t-tests are indicated by asterisks after FDR correction across all comparisons: * FDR-adjusted P = 0.01–0.05; ** FDR-adjusted P = 0.001–0.01; *** FDR-adjusted P < 0.001. Detailed correlations and FDR-corrected p values can be found in SuppData 12.

We next applied ProtAIDe-Dx to the BioFINDER-2 dataset (*N* = 1,786), a real-life memory clinic dataset with biomarker-supported diagnosis. Note that, while BioFINDER-2 is part of GNPC, this site was excluded from model fitting. Diagnostic performance was close to the median of general leave-one-site-out performance (Fig. 4b). Predicted probabilities across diagnosis revealed expected trends (Extended Data Fig. 5); PD probabilities were elevated in patients with PD but also in dementia with Lewy bodies (DLB) cases, and stroke/TIA probability was elevated in patients with vascular dementia. Subsequently, we tested whether these probabilities correlated with disease-specific biomarkers within disease groups (Fig. 4d, Extended Data Fig. 6 and Supplementary Data 12). Among CU individuals, the model-derived probability of being CU was lower for participants expressing AD, Lewy body or neurovascular pathology (Fig. 4d). This indicates that some ‘false positive’ results from ProtAIDe-Dx may correctly identify underlying preclinical neuropathology (Supplementary Table 6). Similarly, AD probabilities were higher in non-AD cases with comorbid Aβ and Tau pathology (Fig. 4d and Supplementary Fig. 6), and higher stroke/TIA probabilities were associated with greater white matter hyperintensity (WMH) burden in both impaired and unimpaired individuals (Fig. 4d). PD probabilities did not show a significant relationship with presence of Lewy body pathology (as measured using CSF α-synuclein seed amplification assays (SAA)) but were correlated with symptom progression (Unified Parkinson’s Disease Rating Scale (UPDRS)) in PD cases (Fig. 4d).

### Proteomics provide additive information to diagnosis in a memory clinic sample

**Fig. 5:**
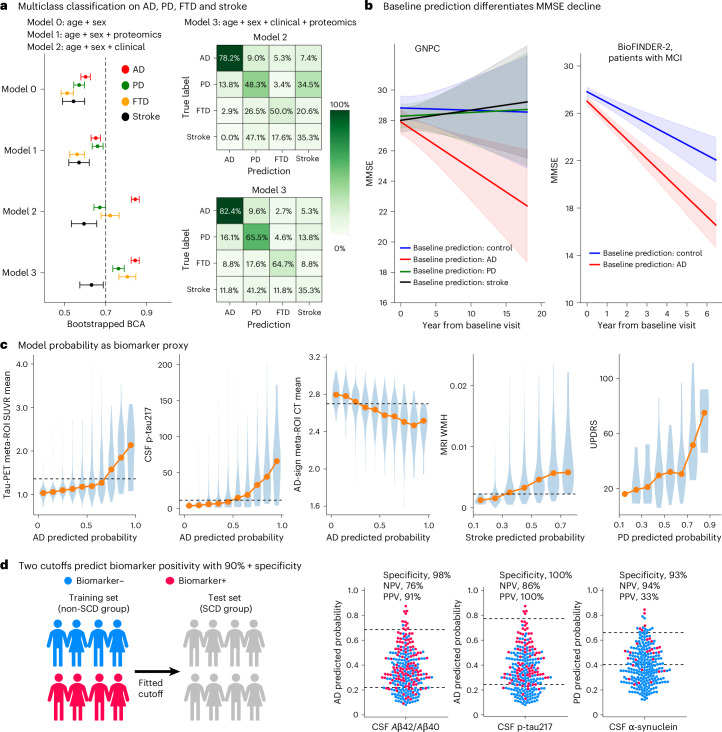
Clinical utility of ProtAIDe-Dx. **a**, Multi-class classification of patients with AD, PD, FTD and stroke using various models inclusive of demographics, ProtAIDe-Dx embeddings and/or common clinical biomarkers. Left: one-versus-rest BCA computed by 1,000 bootstraps on testing patients. Right: confusion matrices on testing patients for model 2 (top) and model 3 (bottom). Error bars represent the s.d. across 1,000 bootstrap resamples. **b**, Left: model-predicted baseline diagnoses differentiated longitudinal MMSE trajectories of GNPC patients irrespective of true baseline clinical diagnosis. Trajectories were modeled using linear mixed-effects model of MMSE with Age, Sex, Site, BaselineDx, BaselinePredictedDx, Year and the BaselinePredictedDx × YearInteraction, with subject-specific random effects for SubjectID. Right: replication on BioFINDER-2 patients with MCI. Solid lines indicate linear mixed-effects model-predicted mean MMSE trajectories, and shaded regions represent 95% confidence intervals for the linear mixed-effects fixed-effects predictions. **c**, Biomarker distributions across predicted probability bins. CT, cortical thickness. **d**, Two-cutoff strategies for predicting biomarker positivity. Cutoffs were derived from non-SCD BioFINDER-2 participants (*n* = 1,524) to achieve 90% NPVs and PPVs, except for α-synuclein, where the PPV was set at 40% owing to sample validity constraints. These fit cutoffs were then applied to patients with SCD (*n* = 263) to estimate accuracy in this clinically relevant sample. Icons in **d** created in BioRender; An, L. https://biorender.com/q2by4y5 (2026).

For models such as ProtAIDe-Dx to be translated to real-life clinical applications, it is important to show evidence that they provide additive value in these settings. Using the same external BioFINDER-2 dataset, we fit a series of models seeking to identify primary etiological diagnosis using a baseline model of only age and sex (model 0), using just ProtAIDe-Dx and demographics (model 1), using accessible clinical markers (model 2: demographics, MMSE, mean cortical thickness of AD-signature meta-region of interest (ROI) (ADSignCT^27^), plasma p-tau217 and plasma NEFL) and using all of these markers together (model 3). The final model incorporating ProtAIDe-Dx with common clinical biomarkers (model 3) achieved significantly higher BCA than the model using only common clinical markers (model 2), especially adding value in diagnosis of non-AD dementias (Fig. 5a and Supplementary Tables 7 and 8). We also performed an analysis distinguishing subtypes of Lewy body disease (Supplementary Results 3).

**Extended Data Fig. 7:**
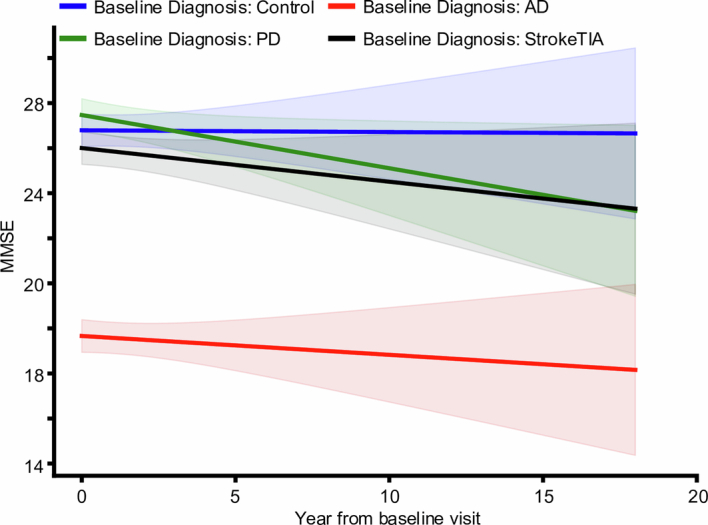
MMSE longitudinal trajectories by baseline clinical diagnosis in the GNPC. Longitudinal trajectories were assessed using the linear mixed-effects model: MMSE ~ Age + Sex + Site + BaselineDx × Year + (Year | SubjectID). Fixed effects: Age, Sex, Site, and the interaction between baseline diagnosis and time (Year); random intercepts and slopes for Year were included for each subject. The BaselineDx × Year term tests whether the annual change in MMSE differs by baseline diagnosis. Solid lines indicate linear mixed-effects model predicted mean MMSE trajectories, shaded regions represent 95% confidence intervals for the LME fixed-effects predictions.

Despite being trained exclusively on baseline visits, ProtAIDe-Dx demonstrated the ability to differentiate longitudinal rates of cognitive decline. Baseline clinical diagnoses did not distinguish rates of decline after FDR correction in the GNPC (*P* > 0.05 across all diagnostic groups; Extended Data Fig. 7 and Supplementary Table 9). However, baseline predicted diagnosis from ProtAIDe-Dx did significantly stratify decline trajectories (FDR-corrected *P* value <0.05 across all prediction groups; Fig. 5b(1) and Supplementary Table 10), independent of clinical diagnosis. Similarly, in the BioFINDER-2 dataset, patients with MCI predicted as AD by ProtAIDe-Dx declined more rapidly than patients with MCI predicted as control (FDR-corrected *P* value of 0.0015; Fig. 5b(2) and Supplementary Table 11).

The probability outputs from ProtAIDe-Dx provide clinically interpretable indicators of biomarker status. In the external BioFINDER-2 dataset, we found that when the AD probability exceeded 0.9, most patients were tau positive by Tau-PET (first graph) or CSF p-tau217 (second graph) and showed cortical thickness below the diagnostic threshold (Fig. 5c, third graph). Likewise, when the stroke probability exceeded 0.7, most patients exhibited elevated white matter hyperintensities (fourth graph). A two-cutoff strategy derived on participants without SCD achieved >90% specificity and positive predictive value (PPV) in patients with SCD (Fig. 5d). This approach achieved 94% negative predictive value (NPV) for detecting LBD-related biomarker positivity on the basis of CSF α-synuclein (see Supplementary Fig. 7 for models ensuring 50% coverage or maximizing PPVs and NPVs).

### Proof-of-concept diagnostic report by ProtAIDe-Dx

**Fig. 6:**
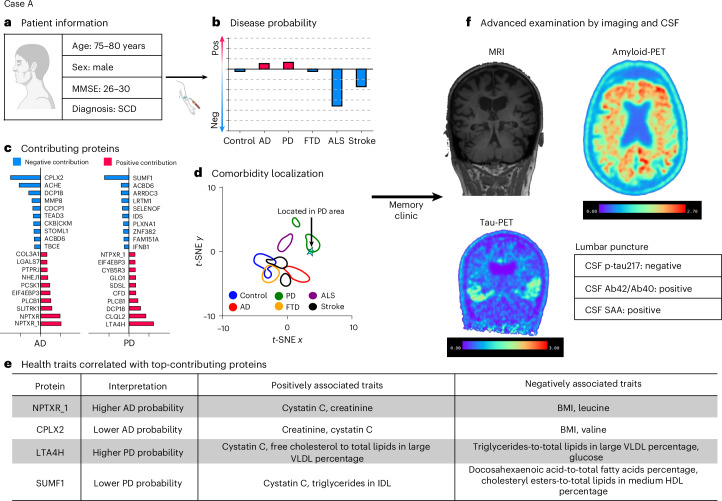
Individual neurodegeneration risk report (case A). **a**, Demographics and cognition score of one older participant who was diagnosed with SCD. **b**, ProtAIDe-Dx predicted this participant with higher probabilities of AD and PD. The probabilities were normalized to be centered at zero. **c**, Contributing proteins for the decision were computed on the basis of SHAP values. The top 10 positively and negatively contributing proteins were visualized. **d**, The location of this participant on the diagnostic probability map indicates his proteomic signature was consistent with typical patients with PD. **e**, Health traits correlated with top contributing proteins were listed, informing clinicians to pay attention to these traits. BMI, body mass index; HDL, high-density lipoprotein; IDL, intermediate-density lipoprotein; VLDL, very low-density lipoprotein. **f**, A signature such as this would be suggestive of follow-up advanced imaging and CSF biomarker examination at a memory clinic to confirm the diagnosis. Here, AD pathology was confirmed by both a positive β-amyloid-PET scan and a positive tau-PET, as well as a positive CSF Aβ42/Aβ40 ratio, but not positive CSF p-tau217 levels, interestingly. The presence of underlying Lewy body pathology was confirmed through a positive CSF seed-amplification assay for α-synuclein pathology. Illustration in **a** created in BioRender; An, L. https://biorender.com/q2by4y5 (2026).

**Extended Data Fig. 8:**
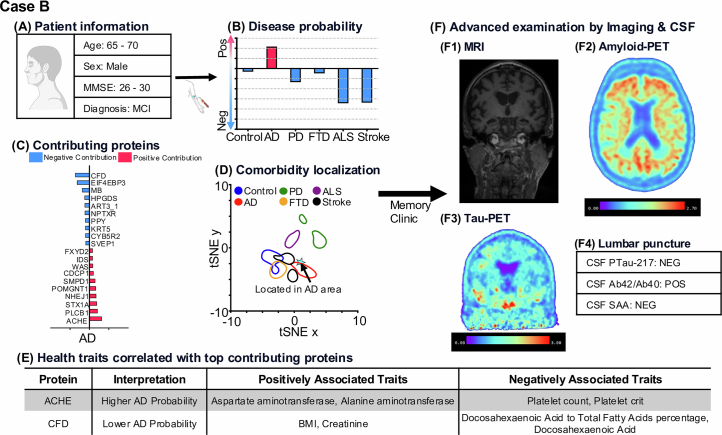
Individual neurodegeneration risk report (Case B). (**A**) Demographics and cognition score of one older participant, who was diagnosed with MCI. (**B**) ProtAIDe-Dx predicted this participant with higher probabilities of AD. The probabilities were normalized to be centered at zero. (**C**) Contributing proteins for making the decision were computed based on SHAP values. The top 10 positive and top 10 negative proteins were visualized. (**D**) The location of this participant on the diagnostic probability map indicates he was close to typical AD patients. (**E**) Health traits correlated with top contributing proteins were listed, informing clinicians to pay attention to these traits. (**F**) Advanced imaging and CSF biomarker examination at the memory clinic to confirm AD pathology. Illustration in **a** created in BioRender; An, L. https://biorender.com/q2by4y5 (2026).

**Extended Data Fig. 9:**
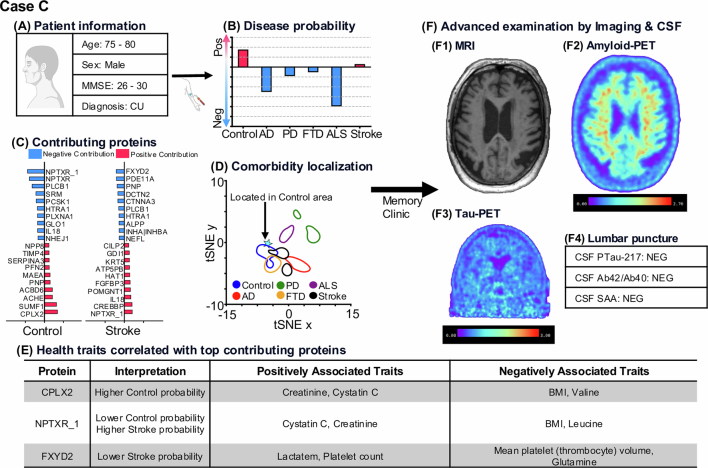
Individual neurodegeneration risk report (Case C). (**A**) Demographics and cognition score of one older participant, who was diagnosed with MCI. (**B**) ProtAIDe-Dx predicted this participant with higher probabilities of AD. The probabilities were normalized to be centered at zero. (**C**) Contributing proteins for making the decision were computed based on SHAP values. The top 10 positive and top 10 negative proteins were visualized. (**D**) The location of this participant on the diagnostic probability map indicates he was close to typical AD patients. (**E**) Health traits correlated with top contributing proteins were listed, informing clinicians to pay attention to these traits. (**F**) Advanced imaging and CSF biomarker examination at the memory clinic to confirm AD pathology. Illustration in **a** created in BioRender; An, L. https://biorender.com/q2by4y5 (2026).

The clinical utility analysis shows the potential of ProtAIDe-Dx to provide significant additive information to clinical diagnostic workup. We therefore built a proof of concept for a diagnostic report using ProtAIDe-Dx (Fig. 6 and Extended Data Figs. 8 and 9). The report indicates diagnostic probability across all conditions (Fig. 6b) and includes a localization of the patient to the GNPC disease probability space (Figs. 2a and 6d). Importantly, the report uses model explanation technology to report which proteins contributed to this specific individual’s prediction (Fig. 6c), allowing a clear biological explanation of the proteomic diagnosis. Physical traits linked to these patient-specific diagnostic proteins are also provided by programmatically accessing a library of protein–trait associations (Fig. 6e), providing possibility for lifestyle interventions or further explanation. We use three patient case examples to showcase the report. Case A (Fig. 6) was a 75–80-year-old man entering the memory clinic with subjective cognitive complaints but objectively intact cognition. ProtAIDe-Dx predicted underlying comorbid AD and Lewy body pathologies. This was confirmed by positron emission tomography (PET) scans showing cortical Aβ burden and temporal tau pathology (AD), as well as positive cerebrospinal fluid (CSF) SAA indicating Lewy body pathology (Fig. 6f). Supplementary Results 4 provides information about the other case studies. While not all ProtAIDe-Dx probabilities were accurate and confirmed by biomarkers (Fig. 4b,d), these examples showcase the potential of ProtAIDe-Dx in adding explainable proteomic data informative toward neurodegenerative etiology to clinical workup.

## Discussion

The rapid growth of dementia populations worldwide urges scalable and accessible biomarkers for neurodegenerative diseases. However, while promising biomarkers for certain diseases are on the horizon^28–30^, many biomarkers under development are either invasive (CSF) or not yet scalable and are usually singular, requiring multiple tests to assess different pathologies. Therefore, a one-shot, multi-disease biomarker panel that is minimally invasive, economical and widely accessible is highly anticipated. This study presents an early attempt toward this goal by applying deep neural networks on a massive (*N* = 17,187) neurodegenerative disease sample and high-rank (7,595 proteins) plasma proteomics data. Our proposed ProtAIDe-Dx model synthesized novel biological insight and exhibited clinical utility by mining neurodegeneration-related signals from high-rank proteomics data at multiple levels. Moreover, when generalized to an out-of-sample memory clinic dataset, ProtAIDe-Dx improved automated differential diagnosis accuracy beyond the capability of currently accessible clinical biomarkers. This study distinguishes itself from other neurodegenerative disease proteomics studies owing to its focus on making individual-level predictions on new cases, by training a model to recognize multiple copathologies rather than on differential diagnosis, and by rigorously avoiding leakage and overfitting and providing true generalization performance. However, despite providing complementary information capable of enhancing current clinical workup, our data suggest that contemporary high-throughput plasma proteomics assays alone cannot yet replace currently available clinical markers. Altogether, this study sets a benchmark for future proteomics studies by providing a baseline for predictive performance, a resource sorely needed in the biomedicine AI field^31^.

The performance of ProtAIDe-Dx is not sufficient at present to replace currently available clinical markers, although we demonstrate several examples of how it could be integrated into clinical practice as an accessible and cost-effective assistant. Disease-differentiation experiments indicated that ProtAIDe-Dx provides additive value beyond currently available clinical biomarkers, highlighting its clinical potential given the high prevalence of neurodegenerative copathologies in the aging population and the fact that affordable, minimally invasive biomarkers for PD and FTD are still under development^29,30^. ProtAIDe-Dx’s predictions to differentiate longitudinal rates of cognitive decline may offer a useful tool for clinical management, as individuals predicted as having AD tended to exhibit a faster decline irrespective of their current diagnostic status. These findings suggest that diagnostic labels may not fully reflect underlying disease progression, whereas ProtAIDe-Dx tends to capture more informative biological signals that could enhance clinical decision making. Probability outputs from ProtAIDe-Dx also provide clinically interpretable indicators of biomarker status, consistent with established approaches such as the Amyloid Probability Score 2 (APS2) developed by C2N Diagnostics. Higher predicted probabilities generally reflected greater biomarker burden, and the two-cutoffs approach achieved >90% specificity for determining biomarker positivity. Intermediate predicted probabilities may suggest early pathology and should prompt confirmatory assessment with other established biomarkers.

Owing to its diverse site composition and unstandardized data collection, the GNPC creates an excellent dataset for building and testing tools for translation to real-world clinical settings, where data curation and standardization rarely match that of most research datasets. Within the GNPC, ProtAIDe-Dx achieved impressive cross-validated diagnostic accuracy across diseases, especially for ALS and PD. However, this performance decreased in a leave-one-site-out validation setting and a site-to-site generalization setting that more closely simulates a true clinical translation, especially for disease with high imbalanced distribution across sites (for example, patients with ALS mainly come from one site). This indicates that, despite the large and diverse sample, site effects and overfitting remain a challenge for training generalizable models. This cannot necessarily be explained by the overcomplexity of our deep learning architecture, as ProtAIDe-Dx consistently outperformed the less complex Random Forest and XGBoost models. It is likely that advances in generalizable data harmonization^32^ or standardization^33^ will be necessary to overcome these limitations.

In general, several factors must be considered when evaluating the diagnostic performance of ProtAIDe-Dx. One of these factors is the difficulty of the task. For instance, FTD is a highly heterogeneous disorder with multiple underlying pathologies and many different clinical presentations^34^, and stroke/TIA is a stochastic event and only of many manifestations of neurovascular disease^35,36^, and itself may often go undiagnosed. However, one of the most important considerations in evaluating the performance of prediction models in neurological diseases relates to the accuracy of the original clinical diagnosis, which both forms the basis of training and serves as the standard for evaluation. Neurological and neurodegenerative diseases are notoriously difficult to diagnose^37^, and in our particular case, models were trained and evaluated on the basis of clinical diagnoses that, for the most part, lacked biomarker confirmation. In addition, the clinical diagnosis criteria varied across sites, given that GNPC is a retrospective collection of multiple cohorts. Given that there are multiple criteria for the diagnosis of AD^38,39^ and multiple interpretations of those criteria, this issue will be present in any real-world application. In light of these limitations, many ‘false’ predictions from ProtAIDe-Dx may not represent incorrect diagnoses, considering the prevalence of asymptomatic disease, co-pathology and misdiagnosis. Along similar lines, we also observed patients with the same diagnosed clinical syndromes but who demonstrate distinct proteomic profiles. These findings are in line with a growing number of studies finding multiple proteomic or transcriptomic subtypes within the disease populations^40,41^. Together, these observations demonstrate that there is a set of neurodegenerative signatures detectable in blood plasma that may be associated with differing clinical profiles or that may represent stratified responses to general neurological insult. In either case, a patient’s underlying biological response to pathology may be just as relevant to treatment and prognosis as their clinical presentation. Future work should leverage longitudinal data to further explore the clinical meaningfulness of these proteomic disease subgroups.

Beyond producing diagnostic probabilities for multiple diseases, ProtAIDe-Dx also identified a compact set of top predictive proteins that significantly influenced model performance on out-of-sample data. While many previous studies have described lists of differentially abundant proteins associated with different diseases^36,42–44^, predictive modeling is probably more clinically useful for making individual diagnoses^45^. In our study, among the top proteins discriminating different conditions, those proteins that discriminated controls from all neurodegenerative conditions were perhaps the most interesting. GLO1, PDE11A and IGF2 have all been investigated as cognition enhancers in various aging models^46,47^ and may indeed be promising targets given that they also signal healthy aging in our large human cohort. Many of the proteins profiled were brain expressed and played a role in either memory (PDE11A and IGF2), inflammation (TGFB1), neurite regeneration (OMG) or endosomal/lysosomal systems (STX1A). OMG was a highly discriminative protein for controls specifically when differentiating them from not just dementia but also patients with MCI–SCI, suggesting its possible role in maintenance of cognition. Meanwhile, lower expression levels of NPTXR in neurodegenerative groups have been observed in multiple studies^48,49^, highlighting synapse loss as a common feature of neurodegeneration^50^. These proteins should continue to be investigated as markers of multi-cause neurological malaise, which might be useful in triaging or early diagnosis and monitoring. Further studies should continue to investigate how these proteins change over time in response to other outcomes of brain health.

In all, ProtAIDe-Dx showed great promise in disease diagnosis, probing disease heterogeneity, identifying novel proteins of interest and generating health- and disease-related signatures. However, there are still challenges that we wish to highlight to facilitate future studies exceeding the performance of ProtAIDe-Dx in real-world samples. A major challenge of ProtAIDe-Dx is that its diagnostic accuracy has not yet reached the level required for standalone clinical use. This limitation may be due to several factors. The performance of ProtAIDe-Dx was comparable to that of other studies using UK Biobank data^51,52^, suggesting that there may be a performance ceiling of high-throughput plasma proteomics as biomarkers. Another factor might be a limited set of specific aptamers that target arbitrary protein conformations, which are also secreted or surface based and are detectable in blood. This is a particular challenge for brain diseases, as many disease-relevant proteins are probably brain expressed and many of these do not cross the blood–brain barrier. In addition, plasma p-tau biomarkers have achieved much better performance discriminating AD than we found here^7,53^ owing to the identification of specific peptides and post-translational modifications directly related to disease pathology. To achieve similar improvements, mass spectrometry and/or other approaches may be needed for more comprehensive screening of peptides and protein fragments^54^. Another challenge of ProtAIDe-Dx is its relatively poor site generalization performance, which hinders its applicability to new sites. Strong site effects in plasma proteomics^55^ may limit generalization and could be mitigated by harmonization^32^. As discussed, the diagnostic labels that our models were trained on may not be sufficiently reliable owing to the lack of biomarker support and imperfect harmonization of diagnostic criteria across cohorts. To address this issue, integrating plasma proteomics studies conducted during life with neuropathological assessments at death may provide an eventual solution for enabling model training with ground truth diagnostic labels. Alternatively, future studies are recommended to incorporate more biomarker-confirmed samples to enable models to more accurately capture biologically meaningful signals for disease classification. Another challenge is presented by the potential confounding effects of various factors on protein levels. For example, medication use can significantly affect circulating protein levels, sometimes exceeding normal physiological ranges^24^, and may therefore dominate model predictions. Hopefully, many of these challenges can be addressed or improved in future releases of GNPC^18^.

In summary, ProtAIDe-Dx represents a pioneering attempt toward the development of scalable, minimally invasive and multi-disease diagnostic tools for neurodegenerative diseases. Despite its promise, predictive proteomics as a field faces several challenges, including insufficient diagnostic accuracy for clinical deployment, limited generalizability across sites and potential confounding effects. Future work should address these limitations by incorporating more reliable diagnostic labels to improve accuracy, refining model architecture to enhance generalization and implementing strategies to mitigate confounding biases. Overall, ProtAIDe-Dx establishes a robust benchmark for AI-driven proteomics tools, paving the way for precision medicine in neurodegenerative diseases.

## Methods

### Datasets

#### GNPC

The GNPC (https://www.neuroproteome.org/), launched in 2023, is a mega consortium combining multiple dementia and population cohorts, including healthy aging, AD, PD, FTD, ALS and stroke/TIA^18^. All cohorts and data were anonymized. Ethics approval for each individual cohort was obtained from their respective institutional review boards. All participating cohorts confirmed that informed consent was obtained from all individuals contributing clinical and generated biosample data before contributing data to the GNPC. The latest GNPC v1.3MS release collected 20,532 participants from 22 contributors (sites), 3,950 of whom had longitudinal visits. We used baseline visit proteomics data for model development.

In this study, we selected 17,187 participants on the basis of SomaLogic 7k proteomics availability. Site U’s proteomics data were from serum but we kept site U to maximize the sample size and learn modality-agnostic signatures. For the 9,708 participants that were neither diagnosed as control nor with any of the five above mentioned diseases, we visualized the distribution of these participants (Supplementary Fig. 8). On the basis of MMSE and CDR, we mapped participants with good cognition (MMSE ≥26 or CDR of 0) as ‘control’ and participants with MCI (20 ≤ MMSE ≤ 25 or CDR of 0.5) as ‘MCI–SCI’. Among the remaining 1,606 participants, 1,062 participants with poor cognition (MMSE ≤10 or CDR ≥1) were labeled as ‘ComputedDementia’, and 542 participants without valid MMSE or CDR were labeled as ‘Unknown’. Supplementary Table 1 presents the demographics and cognition (MMSE) distribution of 17,187 participants by site, and the corresponding distribution of clinical diagnoses is presented in Supplementary Table 2. Race and ethnicity information is shown in Supplementary Fig. 9. It is noted that diagnoses of some sites were confirmed by biomarkers but not for most sites. In the following analysis, the patients with either stroke or TIA were labeled as ‘Stroke’ in figures for ease of visualizations but are referred to as ‘stroke/TIA’ in the main text.

All GNPC blood samples were shipped separately by each contributor and were analyzed by SomaLogic, coordinated by Gates Ventures. SomaLogic applied slow off-rate modified aptamer (SOMAmer) technology, in which chemically modified nucleotides enable high-specificity and high-affinity binding to target proteins. The resulting proteomic data were standardized, normalized and calibrated, with protein abundances reported in relative fluorescent units. Before integration into the GNPC cohort dataset, aptamers were mapped to UniProt^18^. We removed the outlier values for each protein that exceeded six standard deviations (s.d.).

#### BioFINDER-2 cohort

The Swedish BioFINDER-2 dataset (https://biofinder.se/two/, NCT03174938) is a prospective cohort in the south of Sweden spanning the full continuum of AD as well as including patients with non-AD neurodegenerative diseases. Participants are deeply phenotyped, including clinical assessment, CSF/blood sampling, PET and magnetic resonance imaging (MRI) imaging data. All studies were approved by the Institutional Review Board of Lund University and written informed consent or assent was obtained from all participants or their legally authorized representative. BioFINDER-2 is a participating site in GNPC but, in a subanalysis aiming to evaluate the disease probability predicted by the different models in relation to key markers of AD and neurodegenerative diseases, we focused specifically on this cohort. For the BioFINDER-2 subanalysis, we selected 1,786 participants with plasma SomaLogic 7k proteomics data. The demographics and cognition distribution of 1,786 participants are presented in Supplementary Table 12.

We grouped participants into six different groups: CU, SCD, MCI, AD, parkinsonism and other diseases. The BioFINDER-2 dataset inclusion and exclusion criteria have been described in detail previously^57^. In brief, CU participants needed to have an MMSE score of at least 27 (if <66 years old) or 26 (if ≥66 years old) and no signs of cognitive symptoms as assessed by physicians specialized in cognitive disorders. Participants with SCD or MCI were all referred to a memory clinic owing to cognitive symptoms, had an MMSE score between 24 and 30 and did not fulfill criteria for any dementia according to the Diagnostic and Statistical Manual of Mental Disorders, Fifth Edition (DSM-5). Participants were classified as MCI if they performed at least 1.5 s.d. below the normative score on at least one cognitive domain from an extensive neuropsychological test battery^12^, while participants with SCD performed better than −1.5 s.d. Patients with dementia fulfilled the DSM-5 criteria for dementia, and all patients with AD dementia were Aβ-positive (based on CSF Aβ42/Aβ40). Patients with non-AD neurodegenerative diseases were also included in the cohort. Patients with PD, DLB and atypical parkinsonism disorders formed the group ‘parkinsonism’. Patients with other diseases were grouped together (labeled ‘Other’) and included patients with FTD spectrum disorders, vascular dementia and one patient with etiology not otherwise specified. Clinical diagnosis of AD dementia or other neurodegenerative diseases was determined by experienced clinicians.

To benchmark generalization performances on BioFINDER-2 (Fig. 4b) against leave-one-site-out on GNPC, we performed additional diagnostic grouping to match GNPC on the basis of clinical diagnosis and biomarkers of BioFINDER-2. Participants with a clinical diagnosis of normal were labeled as control; patients with an abnormal CSF Aβ42/Aβ40 ratio and a clinical diagnosis of AD were labeled as AD; patients with a clinical diagnosis of PD, DLB or parkinsonism (not otherwise specified) were labeled as parkinsonism disease (PD); patients with a clinical diagnosis of behavioral variant FTD, semantic variant of primary progressive aphasia, FTD (not otherwise specified) or an SCD with a MAPT mutation were labeled as FTD; and patients with infarcts were labeled as stroke. It is noted that stroke is not exclusive to other diagnosis categories. For example, a patient with AD might also be diagnosed with stroke. In summary, we obtained 609 control participants, 261 patients with AD, 135 patients with PD, 43 patients with FTD and 117 patients with stroke.

##### Biomarkers of interest in BioFINDER-2

Multiple biomarkers and the MMSE as a global measure of cognition were investigated in BioFINDER-2. The AD biomarkers were Aβ status (based on CSF Aβ42/Aβ40) and tau-PET standardized uptake value ratio (SUVR) in a temporal meta-ROI (tracer ^18^F-RO948)^58^. The positivity of CSF p-tau217 was determined at a cutoff of 11.42 pg ml^−1^ (ref. ^12^). Structural MRI markers of interest were the cortical thickness in an AD signature composed of temporal lobe regions^27^, whole-brain cortical thickness and ventricular volume (average of lateral ventricle volume in both hemispheres divided by total intracranial volume). T1-weighted MRI was processed with FreeSurfer v6.0. White matter hyperintensity burden (divided by total intracranial volume) was measured on the basis of fluid-attenuated inversion recovery and T1 images processed with Sequence Adaptive Multimodal Segmentation (SAMSEG) tool from FreeSurfer v7.1. α-Synuclein status was available from seed amplification assay performed in the CSF, as described previously^12^. For a subset of participants in the parkinsonism group (*n* = 100), the UPDRS was also available.

### Model cross-validation and generalization

After diagnostic label mapping, 11,803 participants were labeled as either control or one of the five aforementioned diseases, 662 participants diagnosed with AD but exhibiting healthy cognitive scores (MMSE ≥26) were labeled as ‘HealthyAD’, 3,116 participants were labeled as ‘MCI–SCI’, 1,064 were labeled as ‘ComputedDementia’ with poor cognitions (MMSE <19 or CDR ≥1) and 542 were labeled as ‘Unknown’. The 11,803 participants in the control and disease groups were used as the development set for model training and evaluation, including tenfold cross-validation and leave-one-site-out procedures. The ‘HealthyAD’, ‘MCI–SCI’, ‘ComputedDementia’ and ‘Unknown’ groups were held out as additional test sets. We also conducted a supplementary experiment by including patients with MCI–SCI in model development (Extended Data Fig. 3).

We conducted a tenfold cross-validation procedure to evaluate the performances of ProtAIDe-Dx models on GNPC. Participants from each site were evenly split into ten folds, so each fold contained data from all sites. A 9–1–1 train–validation–test split was used, with 8 of 10 folds as a training split, one fold as a validation split and the remaining one as a test split. This process was repeated ten times, with each fold serving as the test split once.

To test the site generalization performance of the proposed ProtAIDe-Dx models, a leave-one-contributor-out scheme was used. Data from one site were reserved as the test split, while the data from the remaining contributors served as a training–validation split.

The sites used as test sets were selected on the basis of the following criteria: for any of the six conditions, (1) this site had 200 or more participants with non-missing diagnoses and (2) this site had at least five participants with minor diagnosis categories for this condition. In total, 14 testing sites fit this criteria. Detailed information is presented in Supplementary Table 13. Notably, the BioFINDER-2 cohort was part of GNPC; therefore, we excluded BioFINDER-2 participants when training and tuning the ProtAIDe-Dx model for testing on BioFINDER-2.

### Machine learning model development

#### Feature selection

A feature selection procedure was used to reduce the number of input proteins. On each training and validation split, we conducted both GLM association analysis and XGBoost^20^ predictive analysis to select informative proteins as input features. As GLM does not require any hyperparameters to set, we merged training and validation participants together to run the GLM association analysis for each of the six conditions and each of the 7,595 SomaLogic 7k proteins following

1 $$\mathrm{Protein} \sim \mathrm{Condition}+\mathrm{Age}+\mathrm{Sex}+\mathrm{AverageProteinLevel}.$$

AverageProteinLevel is the average protein level across all 7k proteins to control for individual expression differences^59^.

After running 7,595 GLMs for each target, we first selected proteins with fold change of beta Condition >2 or <0.5 and then picked the top 5 proteins with the smallest corrected *P* values of beta Condition.

We also ran XGBoost predictive models on each training and validation split to select predictive proteins. We merged training and validation participants together and resplit these participants into ten subfolds, nine used for training models and one as validation split to tune model hyperparameters. This procedure was repeated ten times to predict each condition, with each subfold serving as the validation split once. After all ten models for each condition were trained, proteins internally selected for building models by all ten XGBoost models were kept.

In this way, the number of input proteins was reduced from 7,595 to 200–300 proteins as input features, which vary across each train–validation–test split. In total, 738 proteins were kept across ten train–validation–test splits. This feature selection procedure was performed for both cross-validation and leave-one-contributor-out experiments.

#### Classification metrics

Accuracy is an inappropriate and potentially misleading metric given the imbalanced distribution across the six conditions. We therefore chose BCA as classification metric. We also reported AUC scores for reference.

#### Random Forest as baseline approach

Random Forest models were used as baseline machine learning approaches to classify binary targets separately. We therefore trained six Random Forest models for classifying six binary conditions. The input proteins to Random Forest models were *z*-normalized by mean and s.d. computed from the training split. To get optimal validation prediction accuracies, a grid search on hyperparameters, including maximum tree depth, number of features for best split and probability threshold, was performed on the validation split. The Random Forest model trained with optimal hyperparameters was then applied to the test split. This procedure was repeated for each training–validation–test split, including cross-validation and leave-one-site-out.

#### XGBoost as baseline approach

XGBoost models were used as baseline machine learning approaches to classify binary targets separately. We therefore trained six XGBoost models for classifying six binary conditions. The input proteins to XGBoost models were *z*-normalized by mean and s.d. computed from the training split. To get optimal validation prediction accuracies, a grid search on hyperparameters, including maximum tree depth, subsampling of training data and probability threshold, was performed on the validation split. The XGBoost model trained with optimal hyperparameters was then applied to the test split. This procedure was repeated for each training–validation–test split, including cross-validation and leave-one-site-out.

#### TabPFN as baseline approach

TabPFN models were used as baseline deep learning approaches to classify binary targets separately. We therefore fit six TabPFN models on the basis of pretrained TabPFN classifiers for classifying six binary conditions. We performed several additional postprocessing steps before proteomics data were input into deep learning models, which were the same for both TabPFN and ProtAIDe-Dx. First, each participant’s proteomics values were normalized by their averaged protein levels^59^. Second, we fit a 10-nearest neighbor data imputer onto the training split to impute missing protein entries. Third, a Gaussian rank normalizer^60^ was fit on the training split to ensure proteins followed a normal distribution. The optimal validation probability threshold was selected on the basis of best F1 scores on the validation set. The TabPFN model fit with an optimal probability threshold was then applied to the test split. This procedure was repeated for each training–validation–test split, including cross-validation and leave-one-site-out.

#### ProtAIDe-Dx

The ProtAIDe-Dx models were implemented as multiple layer perceptron-based networks. Input proteins were fed into multiple multi-layer neural networks to classify six binary conditions jointly. The key consideration of choosing a multi-task over multi-class approach for ProtAIDe-Dx is that diagnostic labels are often incomplete in GNPC, as the dataset was aggregated from multiple cohorts with varying research objectives. For example, a participant diagnosed with AD may not have been formally assessed for PD, resulting in missing labels for certain conditions. In a multi-class classification framework using one-hot label vectors across six diagnostic categories, many vectors would contain missing values, necessitating either imputation or exclusion of these samples, both of which would usually be suboptimal. By contrast, a multi-task learning framework maximizes sample utilization, as each task is trained independently on subjects with labels available for that specific condition.

The imbalanced distribution of the six conditions may bias the model toward the majority class and lead to poor generalization if the loss function is not carefully designed. The loss function of the proposed ProtAIDe-Dx model is a weighted combination of binary cross-entropy loss *L*_BCE_ and multi-class rank loss^61^ *L*_RL_. Label smoothing mitigates overfitting to potentially noisy clinical annotations by calibrating model confidence, while rank loss enhances robustness to distributional skew by optimizing relative sample rankings rather than absolute probabilities. This combined approach ensures improved generalization across all conditions despite uneven representation.

2 $$\begin{array}{l}{L}_{\mathrm{BCE}}=\\ \,\,\,\,\,\,\,\,\,\,\,\,\,-\frac{1}{N}\mathop{\sum }\limits_{k=1}^{N}\mathop{\sum }\limits_{i=1}^{6}\left[\left({y}_{k,i}\left(1-\alpha \right)+\frac{\alpha }{2}\right)\log {\hat{y}}_{k,i}+\left({(1-y}_{k,i})\left(1-\alpha \right)+\frac{\alpha }{2}\right)\log {(1-\hat{y}}_{k,i})\right],\end{array}\,\,$$

where *N* is the number of participants, *k* is the participant index, *i* is the index of conditions, *y*_*k,i*_ is the true label for participant *k* and condition *i*, *α* is a hyperparameter label smoothing factor to set, and ${\hat{y}}_{k,i}$ is the model-predicted probability for the participant *k* and condition *i*.

The role of rank loss *L*_RL_ is to constrain the rank of predicted probabilities across conditions, enabling better joint learning of information across targets. For any two conditions *i* and *j*, the rank loss ${L}_{\mathrm{RL}}^{i,j}$ is

3 $${L}_{\mathrm{RL}}^{i,j}=\frac{1}{N}\mathop{\sum }\limits_{k=1}^{N}\max \left[0,\left({\hat{y}}_{k,i}-{\hat{y}}_{k,j}\right)\left({y}_{k,j}-{y}_{k,i}\right)+{{\varepsilon }}\right],$$

where *N* is the number of participants, *k* is the participant index, *i* and *j* are indexes of two conditions, $\hat{{y}_{k,i}}$ and $\hat{{y}_{k,j}}$ are predicted participant *k*’s probabilities for conditions *i* and *j*, and *ε* is a hyperparameter set as 0.25.

The rank loss across all conditions is

4 $${L}_{\mathrm{RL}}=\frac{1}{N}\mathop{\sum }\limits_{k=1}^{N}\mathop{\sum }\limits_{i=1}^{6}\mathop{\sum }\limits_{j=i+1}^{6}{L}_{\mathrm{RL}}^{i,j}.$$

Therefore, the overall loss function for the proposed ProtAIDe-Dx model is

5 $$L={L}_{\mathrm{BCE}}+\lambda {L}_{{\mathrm{RL}}},$$

where *λ* is a hyperparameter to control the weight of *L*_RL_.

The probability threshold for each target was determined for the highest validation F1 score. We performed a hyperparameter search on the validation split to get the optimal validation BCA using Optuna^62^ with 50 trials. The optimal validation probability threshold was selected on the basis of the best F1 scores on the validation set. The search range of hyperparameters is presented in Supplementary Table 14, with optimal searched hyperparameters presented in Supplementary Table 15 (model evaluated on BioFINDER-2) and Supplementary Data 13 (all cross-validation and leave-one-site-out models). An illustration of ProtAIDe-Dx architecture is shown in Supplementary Fig. 10.

After model fitting, we estimated the model’s overfitting by enabling dropout at the inference stage. For each held-out test split, we repeated the prediction 100 times via stochastic forward passes to obtain an empirical distribution of model outputs across 100 repeats. We then summarized this distribution as ‘confidence intervals’. We interpreted interval width through the bias–variance tradeoff as a proxy for overfitting. An overfit model may achieve low bias by memorizing the training data but at the cost of high variance because its predictions are sensitive to small fluctuations in the training set. When applied to unseen test data, this instability yields greater dispersion across dropout repeats, producing wider intervals that reflect poor generalization.

We performed several additional postprocessing steps before proteomics data were input into ProtAIDe-Dx models. First, each participant’s proteomics values were normalized by their averaged protein levels^59^. Second, we fit a 10-nearest neighbor data imputer onto the training split to impute missing protein entries. Third, a Gaussian rank normalizer^60^ was fit on the training split to ensure normalized proteomics are following a normal distribution.

#### Proposed ensemble of XGBoost and ProtAIDe-Dx

We conducted an ensemble approach for the developed XGBoost and ProtAIDe-Dx models. The ensemble approach’s output probability for each condition was a weighted sum between XGBoost and ProtAIDe-Dx: $w\times {p}_{\mathrm{ProtAIDe}}+(1-w)\times {p}_{\mathrm{XGBoost}}$. On the validation split, we searched for the optimal weight *w*, ranging from 0 to 1 with the step as 0.01, to get the highest validation balanced accuracy. This procedure was repeated for each training–validation–test split, including cross-validation and leave-one-site-out.

#### Model generalization to new sites by *K*-shot transfer learning

We developed a *K*-shot transfer learning framework^63^ to help the developed model generalize to new tasks or new sites. Taking generalization to new sites as an example, only *K* participants were needed to transfer the ProtAIDe-Dx model to this new site, which makes ProtAIDe-Dx easy to scale to real-world clinical settings given model calibration^64^ only needs *K* participants. In this study, we picked *K* = 100 to balance the available sample size for each condition and generalization performance. First, the ProtAIDe-Dx model was applied to new sites to get proteomic embeddings. Second, a simple logistic regression (LR) model was trained on *K* participants’ proteomics embedding and tested on the remaining participants. The performances were evaluated on the remaining participants. Similarly, the developed ProtAIDe-Dx model could transfer to new neurodegeneration-related tasks by training a simple machine learning model on *K* participants.

The underlying assumption is that ProtAIDe-Dx’s embeddings have captured enough signals to represent neurodegenerative diseases broadly. Therefore, training a simple machine learning model on low-dimensional proteomics embedding with limited subjects will avoid overfitting issues compared with directly training new models on raw proteins.

#### Estimating predictive importance

To estimate the predictive importance of individual proteins, the PermFIT approach^23^ was adopted on each test split. For each input protein, the PermFIT approach randomly permuted this protein across participants and computed the cross-entropy differences compared with unpermutated data. The mean and s.d. of cross-entropy differences for this protein were obtained from 100 permutations, and *P* values were calculated on the basis of the mean and s.d., assuming a normal distribution.

In this study, we measured the predictive importance of proteins in two ways, including fold count and *Z* statistics. Once PermFIT was done on all ten splits, the fold count approach counted the frequency of important proteins across those splits using FDR-corrected *P* values. The *Z* statistics approach computed the *z* scores (mean divided by s.d.) on each test split and then averaged *z* scores across ten splits to get the predictive importance of proteins.

To estimate the importance of individual model embeddings in predicting specific diagnoses, we used the Haufe approach. Haufe and colleagues^56^ proved that covariance is more reliable than weights (betas) to interpret feature importances in linear models. Given that the embeddings were linearly combined to make predictions, we computed the covariance between embeddings and predicted probabilities for each condition to estimate the embeddings’ feature importance.

### Model evaluation on GNPC

In addition to reporting classification accuracy, we performed multiple experiments to validate ProtAIDe-Dx models on GNPC data. First, we correlated predicted AD probability with MMSE scores. Given that each train–validation–test split had different probability thresholds, we first divided AD probability by the corresponding AD probability threshold for normalization. Then, we took the logarithm of the mean AD probabilities of participants with the same MMSE score on each testing split. Finally, we computed the Pearson correlation between MMSE scores and the logarithms of the mean AD probabilities. Similarly, we computed the logarithms of mean AD probabilities by *APOE* *ε2*/*ε4* carrier group on each test split and then averaged them across ten splits.

#### Model generalization to new task with an example of prediction of longitudinal clinical progression

We validated the generalization ability of nonlinear proteomics embeddings to new tasks. More specifically, we took the embeddings from baseline visits and used these to predict whether participants with CDR 0 at baseline visits would progress to CDR 0.5 or 1 in their following visits or not. For 3,942 participants with longitudinal visits, we selected participants with CDR 0 at baseline visits and non-decreasing CDR during the following visits. For example, participants with CDR 0, 1 and 0 in their first, second and third visits would be excluded. In total, we selected 1,445 participants with stable CDR 0 in their following visits and 218 participants with progressive CDR in their following visits. It is noted that the baseline visits of the 1,445 + 218 = 1,663 participants have been used in the tenfold cross-validation procedure for ProtAIDe-Dx development. We trained LR models to predict whether these 1,663 participants would progress to CDR 0.5 or 1 in their following visits or not, following the former tenfold cross-validation procedure. We adopted default hyperparameters for LR models from the sklearn package (v 1.6.1).

#### Dimensionality reduction of predicted probabilities with *t*-SNE

We selected 6,332 participants who were either diagnosed as recruited control (*N* = 1,540), AD (*N* = 1,637), PD (*N* = 2,287), FTD (*N* = 175), ALS (*N* = 435) or stroke/TIA (*N* = 256) to project onto a two-dimensional probability map. We excluded individuals with all negatives or multiple positives for the six targets. It is noted that the predicted probabilities of the 6,332 participants were all from test splits, following the former tenfold cross-validation procedure.

The dimensionality reduction algorithm was chosen as *t*-distributed stochastic neighbor embedding (*t*-SNE) from the OpenTSNE library (v 1.0.2). We used OpenTSNE over sklearn because OpenTSNE supports inference on new data with fit *t*-SNE models, allowing us to investigate new participants such as MCI–SCI patients on the same *t*-SNE map. The parameters of *t*-SNE were default values except for setting perplexity to 1,000 to better capture global structures.

#### Assessing disease heterogeneity

For each target, *K-*means clustering was performed on the two-dimensional *t*-SNE probability maps of participants within each diagnostic category. To pick the optimal number of clusters, we looped it over from 2 to 10 to pick the one with the highest Silhouette score. In summary, we obtained two control clusters, three AD clusters, four PD clusters, two FTD clusters, two ALS clusters and five stroke/TIA clusters.

To figure out the differentially expressed proteins across clusters, we ran GLMs across 7k proteins on positive participants for each diagnostic category. Therefore, for each diagnostic category, we have GLMs following the formula

6 $$\mathrm{Protein} \sim {C}_{1}+\ldots +{C}_{K}+\mathrm{Age}+\mathrm{Sex}+\mathrm{Site}+\mathrm{AverageProteinLevel}.$$

${C}_{1}\ldots {C}_{K}$ are the binary variables indicating which cluster the participants were from. Site is the categorical variable that indicates which site the participants were from.

#### Drug-related protein mapping

To systematically evaluate potential medication confounding across the 75 discriminative proteins, we performed a targeted protein–drug lookup in DrugBank^65^ by querying each PermFIT-selected protein using its UniProt identifier. We then filtered and annotated drugs for relevance to neurodegenerative or vascular indications using a rule-based approach that integrated Anatomical Therapeutic Chemical (ATC) codes, DrugBank ‘drug category’ fields and free-text drug descriptions. In brief, ATC codes were tokenized (allowing multiple codes per drug) and matched using either prefix-based rules (for drug classes) or exact-code rules (for specific agents), whereas ‘drug category’ entries and descriptions were screened using curated keyword lists and case-insensitive regular expressions.

Drugs were labeled as AD therapies if they carried ATC codes starting with N06DA or N06DX and/or if DrugBank category/description text contained AD-related terms, including ‘anti-dementia’, ‘Alzheimer’, ‘cholinesterase inhibitor(s)’ or ‘NMDA receptor antagonist(s)’, as well as description mentions of ‘Alzheimer’ or ‘dementia’. Drugs were labeled as PD therapies if they carried ATC codes starting with N04 and/or if category or description text contained PD-related terms including ‘Parkinson’, ‘dopaminergic’ or ‘monoamine oxidase’, as well as description mentions of ‘Parkinson’. ALS drugs were labeled using an exact ATC match to N07XX02 (riluzole) and/or ALS-related description terms including ‘amyotrophic’, ‘ALS’ or ‘motor neuron disease’. Stroke/TIA-related drugs were labeled if they carried ATC codes starting with B01 (antithrombotic agents) and/or if the category or description text contained cerebrovascular terms, including ‘stroke’, ‘TIA’, ‘cerebrovascular’, ‘antiplatelet’, ‘anticoagulant’, ‘thrombolytic’ or ‘plasminogen activator’ or description mentions of ‘transient ischemic’. In addition, we flagged cardiovascular risk factor medications using ATC prefixes C02/C03/C07/C08/C09/C10 (antihypertensives/diuretics/beta blockers/calcium-channel blockers/renin–angiotensin system agents/lipid-modifying agents) and grouped these with stroke/TIA-related drugs.

#### Assessing site-to-site generalization variability

We conducted a site-to-site generalization experiment in which models were trained exclusively on data from a single site (development site) and evaluated on all remaining sites (test sites). As expected, classification performance was higher on the development site than on the test sites. We selected TabPFN as the predictive model because it does not require hyperparameter tuning. The same sites used in the leave-one-site-out experiments were included here. For each development site, the data were randomly split into two equal halves. Two TabPFN models were trained independently on each half and evaluated on the other half, with development-site accuracy defined as the mean of these two within-site accuracies. The two trained TabPFN models were then applied to the remaining external test sites, and test-site accuracy was defined as the mean performance of both models on the test sites. To quantify site-to-site variability, we defined relative performance as the ratio of test-site accuracy to development-site accuracy, with lower values indicating stronger site effects.

### Enrichment analysis

To evaluate the cell-type expression of genes of interest, we utilized two independent single-cell RNA sequencing (scRNA-seq) datasets. The first dataset comprised single-cell transcriptomes from 2.3 million cells obtained from the aged human prefrontal cortex of 427 participants in the Religious Order Study (ROS) and the Rush Memory and Aging Project (MAP)^66^. This dataset includes neuronal, glial and vascular cell types. The second dataset, the Human Brain Vascular Atlas, profiled mainly vascular and perivascular cell types, using 143,793 single-cell transcriptomes from the hippocampus and cortex of eight postmortem samples^67^.

For the two datasets, we downloaded the Seurat objects and used the R package Seurat v4.3.0^68^, applying the AverageExpression function to compute cell-type expression levels. We then calculated the proportion of gene expression across all major neuronal and non-neuronal populations.

For organ enrichment analysis, we used organ-enriched genes as defined in previous studies^69^ on the basis of the Gene Tissue Expression Atlas (GTEx) bulk RNA-seq database. A gene was classified as organ-enriched if its expression was at least fourfold higher in a specific organ or tissue compared with any other, following the Human Protein Atlas definition^70^. We considered organ categories including adipose tissue, artery, brain, esophagus, heart, immune, intestine, kidney, liver, lung, muscle, pancreas, skin, stomach and whole blood. Cell- and organ-enriched genes were then mapped to proteins quantified in the SomaScan assay.

To determine whether a specific set of proteins exhibited preferential expression in certain cell types, we calculated the average expression level of the significant protein list across each cell type. To determine whether this enrichment exceeded random expectation, we generated 10,000 randomly sampled gene lists from the Somalogic background set, each maintaining the same protein count as the significant list. A probability distribution was then constructed on the basis of the average expression levels of these random lists across cell types. This allowed us to quantify the likelihood that the observed enrichment was greater than expected by chance, while accounting for background expression variability. Cell types with an FDR-corrected *P* value <0.05 (Benjamini–Hochberg correction) were considered significantly enriched.

We performed overrepresentation analyses of Kyoto Encyclopedia of Genes and Genomes (KEGG) pathways and Gene Ontology (GO) Biological Process (BP) terms using enrichKEGG() and enrichGO() functions in clusterProfiler (v4.14.4) and org.Hs.eg.db (v3.20.0) in R (v4.4.2). The input gene lists consisted of proteins that were significantly up-regulated, down-regulated or both in disease clusters defined by logFoldChange and an FDR-corrected *P* value <0.05 (Benjamini–Hochberg correction). We used all human proteins as the background set for overrepresentation analysis. We reported only those KEGG pathways and GO BP terms that met an FDR threshold of <0.05. To reduce redundancy among significantly enriched BP terms, we applied the clusterProfiler simplify() function using Wang’s semantic similarity measure with a cutoff of 0.7.

Enrichment analyses were performed on proteins differentially expressed across *t*-SNE clusters. Enrichment analysis was additionally performed on embedding-specific proteins. To annotate the biological process of each nonlinear proteomics embedding, we found ‘embedding specific’ proteins using regression. For each embedding, we took the 648 proteins from the cross-validation feature selection to train an XGBoost regression model, with grid search on validation split for the lowest mean squared errors. It is noted that the model was trained to generalize to BioFINDER-2 has 32 embedding dimensions. Once these models were trained, we thresholded proteins with ‘total_gain‘ over 50 as ‘embedding specific’ proteins for enrichment analysis.

### Model evaluation on BioFINDER-2

In the BioFINDER-2 dataset, where we correlated each biomarker with the different predicted probabilities separately in each of the six diagnostic groups (CU, SCD, MCI, AD, parkinsonism and other), FDR correction was applied across all comparisons performed. We also calculated the Pearson correlation between embeddings and biomarkers for BioFINDER-2 participants who had corresponding biomarkers available. FDR correction was applied across all comparisons performed. The model applied to the BioFINDER-2 dataset was the leave-one-site-out model trained on the remaining GNPC sites, with BioFINDER-2 entirely excluded. BioFINDER-2 was fully held out from all stages of model development, including feature selection, hyperparameter tuning and model fitting, and was used only for inference and downstream evaluation/clinical utility analyses. Therefore, there was no sample overlap or information leakage between training and testing datasets.

#### Differential diagnostics of multiple neurodegenerative diseases

We further investigated whether proteomics embeddings would provide unique or additive value in distinguishing multiple neurodegenerative diseases in the BioFINDER-2 dataset. We selected 231 patients with AD (with abnormal CSF Aβ42/Aβ40 ratio), 111 patients with PD (with positive CSF α-synuclein status), 39 patients with FTD and 20 patients with stroke (with infarcts), excluding patients with multiple neurodegenerative diseases. We performed a fivefold cross-validation, ensuring the diagnosis distribution was balanced across folds. Three out of five folds were used to train an Support Vector Machine model, one fold was used to tune hyperparameter *C* from 0.001 to 100 to get an optimal balanced accuracy score and the remaining one was used for the test. This procedure was repeated five times to ensure each fold was used as a test fold.

Four sets of features were selected to validate the effectiveness of our proteomics embeddings. Model 0 only used age and sex; model 1 used age, sex and the top 5 principal components of proteomic embeddings; model 2 used age and sex plus clinical biomarkers, including MMSE, plasma p-tau217, plasma NEFL and mean cortical thickness of AD-signature meta-ROI; and model 3 used all features in model 0, model 1 and model 2. It is noted that age, MMSE, mean cortical thickness of AD-signature meta-ROI, plasma p-tau217 and plasma NEFL were *z*-normalized. Both principle component analysis and *z*-normalization were fit on training splits. Once models were trained, we concatenated participants from five test folds to perform bootstraps 1,000 times to perform statistical tests.

#### Mixed-effect modeling of longitudinal cognitive decline

To assess whether baseline characteristics (for example, clinical diagnoses and model predictions) could differentiate longitudinal trajectories of decline, we fit linear mixed-effects models with individual variability modeled as random effects. Cognitive performance was measured using the MMSE.

First, we evaluated whether baseline clinical diagnoses could differentiate longitudinal trajectories of decline.

7 $${\mathrm{MMSE}} \sim {\mathrm{Age}}+{\mathrm{Sex}}+{\mathrm{Site}}+{\mathrm{BaselineDx}}\times {\mathrm{Year}}+{\mathrm{Year}}|{\mathrm{SubjectID}}.$$

BaselineDx is the categorical variable that indicates baseline diagnoses and $\mathrm{Year}|\mathrm{SubjectID}$ specifies random effects of individuals over time (in units of years).

Then, we evaluated whether baseline predictions could differentiate longitudinal trajectories of decline; we included baseline diagnoses as covariates.

8 $$\begin{array}{l}{\mathrm{MMSE}} \sim {\mathrm{Age}}+{\mathrm{Sex}}+{\mathrm{Site}}+{\mathrm{BaselineDx}}\\ +{\mathrm{BaselinePrediction}}\times {\mathrm{Year}}+{\mathrm{Year}}|{\mathrm{SubjectID}}.\end{array}\,\,$$

BaselinePrediction is the categorical variable that indicates baseline predictions and $\mathrm{Year}|\mathrm{SubjectID}$ specifies random effects of individuals over time (in units of years).

#### A two-cutoff approach of predicted probabilities

In the external BioFINDER-2 dataset, we applied a two-cutoff strategy on predicted probabilities to determine biomarker positivity. Participants were divided into non-SCD and SCD groups, with the non-SCD group used to derive the cutoffs and the SCD group serving as the test set. Cutoffs were chosen to yield 90% NPVs and PPVs, except for CSF α-synuclein, where the PPV was set at 40% owing to sample validity constraints.

### Individual disease risk report

To assess contributing proteins for individual prediction, Shapley Additive Explanations (SHAP) values by SHAP package (v 0.48.0) were computed for each of six conditions, with training participants as the background set. To associate the top contributing proteins with health-related traits, we applied a proteome–phenome atlas^42^ to find the top correlated traits for each protein. The proteome–phenome atlas was computed on the basis of Olink proteomic data. Therefore, there might be no matched results for some of the contributing proteins; we only included available protein–trait associations.

### Statistical analysis

We applied a corrected resample *t*-test to compare model performances in cross-validation and leave-one-site-out experiments. To examine whether the distribution of variables varies across *t*-SNE clusters or not, we conducted two-proportion *z*-tests for binary variables with two clusters, chi-squared tests of independence for both binary variables with three or more groups and multi-category variables, *t*-tests for continuous variables with two clusters and one-way analysis of variance for continuous variables with three or more groups. To compare model performances in comorbidity detection, we applied *t*-tests on 1,000 bootstrapped accuracies. We conducted *t*-tests for comparing probability distributions across biomarker status. It is noted that all *P* values were FDR corrected with *α* as 0.05 using the Benjamini–Hochberg procedure.

### Reporting summary

Further information on research design is available in the Nature Portfolio Reporting Summary linked to this article.

## Online content

Any methods, additional references, Nature Portfolio reporting summaries, source data, extended data, supplementary information, acknowledgements, peer review information; details of author contributions and competing interests; and statements of data and code availability are available at 10.1038/s41591-026-04303-y.

## Supplementary information

Supplementary Information Supplementary Results 1–4, Figs. 1–11, Tables 1–15, References and GNPC V1 Full Member List and Affiliations.

Reporting Summary

Supplementary Data 1–13 Supplementary Data 1: Cross-validation performances and *P* values. Supplementary Data 2: Variable distribution by tSNE cluster. Supplementary Data 3: Differential abundance analysis by tSNE cluster with BP. Supplementary Data 4: Differential abundance analysis by tSNE cluster with KEGG. Supplementary Data 5: Medication–protein associations. Supplementary Data 6: Enrichment analysis by embedding with BP. Supplementary Data 7: Enrichment analysis by embedding with KEGG. Supplementary Data 8: Correlations between embeddings and biomarkers. Supplementart Data 9: Cell type enrichment analysis by embedding with BBB atlas. Supplementary Data 10: Cell-type enrichment analysis by embedding with ROSMAP atlas. Supplementary Data 11: Leave-one-site-out performances and *P* values. Supplementary Data 12: Correlations between predicted probabilities and biomarkers. Supplementary Data 13: Optimal hyperparameters of ProtAIDe-Dx model.

## Data Availability

GNPC (https://www.neuroproteome.org/) is open access. Pseudoanymized BioFINDER-2 data will be shared by request from a qualified academic investigator for the sole purpose of replicating procedures and results presented in this article and as long as data transfer is in agreement with European Union legislation on the general data protection regulation and decisions by the Swedish Ethical Review Authority and Region Skåne, which should be regulated in a material transfer agreement.

## Code availability

Code is publicly available via GitHub at https://github.com/DeMONLab-BioFINDER/An_ProtAIDe-Dx. Y. Xiao reviewed the code before merging it into the GitHub repository to reduce the chance of coding errors.
