# Supplementary material for: A deep joint-learning proteomics model for diagnosis of six conditions associated with dementia
Source: Nat Med. 2026 Mar 31;32(5):1852–64. doi: 10.1038/s41591-026-04303-y (PMC13190262; doi:10.1038/s41591-026-04303-y)
Supplement: Supplementary file 1 — Supplementary Results 1–4, Figs. 1–11, Tables 1–15, References and GNPC V1 Full Member List and Affiliations. [file 41591_2026_4303_MOESM1_ESM.pdf]

nature medicine

Supplementary information https://doi.org/10.1038/s41591-026-04303-y

A deep joint-learning proteomics model for diagnosis of six conditions associated with dementia

In the format provided by the authors and unedited

Supplementary Results

S1 Diagnostic probabilities reveal disease heterogeneity and co-pathology.

The second (i.e. first non-dominant) cluster of healthy controls emerged at the intersection of shallow extremes of the AD and stroke/TIA regions. This group was older than the dominant cognitively unimpaired group (71.7±11.5 versus 57.7±15.8, FDR-corrected p=4e-56), worse educated, and had higher rates of hyperlipidemia (Fig. 2G, 28% versus 15%, FDR-corrected p=2e-6), other cardiovascular risk factors, more frequent history of smoking, higher cancer and diabetes rates and worse cognition (SuppData 2). Differential abundance analyses comparing the two control clusters revealed downregulation of proteins relating to inflammation in the dominant cluster compared to the minor cluster (SuppData 3, SuppData 4). These two clusters may resemble healthy (dominant) vs. unhealthy (minor) aging.

Two minor AD clusters also emerged, with one colocalizing in the stroke/TIA region, and the other in the PD region. Patients in these minor clusters showed lower rates of *APOE* ε4 carriage (FDR-corrected p=2e-7) and slightly better cognition (FDR-corrected p=3e-10). Patients in the minor cluster in the stroke/TIA region showed increased frequency of hypertension (Fig. 2H, 50% versus 26% and 35%, FDR-corrected p=0.01) and other vascular risk factors (SuppData 2), likely signaling mixed AD and vascular co-pathology, or misdiagnosis. Patients in the minor cluster within the PD region were younger and were predominantly male (69% vs. 43% and 46%, SuppData 2, FDR-corrected p=0.03). Compared to the other clusters the dominant AD cluster had a higher abundance of proteins involved in cell death, damage response and mitochondrial activity, but lower abundance of proteins involved in immune and defense response. The minor cluster in the stroke/TIA region meanwhile had more abundance of proteins involved in immune response, plasticity and AD-related signals (i.e. APP and reelin processes) (SuppData 3, SuppData 4). Interestingly, both the minor clusters showed decreased abundance of proteins associated with energetic metabolisms, and in the case of the minor cluster in the PD region, specifically glucose metabolism (Fig. S3A, SuppData 3, SuppData 4). This is interesting given the robust finding of hypometabolism (especially relative to brain atrophy) in PD[1,2], compared to AD, which can also produce phases of cortical hyperactivity[3].

Perhaps most interestingly, a minor cluster for ALS emerged distant from the dominant cluster but in closer proximity to the FTD region. This minor cluster showed a higher rate of C9orf72 mutations (Fig. 2I, 50% versus 18%, FDR-corrected p=0.03), marginally higher rates of SOD1 mutations (Fig. S4, 50% versus 18.8%, unadjusted p=0.32), and a higher rate of mild cognitive impairment (SuppData 2, 99% versus 67%, FDR-corrected p=7e-11). Differential abundance between these two groups revealed upregulation of proteins relating to cell death in the C9orf72 cluster, and downregulation of proteins relating to metabolism and immunity (Fig. S3B, S3C; SuppData 3, SuppData 4). The dominant cluster meanwhile showed downregulation of proteins relating to muscle activity, tissue remodeling and neuroplasticity. Differential abundance and characteristics for PD, stroke/TIA and FTD can be found in SuppData 3, and SuppData 4.

S2 Model interpretation highlights disease-specific networks and key discriminative proteins.

For PD, we replicated previous recent work[4] highlighting SUMF1 as discriminative of PD. The loss of SUMF1, which encodes an essential enzyme for activating cellular sulfatases [5], leads to lysosomal dysfunction and blocked autophagy [6]. Therefore, SUMF1 may play a role in impaired α-synuclein clearance, given that lysosomal dysfunction contributes to the spread of α-synuclein [7].

SERPINF2 has also been reported as associated with PD by previous work[8]. Similarly, PRL and C3 have long been associated with PD. PRL demonstrates neuroprotective effects through activation of JAK/STAT and PI3K/Akt pathways and reduction of excitotoxicity in preclinical studies [9] and it has been reported to link with dopaminergic function[10–12]. C3 has been indicative of immune changes detectable even before symptom onset[13–16]. Other interesting PD-discriminative proteins emerged. GPT has previously been shown to be discriminative in different synculeinopathies[17], but related protein GPT2 has been implicated in midbrain neurodegeneration[18]. B4GALT-family proteins have been implicated in midbrain responses to environmental exposure[19], while HERC1 is an endosomal system protein that has been previously linked to PD[20,21]. CSF NPTXR has emerged as a potential biomarker for synaptic dysfunction [22–24] and Lewy body disorders [25,26], and plasma levels were predictive of PD in our dataset. NPTXR regulates synaptic plasticity and excitatory synapse formation, and recent studies have shown it to have a neuroprotective role against PD pathology [27,28].

A set of proteins was associated with both healthy controls and ALS, perhaps reflecting the model keying into the younger age or less prevalent cognitive impairment in these populations. For example, CNTFR is an astrocytic neurotrophic factor receptor that has been associated with better cognition and slower cognitive decline, in both CSF[29] and plasma[30], while TNNT2 is a dementia-associated heart-health protein[31–33], which has been recently linked to ALS[34]. CNTFR, in particular, has a long history of interest as an ALS treatment due to its importance in the survival of motor neurons[35,36]. Meanwhile, POMGNT1 is likely more ALS-related, given its well documented association with congenital muscular dystrophy[37,38], and LRTM1 plays a role in both brain and skeletomuscular systems[39]. KCNIP3 showed the strongest discriminative signal for ALS [34,40,41], it has been listed among ~300 ALS-associated genes[42] and reported to be upregulated in ALS patients[43,44], with similar observations in animal models[45]. In plasma proteomics, KCNIP3 levels were found to be increased by approximately 60% in ALS patients receiving riluzole treatment[34]. Functionally, KCNIP3 binds voltage-gated potassium channels and regulates A-type currents, which counteract depolarizing inputs and modulate neuronal excitability[46]. These findings suggest that elevated KCNIP3 levels in ALS patients may indicate stronger inhibitory control, suggesting a compensatory reduction in neuronal excitability. Certain proteins were discriminative of both ALS and FTD including HEY1, involved in Notch signaling and upregulated in mutant C9orf72 organoids[47].

However, several FTD-discriminating proteins captured our interest. NEFL, involved in neuronal damage, has been commonly found to have increased concentrations and in multiple neurodegenerative diseases [48–50], including FTD [51,52], making it unsurprising as a protein predictive of FTD in the present study. Interestingly, PI3 was found to be discriminative for FTD. PI3 has previously been reported to be abundant in the plasma of cognitive impairment and dementia groups[53] and predictive for future dementia risk[54]. Given that PI3 encodes elafin, a serine protease inhibitor with potent anti-inflammatory activity [55], and that all major genetic forms of FTD exhibit elevated inflammatory signatures[56,57], PI3 may influence FTD pathology by modulating neuroinflammatory responses, either as a compensatory protective factor or as a biomarker of inflammatory dysregulation, a possibility that warrants further experimental validation. SERPINA3 levels are altered in tissue of C9orf72 patients[58], CSF levels differentiated symptomatic from unaffected FTD mutation-carrying patients[59], and SERPINA3 levels were returned to normal after GRN-mutation mouse models were exposed to peripheral brain-penetrant GRN[60]. IGF2R has anti-inflammatory properties and is reduced across many neurodegenerative disorders, including in tissue of FTD patients and in GRN-insufficient cell models[61–64]. MAEA has been shown to interact with phosphorylated tau[65] and TDP-43[66], while CSF STC1 levels differ across neurodegenerative disorders[67].

Looking at proteins discriminative of TIA/stroke, interestingly, several were also discriminative for AD (NPTXR, CD93) or PD (SUMF1, C3). While this may indicate that these proteins are useful for differentiating these diseases one from another, previous research instead suggests they may represent overlapping biological processes across disorders: neuronal pentraxin is a general signal of synaptic health[24], while CD93 is likely involved in blood brain barrier integrity[68,69], is altered in entorhinal endothelial cells in AD[70], has been implicated in neurovascular disease[71,72], and is generally a predictive marker of cognition[24]. C3 has also been reported as a meaningful marker in stroke patients[73–76]. Meanwhile, DCP1B has also been associated with neurovascular disease phenotypes[77] and is discriminative for TIA/stroke in this study, but has previously been more associated with PD[78,79], again possibly suggesting some shared pathways or etiologies among these disorders. Among those proteins most discriminative for TIA/stroke, perhaps the most promising being RAN. RAN, traditionally recognized for its roles in cell division and signaling [80,81], has recently emerged as a modulator of oxidative stress, apoptosis, and glial activation in models of ischemic brain injury[82]. By conferring neuroprotection and attenuating neuroinflammation, it represents a promising therapeutic target for stroke and related neurovascular disorders, further supported by genetic evidence linking an RNA variant (rs1435) to post-stroke mortality in patients with large-artery disease[83]. Another interesting protein predictive of stroke/TIA was MATAP2. MATAP2, best known for its role in angiogenesis [84], has also been implicated in stroke. Experimental ischemia shows calpain-mediated cleavage of MATAP2, linking it to stress pathways during neuronal injury[85], while its functions in endothelial remodeling[86] and glial inflammation[87] suggest it may influence both acute injury and recovery. Together, these findings highlight MetAP2 as a potential but underexplored therapeutic target in neurovascular disease.

Perhaps the most interesting proteins are those discriminative of healthy controls compared to other diseases, due to their potential as markers for general brain health. Many of the proteins associated with healthy controls seemed to be involved with discriminating them from other diseases, such as AD (e.g., ACHE, NPTXR, SMOC1, SPC25) or PD (e.g., SUMF1). However, some novel and interesting proteins were also found to be highly discriminative of certain conditions. For instance, GLO1 helps to manage mitochondrial and synaptic stress driven by advanced glycation end products, and has been shown to improve neurovascular coupling and bring about cognitive benefits in mice[88–90]. TGFB1 is critical to the transition between homeostatic and disease-responsive microglial states[91–93], and while its levels in the brain have been associated with negative cognitive and neurodegenerative outcomes[94,95], it may play an overall protective role[96]. VAT1[97,98] and STX1A[99,100] are both vesicular proteins that have been previously implicated in cognitive reserve, where the latter seems to be crucial for clearance of cellular debris via lysosomal exocytosis[101]. Both PDE11A[102–106] and IGF2[64,107–109] are expressed in limbic brain regions, play a role in memory processes, and have consistently been shown to rescue cognition in aging and neurodegeneration mouse models. PDE11A is a cAMP/cGMP-degrading enzyme that accumulates ectopically in the ventral hippocampus of both mice and humans with age[105,106]. IGF2, meanwhile, is neuronally enriched, involved in endosomal/lysosomal processes, is decreased in the hippocampus of AD patients and has also been shown to be neuroprotective in a PD context and various other contexts that involve abnormal protein accumulation[62,64,108]. Given our interest in proteins discriminative of healthy cognition vs. all diseases, we ran a new model where SCD/MCI cases were now included as negative cases for all classes. This forced the model to distinguish cognitively healthy individuals additionally from mild (or in most cases, no objective) cognitive impairment or uncertain etiology, increasing specificity for cognitive health. Unsurprisingly, the model performed worse all around, particularly for classifying recruited

controls (77% vs 83%). However, the model interestingly identified OMG (oligodendrocyte myelin glycoprotein)[110] as highly discriminative of cognitively unimpaired controls compared to cognitively impaired patients across all ten folds (Extended Data Fig. 3).

We examine this underlying biology by observing the contribution of embeddings to diagnostic prediction, isolation and enrichment analysis of proteins involved in each embedding (Fig. 3C, SuppData 6, SuppData 7), and association between the embeddings and disease-specific biomarkers in an external dataset (Fig. 4C; see below). We employed a novel method for discovering the selected 648 proteins involved in each embedding (see Methods), though the approach does not indicate directionality of the effects. Contributing protein sets therefore represent a combination of co-abundant proteins and proteins with anti-correlated abundance within the embedding.

Given that proteins in blood come from organs throughout the body, we expected these embeddings to represent processes stemming from multiple organs. However, brain-specific proteins were highly prevalent across all embeddings (Fig. 3B). We therefore tested for enrichment of specific neural cell types (SuppData 9, SuppData 10). Embedding Z2 showed strong specificity to the brain, and to neurons in particular (SuppData 9). Z2 was also discriminative of cognitively unimpaired, AD, and stroke/TIA diagnoses (Fig. 3C), and enrichment terms relating to neuronal resilience, synaptic communication, and molecular homeostasis (SuppData 6), and was associated with older age, worse cognition and unhealthier levels across multiple markers in an external sample (Fig. 4C). This embedding may represent neuronal functional decline, reflecting reduced resilience and synaptic dysregulation that contribute to cognitive impairment across aging and neurodegeneration. Embedding Z23 differentiated controls from neurodegenerative conditions and was enriched for proteins expressed by oligodendrocyte precursor cells (OPC, SuppData 9, SuppData 10). The associated enrichment terms included immune activation, structural remodeling, cytoskeletal organization, proteostasis, vascular function, and developmental processes (SuppData 6). This embedding was also associated with older age, adverse biomarker profiles, and male sex. Together, these features suggest that Z23 may capture glial vulnerability pathways that link aging and sex to increased neurodegenerative disease risk.

Embedding Z3 showed specificity for AD (Fig. 3C) and was associated with worse cognition and AD biomarkers in an external sample (Fig. 4C). The enrichment in sex-related reproductive and hormonal regulation suggests that part of the signal may reflect female sex differences in AD risk, a rapidly expanding line of research[111]. In addition, enrichment for OPC proteins, synaptic activity and remodeling, phosphorylation, and tissue damage (Fig. 3C, SuppData 9, SuppData 10) may reflect processes linking sex-related pathology with cellular vulnerability, synaptic dysfunction, and injury in AD. Embedding Z21 was discriminative for both ALS and PD diagnosis and was associated with younger age, better cognition, and healthier levels across multiple markers in an external sample (Fig. 4C). Z21 showed specificity to both brain and muscle systems (Fig. 3B) and was enriched for proteins involved in muscle energy buffering, contractile apparatus integrity, and structural remodeling, which are critical for muscle performance and therefore highly relevant to motor and movement disorders. Embedding Z25 was discriminative for both AD and PD diagnosis and represented the most important embedding for stroke/TIA classification (Fig. 3C). In an external sample, Z25 was associated with older age, poorer cognition, and biomarkers of vascular pathology, neurodegeneration, and inflammation (Fig. 4C). Functional enrichment analysis indicated that Z25 was linked to proteins involved in vasculature development, angiogenesis, blood circulation, and the regulation of vessel diameter and permeability, suggesting a signal of vascular activity and potential blood–brain barrier permeability. Finally, some embeddings appeared to capture proteomic features associated with brain

health or resilience (Fig. 3C, SuppData 6, SuppData 7). For example, embedding Z1 discriminated cognitively unimpaired individuals from patients (Fig. 3C). In an external cohort, it was associated with tau pathology, but otherwise with better cognitive performance, and generally healthier biomarker profiles (Fig. 4C). The enriched terms of Z1 underscore processes related to responses to damage and hypoxia, protein response to the nucleus, vascular and metabolic homeostasis, and negative regulation of inflammatory and complement systems. Together, these findings suggest that embedding Z1 may capture protective or resilience signals, or may be capturing a very early disease response. A more comprehensive view of shared GO terms between embeddings and biological similarities of embeddings confirmed by biomarkers could be found in Fig. S5.

S3 Proteomics provide additive information to diagnosis in a memory clinic sample.

To test the limits of ProtAIDe-Dx's capability for extension to novel differential diagnosis tasks, we tested whether ProtAIDe-Dx embeddings could distinguish PD (n=78), DLB (n=48), PSP (n=34), and MSA (n=16) in the BioFINDER-2 cohort. This task resulted in an accuracy of 0.52 ± 0.04, which was significantly greater than the null accuracy of 0.44 ($p = 0.012$), notable given that ProtAIDe-Dx was not trained for this specific task. While these results suggest that the embeddings may capture information relevant to PD subtypes, the MSA subgroup is small (n=16), which may limit the robustness of our estimates. Findings should therefore be interpreted cautiously and further examined in larger cohorts.

S4 Proof-of-concept diagnostic report by ProtAIDe-Dx.

Case B (Extended Data Fig.8) presented with MCI in their late-60s, and was given a probable diagnosis of AD by ProtAIDe-Dx, likely due to proteins indicating AD-related Aβ42 (PLCB1), and neuroinflammation (CFD). CSF and PET biomarkers confirmed the presence of AD Aβ and tau pathology in this patient. Finally, Case C was a late-70s male without any subjective or objective cognitive impairment, recruited from the Malmö population. ProtAIDe-Dx predicted a diagnosis of cognitively unimpaired with underlying neurovascular disease (Extended Data Fig.9). CSF and PET biomarkers were negative for AD pathology, but the MRI showed enlarged lateral ventricles, periventricular WMH burden and widespread cortical atrophy indicative of neurovascular disease.

Supplementary Figures

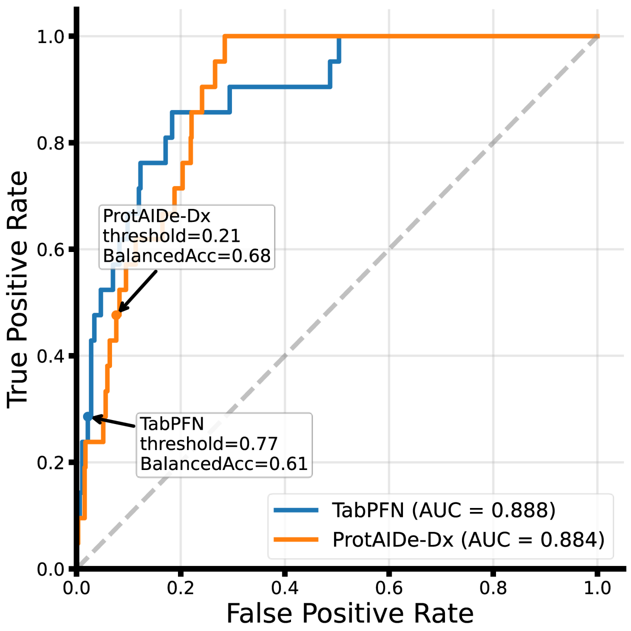

Figure S1. AUROC can be misleading under extreme class imbalance. ROC curves for ProtAIDe-Dx and TabPFN in one test fold's FTD prediction (1% positive) show similar AUROC, whereas ProtAIDe-Dx yields substantially higher balanced accuracy. Decision thresholds were selected by maximizing F1 on the validation set using the same procedure for both models.

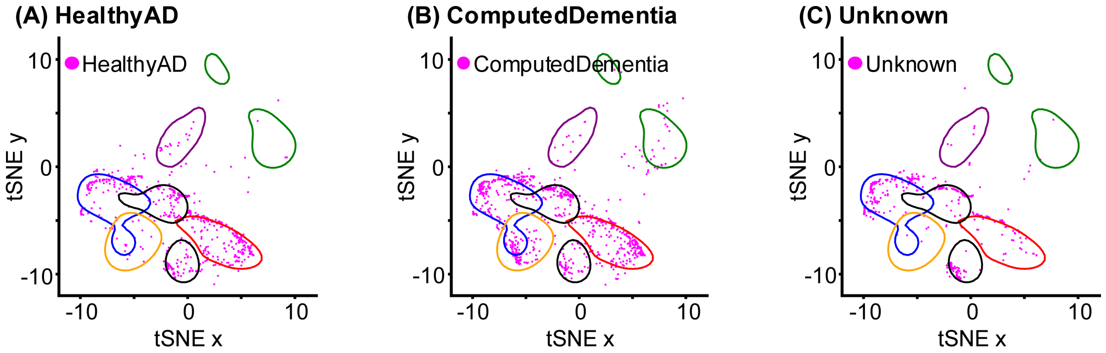

Figure S2. Projection of held-out test participants onto the fitted 2-dimensional t-SNE diagnostic probability map. Test participants were excluded from model training and development. (A) HealthyAD group (n=662), participants clinically diagnosed with Alzheimer's disease but with preserved cognition (MMSE ≥ 26); (B) ComputedDementia group (n=1,062), participants without an available clinical diagnosis but with impaired cognition (MMSE < 19 or CDR ≥ 1); (C) Unknown group (n=542), participants with no available clinical diagnosis or cognitive scores (MMSE/CDR).

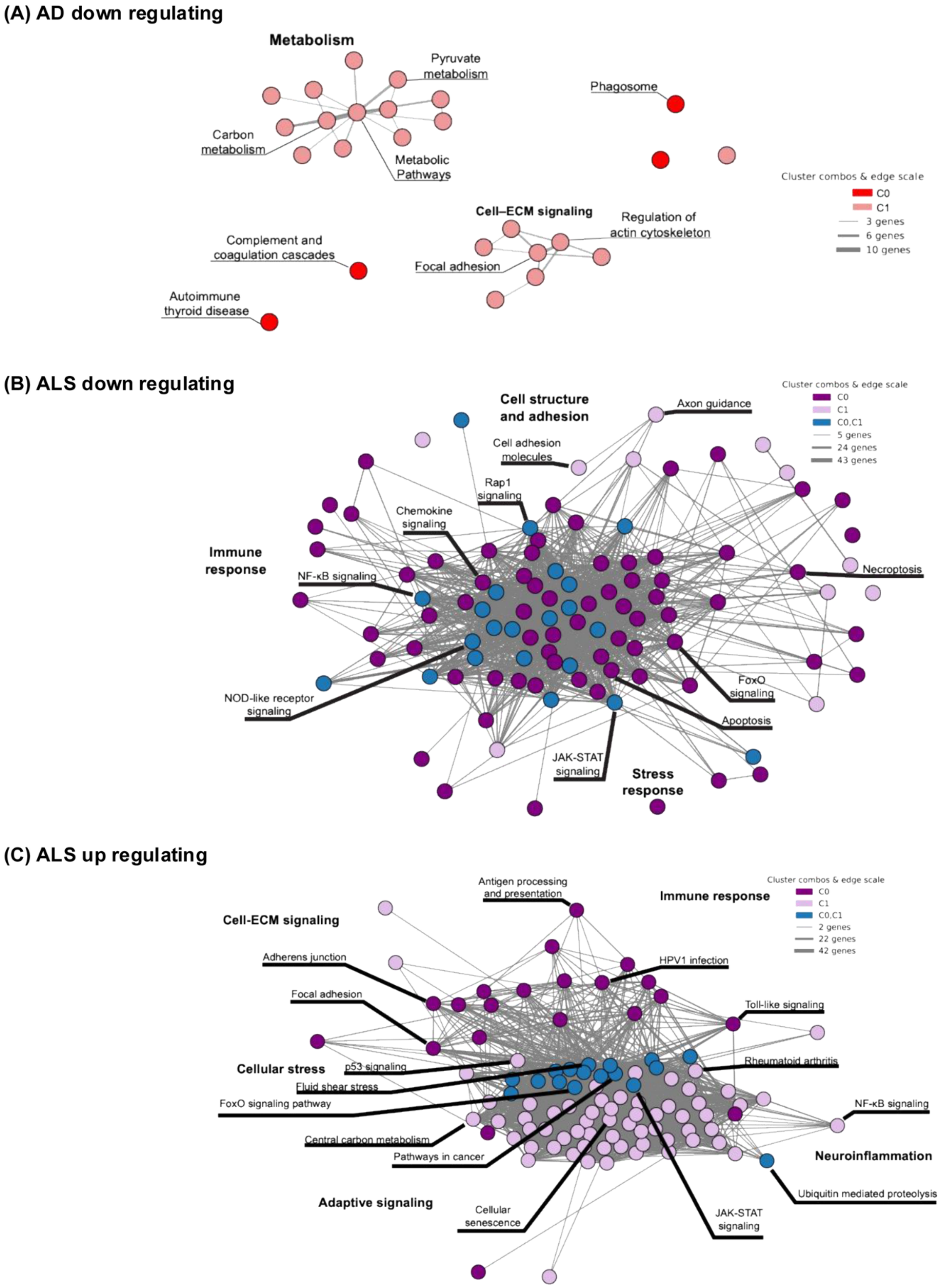

Figure S3. KEGG terms (pFDR < 0.05) enriched among up- or down-regulated proteins in AD and ALS clusters. Node shape denotes KEGG community; color indicates cluster. (A) KEGG terms enriched among down-regulated proteins across three AD clusters; (B) KEGG terms enriched among down-regulated proteins across two ALS clusters; (C) KEGG terms enriched among up-regulated proteins across two ALS clusters.

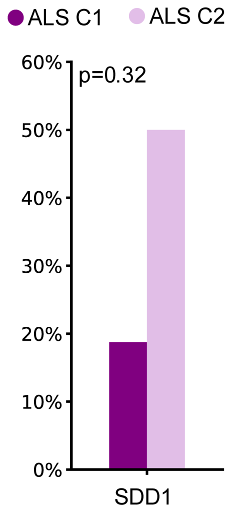

Figure S4. The distribution of SOD1 mutations varied across the ALS clusters. Among patients with available SOD1 information, 3/16 (18.8%) in ALS cluster C1 and 1/2 (50.0%) in ALS cluster C2 harbored SOD1 mutations. P value was unadjusted.

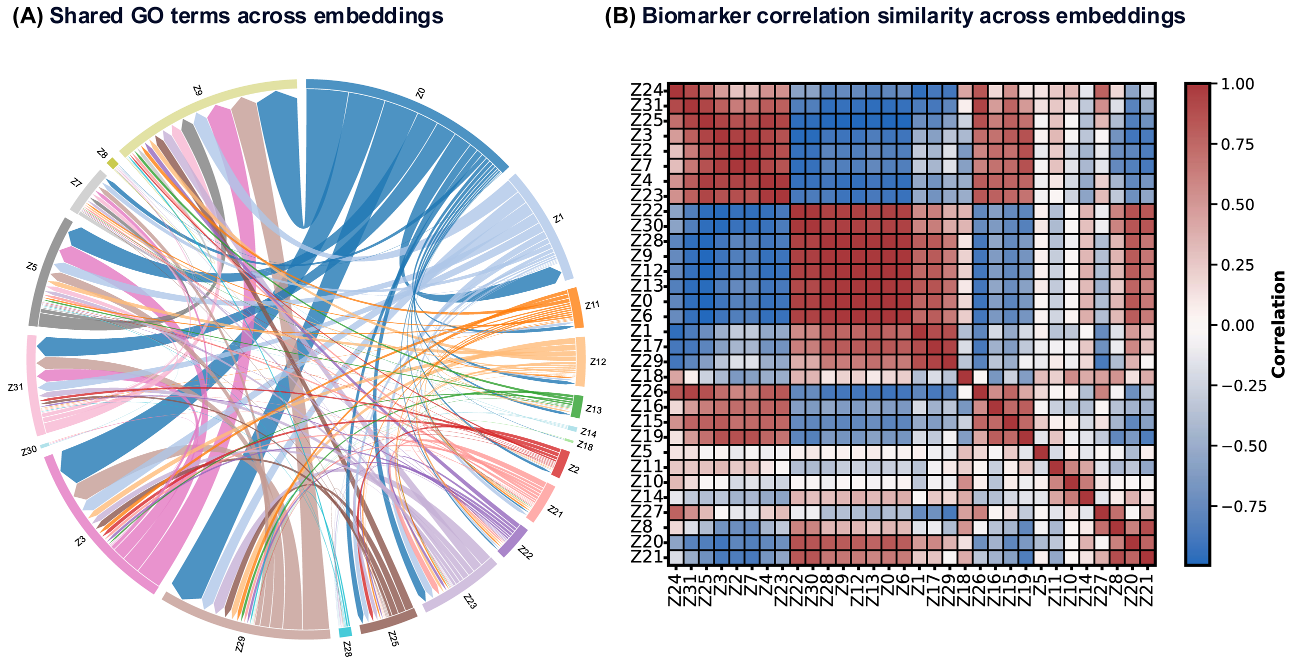

Figure S5. Global view of embeddings. (A) Network of embeddings connected by shared Gene Ontology (GO) terms. Nodes represent individual embeddings; edges indicate overlap in GO-term annotations, and edge width is proportional to the number of shared GO terms between the connected embeddings (larger width = greater overlap). (B) Similarity of embeddings based on biomarker-correlation profiles. For each embedding, we computed its correlation with the 21 biomarkers shown in Fig. 4C, yielding a 21-element correlation vector per embedding; pairwise embedding similarities were then calculated as the correlation between these correlation vectors.

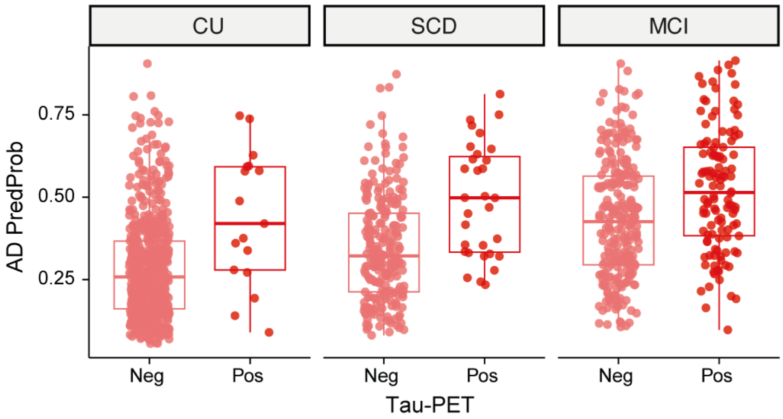

Figure S6. AD probabilities by TauPET positivity across diagnostic groups on the BioFINDER-2 cohort. Box plots were stratified by Tau PET status within each clinical group (CU, SCD, MCI), counts were reported for Tau PET+ and Tau PET− (19/585, 36/223, 136/239, respectively). Whiskers represented 1.5 times the interquartile range.

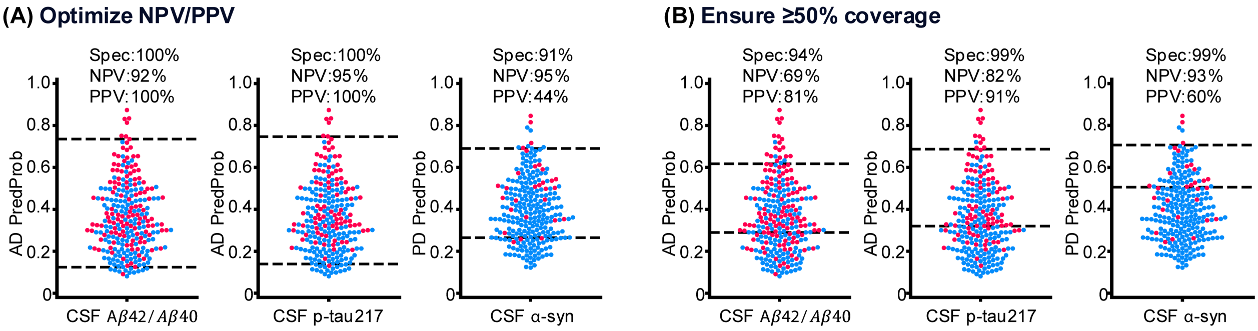

Figure S7. Two cutoff strategies for predicting biomarker positivity. Cutoffs were fit in non-SCD participants (n=1,524) from BioFINDER-2 and then applied to individuals with SCD (n=263) to estimate out-of-sample predictive performance. (A) Cutoffs optimized to maximize positive predictive value (PPV) and negative predictive value (NPV). (B) Cutoffs constrained to achieve ≥50% coverage.

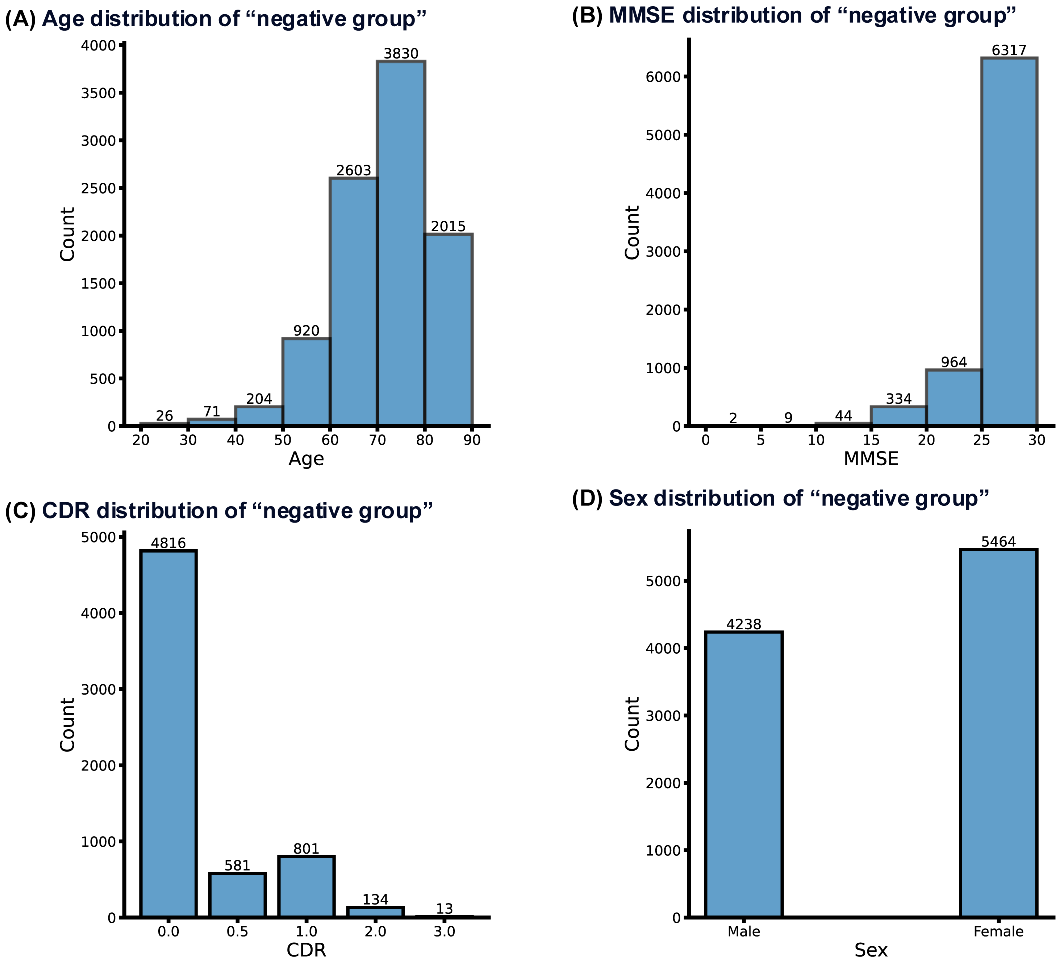

Figure S8. Distribution of “negative group” (N=9,708). (A) Age distribution of “negative group”. (B) MMSE distribution of “negative group”. (C) CDR distribution of “negative group”. (D) Sex distribution of “negative group”.

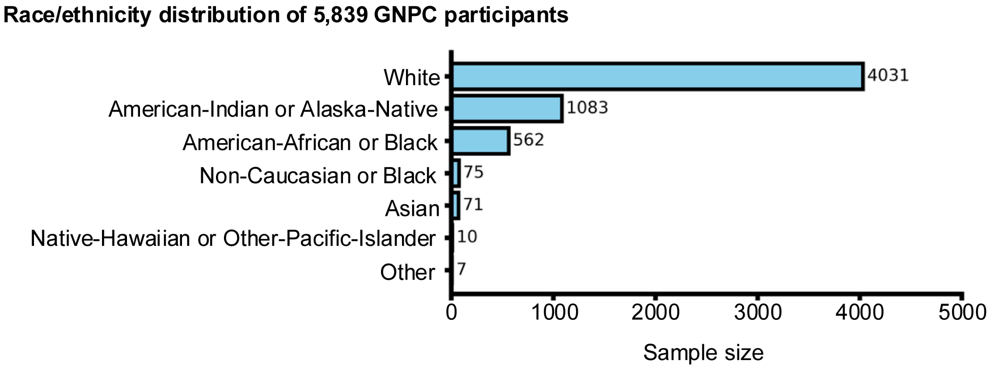

Figure S9. Distribution of race and ethnicity among 5,839 GNPC participants. As race and ethnicity were not primary variables during the GNPC collection stage, this information was available for only 5,839 participants.

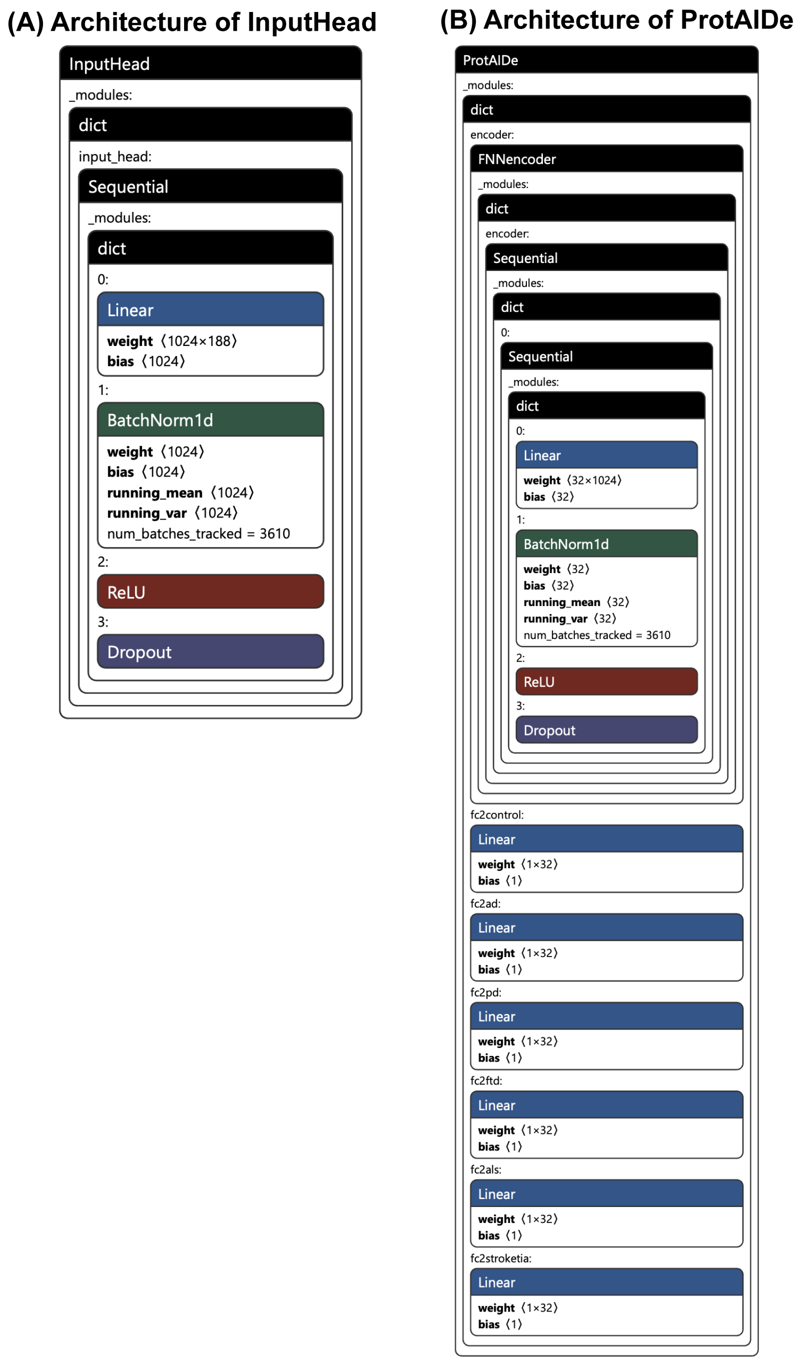

Figure S10. Architecture of ProtAIDe-Dx (model evaluated on the BioFINDER-2 cohort). The model comprises two MLP-based modules: (A) InputHead, which ingests proteins and produces initial input embeddings; and (B) ProtAIDe, which refines input embeddings and outputs probabilistic predictions for six conditions. Network components: Linear (affine transformation), BatchNorm1D (batch normalization over the feature dimension), ReLU (rectified linear unit), and Dropout (randomly zeroing activations with probability p). All ProtAIDe-Dx models share this architecture but differ in hyperparameters (e.g., number of layers, node of layer, dropout rate).

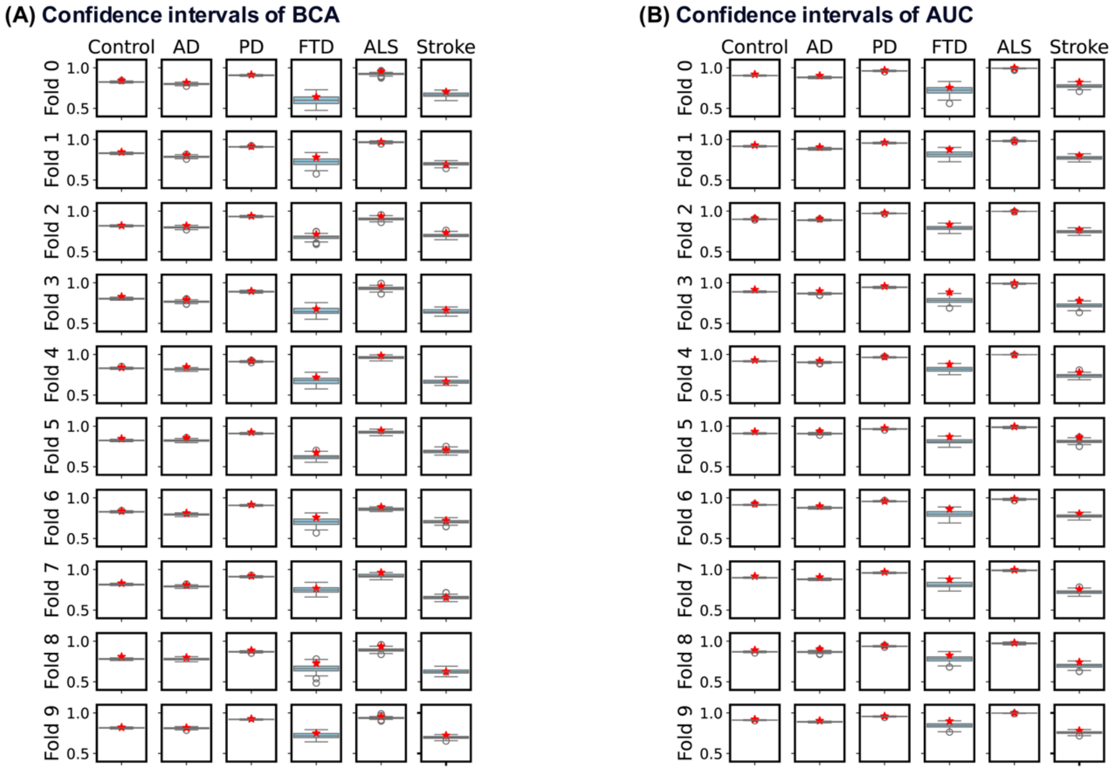

Figure S11. Assessment of overfitting using confidence intervals of predictions on test splits. The width of the confidence interval can be understood through the bias–variance tradeoff as a proxy of overfitting. Specifically, an overfitted model achieves low bias by memorizing the training data, producing precise predictions on training data. However, this comes at the cost of high variance: the model's predictions become highly sensitive to small fluctuations in the training set. When applied to new test data, this instability results in inaccurate and inconsistent predictions, which manifest as wider confidence intervals on the test set, reflecting greater predictive uncertainty. (A) Confidence intervals of BCA. (B) Confidence intervals of AUC. Box plots were drawn on 100 forward passes enabling random drop out, showing the median, interquartile range (25th–75th percentiles), and whiskers extending to 1.5×IQR.

Supplementary Tables

Site	Sample Size	Age (Mean ± Std)	Sex (%Female)	BMI (Mean ± Std)	MMSE (Mean ± Std)
A	983	72.1±6.7	56.4%	27.9±5.6	26.6±2.9
B	443	57.0±4.9	60.3%	26.8±4.1	29.1±1.0
C	2000	68.8±11.7	48.8%	25.9±4.0	26.8±3.8
D	489	71.3±9.6	60.1%	-	25.5±5.5
E	537	72.6±7.2	57.2%	27.4±4.8	27.5±3.6
F	3355	74.7±9.8	56.7%	27.5±5.3	26.4±4.6
G	1330	67.3±14.0	52.3%	-	28.5±1.7
I	545	66.6±7.8	58.7%	28.4±5.7	-
J	421	71.3±8.4	51.5%	26.0±4.7	-
K	406	71.8±5.5	50.4%	26.2±4.3	19.8±1.1
L	1101	78.8±8.8	58.9%	26.4±5.2	26.2±5.1
M	355	57.6±12.0	43.9%	27.3±5.0	-
N	789	56.1±14.7	53.6%	26.9±5.8	-
P	519	71.7±8.7	44.5%	-	28.3±1.4
Q	1370	72.2±8.8	57.9%	26.9±4.1	24.5±4.5
R	435	82.7±5.7	71.0%	-	22.7±8.2
S	200	60.0±9.8	50.0%	-	-
T	233	66.5±8.3	65.2%	26.4±4.5	28.2±1.7
U	1676	67.7±9.1	34.8%	-	-

Table S1. Demographics and cognition distribution of the selected 17,187 GNPC v1.3MS participants by site. The item with "-" means this variable is missing.

Site	Sample Size	%Control	%AD	%PD	%FTD	%ALS	%Stroke	%TIA
A	983	38.6%	26.2%	0.1%	0	0	3.2%	5.1%
B	443	80.8%	0	0	0	0	0	0
C	2000	36.2%	13.2%	6.4%	2.4%	0	0	0
D	489	28.4%	55.8%	0.8%	0	0	0	0
E	537	57.5%	10.8%	0.4%	0.7%	0	0.4%	2.2%
F	3355	45.5%	29.2%	3.5%	0.9%	0	2.6%	0
G	1330	69.7%	10.5%	0	0	0	1.1%	0
I	545	42.9%	35.2%	0.2%	2.8%	0	0	0.7%
J	421	42.3%	22.1%	24.0%	0	0	0.7%	2.6%
K	406	0	0	0	0	0	0	0
L	1101	47.1%	11.8%	6.2%	0	0	10.7%	11.3%
M	355	30.1%	0	0	0.3%	69.0%	1.7%	3.1%
N	789	48.7%	0.4%	0.1%	12.5%	1.1%	0	0
P	519	86.9%	0	0	0	0	1.5%	0
Q	1370	25.0%	0.3%	3.9%	0.7%	0	0	0
R	435	26.4%	31.0%	4.8%	0	0	23.4%	0
S	200	0	0	0	0	95.0%	0	0
T	233	0	0	100%	0	0	0	0
U	1676	0	0	100%	0	0	0	0

Table S2. Positive ratio of clinical diagnosis of the selected 17,187 GNPC v1.3MS. The item with "-" means this variable is missing, and the item with 0 means all participants are negative. The disease diagnostic categories were not exclusive, and some participants missed diagnostic information for six conditions, therefore, the sum of positive ratios was not equal to 1.

Metric	Model	**Control** mean±std median	**AD** mean±std median	**PD** mean±std median	**FTD** mean±std median	**ALS** mean±std median	**Stroke** mean±std median
BCA	RandomForest	0.81±0.01 0.80	0.65±0.02 0.65	0.89±0.01 0.89	0.50±0 0.50	0.81±0.03 0.82	0.54±0.02 0.54
	XGBoost	0.83±0.01 0.83	0.78±0.02 0.78	0.92±0.01 0.92	0.55±0.03 0.55	0.95±0.02 0.94	0.60±0.03 0.61
	TabPFN	0.84±0.01 0.84	0.82±0.02 0.82	0.92±0.01 0.92	0.64±0.06 0.63	0.95±0.04 0.96	0.70±0.04 0.70
	ProtAIDe-Dx	0.83±0.01 0.83	0.82±0.02 0.81	0.92±0.01 0.92	0.72±0.04 0.72	0.95±0.03 0.95	0.69±0.03 0.70
	Ensemble	0.85±0.01 0.84	0.82±0.01 0.82	0.92±0.01 0.93	0.73±0.03 0.74	0.95±0.03 0.96	0.70±0.03 0.71
AUC	RandomForest	0.81±0.01 0.80	0.65±0.02 0.65	0.89±0.01 0.89	0.50±0 0.50	0.81±0.03 0.82	0.54±0.02 0.54
	XGBoost	0.92±0.01 0.91	0.91±0.01 0.91	0.97±0.01 0.97	0.84±0.02 0.84	0.99±0.01 1.00	0.78±0.04 0.79
	TabPFN	0.92±0.01 0.92	0.92±0.01 0.92	0.97±0.01 0.97	0.86±0.04 0.86	0.96±0.00 1.00	0.82±0.03 0.82
	ProtAIDe-Dx	0.92±0.01 0.92	0.91±0.01 0.90	0.97±0.01 0.97	0.86±0.04 0.87	0.99±0.01 1.00	0.79±0.03 0.78
	Ensemble	0.93±0.01 0.92	0.91±0.01 0.91	0.97±0.01 0.97	0.86±0.04 0.87	0.99±0.01 1.00	0.79±0.03 0.78

Table S3. BCA and AUC scores for 10-fold cross-validation. Mean, standard deviation, and median values were computed from 10 test folds.

Metric	Model	**Control** mean±std median	**AD** mean±std median	**PD** mean±std median	**FTD** mean±std median	**ALS** mean±std median	**Stroke** mean±std median
BCA	RandomForest	0.59±0.09 0.60	0.56±0.03 0.56	0.51±0.02 0.50	0.50±0 0.50	0.58±0.08 0.58	0.50±0.01 0.50
	XGBoost	0.64±0.07 0.64	0.67±0.05 0.67	0.58±0.04 0.57	0.50±0 0.50	0.56±0.04 0.56	0.51±0.01 0.50
	TabPFN	0.66±0.09 0.68	0.73±0.06 0.71	0.60±0.06 0.59	0.52±0.02 0.51	0.61±0.10 0.61	0.54±0.05 0.53
	ProtAIDe-Dx	0.67±0.09 0.68	0.73±0.06 0.74	0.62±0.07 0.60	0.69±0.04 0.68	0.56±0.06 0.56	0.59±0.06 0.59
	Ensemble	0.66±0.09 0.68	0.72±0.04 0.71	0.62±0.07 0.60	0.66±0.09 0.65	0.56±0.05 0.56	0.57±0.06 0.57
	Retrain	0.61±0.05 0.63	0.64±0.04 0.64	0.61±0.07 0.60	0.61±0.08 0.62	0.56±0.06 0.56	0.53±0.03 0.52
	Finetune	0.68±0.08 0.71	0.74±0.05 0.72	0.64±0.07 0.64	0.62±0.05 0.61	0.60±0.00 0.60	0.56±0.05 0.57
AUC	RandomForest	0.59±0.09 0.60	0.56±0.03 0.56	0.51±0.02 0.50	0.50±0 0.50	0.58±0.08 0.58	0.50±0.01 0.50
	XGBoost	0.72±0.08 0.74	0.80±0.07 0.83	0.71±0.10 0.73	0.63±0.06 0.64	0.74±0.02 0.74	0.57±0.07 0.57
	TabPFN	0.75±0.09 0.79	0.83±0.06 0.85	0.76±0.12 0.77	0.66±0.04 0.64	0.77±0.02 0.77	0.61±0.08 0.60
	ProtAIDe-Dx	0.74±0.08 0.78	0.81±0.06 0.82	0.71±0.09 0.69	0.72±0.03 0.72	0.66±0.03 0.66	0.61±0.02 0.62
	Ensemble	0.75±0.10 0.78	0.81±0.06 0.83	0.72±0.09 0.74	0.72±0.03 0.71	0.72±0.04 0.72	0.61±0.04 0.62
	Retrain	0.68±0.08 0.70	0.72±0.07 0.73	0.67±0.09 0.69	0.65±0.13 0.67	0.69±0.06 0.69	0.57±0.06 0.55
	Finetune	0.73±0.10 0.76	0.80±0.07 0.80	0.70±0.08 0.70	0.67±0.05 0.65	0.63±0.00 0.63	0.59±0.09 0.60

Table S4. BCA and AUC scores for leave-one-site-out. Mean, standard deviation, and median values were computed from 14 test sites.

Target	Relative Performances (Test / Development)
Control	84%±12%
AD	87%±10%
PD	89%±13%
FTD	96%±16%
ALS	90%±4%
Stroke	100%±12%

Table S5. Mean and standard deviation of relative performances from site-to-site generalization experiment. The 14 test sites were the same as in the leave-one-site-out setting.

Variable	True Negative	False Positive	Adjusted P Values
Age	60.4±15.7	78.6±6.7	4e-38
Sex (% Female)	60.1%	56.7%	0.59
CSF abnormal Aβ42/Aβ40 ratio	14.8%	44.8%	1e-8
CSF p-tau 217	6.27±6.73	13.2±12.9	6e-5
TauPET	1.08±0.11	1.19±0.20	3e-5
ADSignCT	2.76±0.12	2.67±0.13	8e-7
WholeBrainCT	2.40±0.08	2.32±0.07	2e-10
Ventricle Volume	0.009±0.006	0.014±0.001	1e-8
WMH	0.003±0.003	0.006±0.004	3e-7

Table S6. Distribution of demographics and AD biomarkers between the True Negative and False Positive groups on the BioFIDNER-2 cohort. The true positive group (N=459) referred to Control participants predicted as Control, and the false positive group (N=67) referred to Control participants predicted as AD patients. For binary variables, p values were computed by the two-proportion z test; for continuous variables, p values were computed by the t-test. All p values were FDR-corrected.

Model	AD	PD	FTD	Stroke
M0	0.60±0.02	0.57±0.03	0.51±0.03	0.54±0.05
M1	0.63±0.02	0.62±0.03	0.54±0.03	0.67±0.06
M2	0.84±0.02	0.67±0.03	0.72±0.04	0.60±0.06
M3	0.84±0.02	0.76±0.03	0.81±0.04	0.63±0.06

Table S7. Models' BCA (mean±std) on differentiating diagnoses on the BioFINDER-2 cohort. The mean and standard deviations were computed on 1000 bootstraps of testing participants.

Model Pair		FDR-corrected P values			
Model A	Model B	AD	PD	FTD	Stroke
M0	M1	2e-118	4e-296	5e-64	0
M0	M2	0	0	8e-190	9e-30
M0	M3	0	0	0	9e-79
M1	M2	0	0	0	4e-212
M1	M3	0	2e-17	0	9e-22
M2	M3	0	0	0	5e-124

Table S8. FDR-corrected p values by comparing the models' BCA on differentiating diagnoses on the BioFINDER-2 dataset. The p values were computed on 1000 bootstraps with t-test. The BCA was defined as one-versus-rest.

	Coef	Std.Err	Z	P value	Adjusted P value
Intercept	19.73	0.37	53.33	4.72e-01	6.12e-01
C(Sex)	-0.06	0.09	-0.72	9.30e-01	9.30e-01
C(Site)[D]	0.02	0.22	0.09	1.31e-02	2.61e-02
C(Site)[E]	0.56	0.23	2.48	1.22e-02	2.61e-02
C(Site)[F]	0.40	0.16	2.51	6.39e-01	7.20e-01
C(Site)[P]	-0.06	0.13	-0.47	8.92e-04	2.38e-03
C(Site)[R]	-1.05	0.31	-3.32	8.92e-02	1.59e-01
C(Site)[T]	-0.99	0.58	-1.70	9.44e-141	7.55e-140
C(BlDx)[Control]	9.13	0.36	25.26	2.22e-49	8.89e-49
C(BlDx)[PD]	9.81	0.66	14.77	5.01e-50	2.67e-49
C(BlDx)[StrokeTIA]	8.34	0.56	14.87	4.36e-09	1.40e-08
Age	-0.36	0.06	-5.87	4.54e-01	6.12e-01
Year	-0.08	0.11	-0.75	4.97e-01	6.12e-01
C(BlDx)[Control]:Year	0.08	0.11	0.68	2.33e-01	3.73e-01
C(BlDx)[PD]:Year	-0.15	0.13	-1.19	6.75e-01	7.20e-01
C(BlDx)[StrokeTIA]:Year	-0.07	0.16	-0.42	4.72e-01	6.12e-01

Table S9. Linear mixed-effect model results for modeling cognitive decline by baseline diagnosis on GNPC. MMSE ~ Age + Sex + Site + BaselineDx * Year + Year|SubjID. BlDx was short for BaselineDx. Fixed effects: Age, Sex, Site and the interaction between baseline diagnosis and time (Year); random intercepts and slopes for Year were included for each subject. The BaselineDx × Year term tests whether annual change in MMSE differs by baseline diagnosis.

	Coef	Std.Err	Z	P value	Adjusted P value
Intercept	19.22	0.40	47.74	0	0
C(Sex)	-0.08	0.09	-0.93	3.51e-01	4.76e-01
C(Site)[D]	0.13	0.22	0.60	5.48e-01	6.80e-01
C(Site)[E]	0.54	0.22	2.40	1.63e-02	2.81e-02
C(Site)[F]	0.41	0.16	2.57	1.01e-02	1.92e-02
C(Site)[P]	-0.07	0.13	-0.51	6.09e-01	6.80e-01
C(Site)[R]	-0.57	0.54	-1.06	2.88e-01	4.21e-01
C(Site)[T]	-0.12	0.83	-0.14	8.87e-01	8.87e-01
C(BlDx)[Control]	8.76	0.37	23.89	3.69e-126	3.51e-125
C(BlDx)[PD]	8.79	0.65	13.49	1.8e-41	8.82e-41
C(BlDx)[StrokeTIA]	7.83	0.54	14.43	3.13e-47	1.98e-46
C(BlPred)[Control]	0.92	0.33	2.81	4.96e-03	1.30e-02
C(BlPred)[PD]	0.38	0.73	0.52	6.05e-01	6.80e-01
C(BlPred)[StrokeTIA]	0.11	0.66	0.16	8.72e-01	8.87e-01
Age	-0.34	0.06	-5.51	3.65e-08	1.39e-07
Year	-0.31	0.11	-2.88	3.97e-03	1.26e-02
C(BlPred)[Control]:Year	0.29	0.11	2.74	6.18e-03	1.30e-02
C(BlPred)[PD]:Year	0.33	0.15	2.20	2.77e-02	4.39e-02
C(BlPred)[StrokeTIA]:Year	0.37	0.14	2.76	5.74e-03	1.30e-02

Table S10. Linear mixed-effect model results for modeling cognitive decline by baseline prediction on GNPC. MMSE ~ Age + Sex + Site + BaselineDx + BaselinePrediction * Year + Year|SubjID. BlDx was short for BaselineDx, and BlPred was short for BaselinePrediction. Fixed-effects: Age, Sex, Site, BaselineDx, and the interaction between baseline prediction and time (Year); random intercepts and slopes for Year were included for each subject. The BaselinePrediction × Year term tests whether annual change in MMSE differs by baseline prediction.

	Coef	Std.Err	Z	P value	Adjusted P value
Intercept	28.43	1.17	24.21	1.59e-129	9.57e-129
C(Sex)	-0.55	0.24	-2.28	2.28e-02	2.73e-02
C(BlPred)[Control]	0.80	0.26	3.12	1.80e-03	2.70e-03
Age	-0.02	0.02	-1.23	2.17e-01	2.17e-01
Year	-1.62	0.15	-10.65	1.80e-26	5.41e-26
C(BlPred)[Control]:Year	0.73	0.22	3.37	7.48e-04	1.50e-03

Table S11. Linear mixed-effect model results for modeling cognitive decline by baseline prediction on BioFINDEER-2 MCI patients. MMSE ~ Age + Sex + BaselinePrediction * Year + Year|SubjID. BlPred was short for BaselinePrediction. Fixed-effects: Age, Sex, Site, and the interaction between baseline prediction and time (Year); random intercepts and slopes for Year were included for each subject. The BaselinePrediction × Year term tests whether annual change in MMSE differs by baseline prediction.

Diagnosis Group	Sample size	Age (Mean ± Std)	Sex (n,%Female)	MMSE (Mean ± Std)
CU	609	63.6±15.8	350 (57.4%)	29.0±1.1
SCD	263	68.0±9.0	133 (50.6%)	28.7±1.5
MCI	381	71.7±7.9	162 (42.5%)	27.1±1.9
AD dementia	263	73.8±7.4	147 (55.9%)	21.0±4.2
Parkinsonism	190	71.0±8.8	52 (27.4%)	25.8±4.5
Other diseases	80	72.0±8.8	36 (45.0%)	24.0±4.1

Table S12. Demographics and cognition distribution of 1,786 BioFINDER-2 participants.

Site	Control (Pos-Neg)	AD (Pos-Neg)	PD (Pos-Neg)	FTD (Pos-Neg)	ALS (Pos-Neg)	Stroke/TIA (Pos-Neg)
A	379-250	214-412	X	X	X	57-572
C	723-399	224-898	127-995	48-1074	X	X
D	139-126	125-76	X	X	X	X
E	309-76	X	X	X	X	14-313
F	1527-780	690-1617	99-2208	25-2282	X	74-2233
G	927-40	26-941	X	X	X	X
I	234-206	192-133	X	X	X	X
J	178-201	93-285	101-278	X	X	14-347
L	519-308	113-128	65-283	X	X	192-510
M	107-248	X	X	X	245-110	11-344
N	384-112	X	X	99-397	9-487	X
P	451-8	X	X	X	X	X
Q	343-66	X	54-355	10-399	X	X
R	115-191	126-180	20-285	X	X	X

Table S13. Sample size by each target for selected testing sites in GNPC. The 'X' means the target of this contributor did not pass the selection criteria.

Hyperparameter	Range
Node of input layer	16, 32, 64, 128, 256, 512, 1024
Number of hidden layers	1, 2, 3
Node of each hidden layer	8, 16, 32, 64, 128, 256, 512
Learning rate	1e-4 – 1e-1
Dropout	0 - 0.5
Optimizer	Adam, SGD, RMSProp
λ	0 - 5
α	0 - 0.2

Table S14. Hyperparameter search range for ProtAIDe-Dx model.

Hyperparameter	Optimal value
Node of input layer	1024
Number of hidden layers	1
Node of each hidden layer	32
Learning rate	1e-4
Dropout	0.4
Optimizer	Adam
lambda	3
alpha_Control	0.1
alpha_AD	0.1
alpha_PD	0.1
alpha_FTD	0.1
alpha_ALS	0
alpha_Stroke	0.1

Table S15. Optimal hyperparameters of the ProtAIDe-Dx model evaluated on the BioFINDER-2 cohort.

Supplementary References

1. Borghammer, P. *et al.* Cortical hypometabolism and hypoperfusion in Parkinson's disease is extensive: probably even at early disease stages. *Brain Struct. Funct.* **214**, 303–317 (2010).
2. Labrador-Espinosa, M. A. *et al.* Cortical hypometabolism in Parkinson's disease is linked to cholinergic basal forebrain atrophy. *Mol. Psychiatry* **30**, 2372–2380 (2025).
3. Corriveau-Lecavalier, N., Adams, J. N., Fischer, L., Molloy, E. N. & Maass, A. Cerebral hyperactivation across the Alzheimer's disease pathological cascade. *Brain Commun.* **6**, fcae376 (2024).
4. Rutledge, J. *et al.* Comprehensive proteomics of CSF, plasma, and urine identify DDC and other biomarkers of early Parkinson's disease. *Acta Neuropathol.* **147**, 52 (2024).
5. Buono, M. & Cosma, M. P. Sulfatase activities towards the regulation of cell metabolism and signaling in mammals. *Cell. Mol. Life Sci.* **67**, 769–780 (2010).
6. Frankel, L. B. *et al.* A non-conserved miRNA regulates lysosomal function and impacts on a human lysosomal storage disorder. *Nat. Commun.* **5**, 5840 (2014).
7. Udayar, V., Chen, Y., Sidransky, E. & Jagasia, R. Lysosomal dysfunction in neurodegeneration: emerging concepts and methods. *Trends Neurosci.* **45**, 184–199 (2022).
8. Winchester, L. *et al.* Identification of a possible proteomic biomarker in Parkinson's disease: discovery and replication in blood, brain and cerebrospinal fluid. *Brain Commun.* **5**, fcac343 (2023).
9. Ramos-Martinez, E., Ramos-Martínez, I., Molina-Salinas, G., Zepeda-Ruiz, W. A. & Cerbon, M. The role of prolactin in central nervous system inflammation. *Rev. Neurosci.* **32**, 323–340 (2021).
10. Agnoli, A. *et al.* Prolactin response as an index of dopaminergic receptor function in Parkinson's disease. Correlation with clinical findings and therapeutic response. *J. Neural Transm.* **51**, 123–134 (1981).
11. Ben-Jonathan, N. & Hnasko, R. Dopamine as a prolactin (PRL) inhibitor. *Endocr. Rev.* **22**, 724–763 (2001).
12. Kostic, V. S., Marinkovic, Z., Filipovic, S. & Momcilovic, D. Function of dopamine receptors in young-onset Parkinson's disease: prolactin response. *Mov. Disord.* **8**, 227–229 (1993).
13. Daborg, J. *et al.* Cerebrospinal fluid levels of complement proteins C3, C4 and CR1 in Alzheimer's disease. *J. Neural Transm. (Vienna)* **119**, 789–797 (2012).
14. Veselý, B. *et al.* Interleukin 6 and complement serum level study in Parkinson's disease. *J. Neural Transm. (Vienna)* **125**, 875–881 (2018).
15. Abdi, I. Y., Ghanem, S. S. & El-Agnaf, O. M. Immune-related biomarkers for Parkinson's disease. *Neurobiol. Dis.* **170**, 105771 (2022).
16. Hällqvist, J. *et al.* Plasma proteomics identify biomarkers predicting Parkinson's disease up to 7 years before symptom onset. *Nat. Commun.* **15**, 4759 (2024).
17. Dick, F. *et al.* Brain proteome profiling reveals common and divergent signatures in Parkinson's disease, multiple system atrophy, and progressive supranuclear palsy. *Mol. Neurobiol.* **62**, 2801–2816 (2025).
18. Baytas, O., Kauer, J. A. & Morrow, E. M. Loss of mitochondrial enzyme GPT2 causes early neurodegeneration in locus coeruleus. *Neurobiol. Dis.* **173**, 105831 (2022).
19. Xu, Y. *et al.* Chronic cadmium exposure induces Parkinson-like syndrome by eliciting sphingolipid disturbance and neuroinflammation in the midbrain of C57BL/6J mice. *Environ. Pollut.* **337**, 122606 (2023).
20. Gu, X. *et al.* Enrichment of rare variants in E3 ubiquitin ligase genes in Early onset Parkinson's disease. *Neurobiol. Aging* **109**, 273–278 (2022).
21. Beric, A. *et al.* Plasma acellular transcriptome contains Parkinson's disease signatures that can inform clinical diagnosis. *medRxiv* 2024.10.18.24315717 (2024) doi:10.1101/2024.10.18.24315717.
22. Nilsson, J. *et al.* Cerebrospinal fluid biomarkers of synaptic dysfunction are altered in Parkinson's disease and related disorders. *Mov. Disord.* **38**, 267–277 (2023).
23. Bartl, M. *et al.* Author Correction: Lysosomal and synaptic dysfunction markers in longitudinal cerebrospinal fluid of de novo Parkinson's disease. *NPJ Parkinsons Dis.* **10**, 121 (2024).
24. Oh, H. S.-H. *et al.* A cerebrospinal fluid synaptic protein biomarker for prediction of cognitive resilience versus decline in Alzheimer's disease. *Nat. Med.* (2025) doi:10.1038/s41591-025-03565-2.
25. Frigerio, I. *et al.* Regional differences in synaptic degeneration are linked to alpha-synuclein burden and axonal damage in Parkinson's disease and dementia with Lewy bodies. *Acta Neuropathol. Commun.* **12**, 4 (2024).
26. Holmes, S. E. *et al.* Synaptic loss and its association with symptom severity in Parkinson's disease. *NPJ Parkinsons Dis.* **10**, 42 (2024).
27. Al-Kuraishy, H. M., Jabir, M. S., Al-Gareeb, A. I. & Albuhadily, A. K. The conceivable role of prolactin

hormone in Parkinson disease: The same goal but with different ways. *Ageing Res. Rev.* **91**, 102075 (2023).
28. Nguyen, H. D., Jo, W. H., Hoang, N. H. M. & Kim, M.-S. In silico identification of the potential molecular mechanisms involved in protective effects of prolactin on motor and memory deficits induced by 1,2-Diacetylbenzene in young and old rats. *Neurotoxicology* **93**, 45–59 (2022).
29. Wang, S. *et al.* Association of cerebrospinal fluid ciliary neurotrophic factor levels with cognitive decline and disease progression. *J. Alzheimers. Dis.* **105**, 573–581 (2025).
30. Gudkova, A. A., Druzhkova, T. A. & Zinchuk, M. S. Levels of brain-derived neurotrophic factor (BDNF) and ciliary neurotrophic factor (CNTF) in blood and tears of young and middle-aged patients with vascular cognitive impairment and depression. *Neurochem. J.* **18**, 243–249 (2024).
31. Oh, H. S.-H. *et al.* Organ aging signatures in the plasma proteome track health and disease. *Nature* **624**, 164–172 (2023).
32. Broersen, L. H. A. *et al.* High-sensitivity cardiac troponin T and cognitive function in patients with ischemic stroke. *Stroke* **51**, 1604–1607 (2020).
33. Chen, Y. *et al.* High-sensitivity cardiac troponin I and risk of dementia: 25-year longitudinal study in the Whitehall II cohort. *medRxiv* (2024) doi:10.1101/2024.11.19.24317589.
34. Dergai, O. *et al.* Skeletal muscle biomarkers of amyotrophic lateral sclerosis: a large-scale, multi-cohort proteomic study. *medRxiv* 2025.04.23.25326161 (2025) doi:10.1101/2025.04.23.25326161.
35. Bongioanni, P., Reali, C. & Sogos, V. Ciliary neurotrophic factor (CNTF) for amyotrophic lateral sclerosis/motor neuron disease. *Cochrane Database Syst. Rev.* **2011**, CD004302 (2004).
36. Donaldson, D. H., Britt, D. E., Jones, C., Jackson, C. L. & Patterson, D. Localization of the gene for the ciliary neurotrophic factor receptor (CNTFR) to human chromosome 9. *Genomics* **17**, 782–784 (1993).
37. Yiş, U. *et al.* Clinical, radiological, and genetic survey of patients with muscle-eye-brain disease caused by mutations in POMGNT1. *Pediatr. Neurol.* **50**, 491–497 (2014).
38. Biancheri, R. *et al.* POMGnT1 mutations in congenital muscular dystrophy: genotype-phenotype correlation and expanded clinical spectrum. *Arch. Neurol.* **63**, 1491–1495 (2006).
39. Yu, Y. *et al.* Lrtm1: A novel sensor of insulin signaling and regulator of metabolism and activity. *Diabetes* **74**, 691–704 (2025).
40. Molinaro, P. *et al.* Emerging role of DREAM in healthy brain and neurological diseases. *Int. J. Mol. Sci.* **24**, 9177 (2023).
41. Wu, L.-Y., Song, Y.-J., Zhang, C.-L. & Liu, J. KV channel-interacting proteins in the neurological and cardiovascular systems: An updated review. *Cells* **12**, 1894 (2023).
42. Arreola-Aldape, C. A. *et al.* A systematic review and functional in-silico analysis of genes and variants associated with amyotrophic lateral sclerosis. *Front. Neurosci.* **19**, 1598336 (2025).
43. Larrodé, P. *et al.* DREAM-dependent activation of astrocytes in amyotrophic lateral sclerosis. *Mol. Neurobiol.* **55**, 1–12 (2018).
44. Bączyk, M. *et al.* Synaptic restoration by cAMP/PKA drives activity-dependent neuroprotection to motoneurons in ALS. *J. Exp. Med.* **217**, (2020).
45. Gonzalo-Gobernado, R. *et al.* Repaglinide induces ATF6 processing and neuroprotection in transgenic SOD1G93A mice. *Int. J. Mol. Sci.* **24**, 15783 (2023).
46. Dimitriadi, M. *et al.* The neuroprotective drug riluzole acts via small conductance Ca2+-activated K+ channels to ameliorate defects in spinal muscular atrophy models. *J. Neurosci.* **33**, 6557–6562 (2013).
47. Yang, D. *et al.* FTD/ALS-associated poly(GR) protein impairs the Notch pathway and is recruited by poly(GA) into cytoplasmic inclusions. *Acta Neuropathol.* **130**, 525–535 (2015).
48. Giannini, L. A. A. *et al.* Clinical value of longitudinal serum neurofilament light chain in prodromal genetic frontotemporal dementia. *Neurology* **101**, e1069–e1082 (2023).
49. Ashton, N. J. *et al.* A multicentre validation study of the diagnostic value of plasma neurofilament light. *Nat. Commun.* **12**, 3400 (2021).
50. Rohrer, J. D. *et al.* Serum neurofilament light chain protein is a measure of disease intensity in frontotemporal dementia. *Neurology* **87**, 1329–1336 (2016).
51. Sogorb-Esteve, A. *et al.* Proteomic analysis reveals distinct cerebrospinal fluid signatures across genetic frontotemporal dementia subtypes. *Sci. Transl. Med.* **17**, eadm9654 (2025).
52. Saloner, R. *et al.* Large-scale network analysis of the cerebrospinal fluid proteome identifies molecular signatures of frontotemporal lobar degeneration. *Res. Sq.* (2024) doi:10.21203/rs.3.rs-4103685/v1.
53. Tanaka, T. *et al.* Plasma proteomic signatures predict dementia and cognitive impairment. *Alzheimers Dement. (N. Y.)* **6**, e12018 (2020).
54. Guo, Y. *et al.* Plasma proteomic profiles predict future dementia in healthy adults. *Nat. Aging* **4**, 247–260

(2024).
55. Small, D. M. *et al.* A functional variant of elafin with improved anti-inflammatory activity for pulmonary inflammation. *Mol. Ther.* **23**, 24–31 (2015).
56. Bright, F. *et al.* Neuroinflammation in frontotemporal dementia. *Nat. Rev. Neurol.* **15**, 540–555 (2019).
57. McCauley, M. E. & Baloh, R. H. Inflammation in ALS/FTD pathogenesis. *Acta Neuropathol.* **137**, 715–730 (2019).
58. Prudencio, M. *et al.* Distinct brain transcriptome profiles in C9orf72-associated and sporadic ALS. *Nat. Neurosci.* **18**, 1175–1182 (2015).
59. Bergström, S. *et al.* A panel of CSF proteins separates genetic frontotemporal dementia from presymptomatic mutation carriers: a GENFI study. *Mol. Neurodegener.* **16**, 79 (2021).
60. Reich, M. *et al.* Peripheral expression of brain-penetrant progranulin rescues pathologies in mouse models of frontotemporal lobar degeneration. *Sci. Transl. Med.* **16**, eadj7308 (2024).
61. Tesla, R. *et al.* Benzoxazole-derivatives enhance progranulin expression and reverse the aberrant lysosomal proteome caused by GRN haploinsufficiency. *Nat. Commun.* **15**, 6125 (2024).
62. Arcos, J. *et al.* IGF2 prevents dopaminergic neuronal loss and decreases intracellular alpha-synuclein accumulation in Parkinson's disease models. *Cell Death Discov.* **9**, 438 (2023).
63. Liou, C. J., Tong, M., Vonsattel, J. P. & de la Monte, S. M. Altered brain expression of insulin and insulin-like growth factors in frontotemporal lobar degeneration: Another degenerative disease linked to dysregulation of insulin metabolic pathways. *ASN Neuro* **11**, 1759091419839515 (2019).
64. Alberini, C. M. IGF2 in memory, neurodevelopmental disorders, and neurodegenerative diseases. *Trends Neurosci.* **46**, 488–502 (2023).
65. Kavanagh, T. *et al.* The interactome of tau phosphorylated at T217 in Alzheimer's disease human brain tissue. *Acta Neuropathol.* **149**, 44 (2025).
66. Shwab, E. K. *et al.* Comparative mapping of single-cell transcriptomic landscapes in neurodegenerative diseases. *Alzheimers. Dement.* **21**, e70012 (2025).
67. Shahim, P. *et al.* Cerebrospinal fluid stanniocalcin-1 as a biomarker for Alzheimer's disease and other neurodegenerative disorders. *Neuromolecular Med.* **19**, 154–160 (2017).
68. Griffiths, M. R., Botto, M., Morgan, B. P., Neal, J. W. & Gasque, P. CD93 regulates central nervous system inflammation in two mouse models of autoimmune encephalomyelitis. *Immunology* **155**, 346–355 (2018).
69. Lugano, R. *et al.* CD93 maintains endothelial barrier function by limiting the phosphorylation and turnover of VE-cadherin. *FASEB J.* **37**, e22894 (2023).
70. Bryant, A. *et al.* Endothelial cells are heterogeneous in different brain regions and are dramatically altered in Alzheimer's disease. *J. Neurosci.* **43**, 4541–4557 (2023).
71. Cordon, J. *et al.* Identification of clinically relevant brain endothelial cell biomarkers in plasma. *Stroke* **54**, 2853–2863 (2023).
72. Piani, F. *et al.* Diagnostic and prognostic role of CD93 in cardiovascular disease: A systematic review. *Biomolecules* **13**, 910 (2023).
73. Zhang, B., Yang, N. & Gao, C. Is plasma C3 and C4 levels useful in young cerebral ischemic stroke patients? Associations with prognosis at 3 months. *J. Thromb. Thrombolysis* **39**, 209–214 (2015).
74. Olsson, S. *et al.* Genetic variation in complement component C3 shows association with ischaemic stroke: C3 and ischaemic stroke. *Eur. J. Neurol.* **18**, 1272–1274 (2011).
75. Mocco, J. *et al.* Complement component C3 mediates inflammatory injury following focal cerebral ischemia. *Circ. Res.* **99**, 209–217 (2006).
76. Yang, P. *et al.* Increased serum complement C3 levels are associated with adverse clinical outcomes after ischemic stroke. *Stroke* **52**, 868–877 (2021).
77. Tian, Q., Greig, E. E., Duggan, M. R., Walker, K. A. & Ferrucci, L. Plasma proteomic signatures of dual cognitive and mobility decline in older adults. *EBioMedicine* **118**, 105858 (2025).
78. Hallacli, E. *et al.* The Parkinson's disease protein alpha-synuclein is a modulator of processing bodies and mRNA stability. *Cell* **185**, 2035-2056.e33 (2022).
79. Imam, F. *et al.* The Global Neurodegeneration Proteomics Consortium: biomarker and drug target discovery for common neurodegenerative diseases and aging. *Nat. Med.* **31**, 2556–2566 (2025).
80. Sazer, S. & Dasso, M. The ran decathlon: multiple roles of Ran. *J. Cell Sci.* **113 (Pt 7)**, 1111–1118 (2000).
81. Kau, T. R., Way, J. C. & Silver, P. A. Nuclear transport and cancer: from mechanism to intervention. *Nat. Rev. Cancer* **4**, 106–117 (2004).
82. Yeo, E. J. *et al.* Tat-RAN attenuates brain ischemic injury in hippocampal HT-22 cells and ischemia

animal model. *Neurochem. Int.* **167**, 105538 (2023).
83. Kim, J. O. *et al.* Association of MicroRNA biogenesis genes polymorphisms with ischemic stroke susceptibility and post-stroke mortality. *J. Stroke* **20**, 110–121 (2018).
84. Sin, N. *et al.* The anti-angiogenic agent fumagillin covalently binds and inhibits the methionine aminopeptidase, MetAP-2. *Proc. Natl. Acad. Sci. U. S. A.* **94**, 6099–6103 (1997).
85. Clinkinbeard, T., Ghoshal, S., Craddock, S., Creed Pettigrew, L. & Guttmann, R. P. Calpain cleaves methionine aminopeptidase-2 in a rat model of ischemia/reperfusion. *Brain Res.* **1499**, 129–135 (2013).
86. Esa, R. *et al.* The role of methionine aminopeptidase 2 in lymphangiogenesis. *Int. J. Mol. Sci.* **21**, 5148 (2020).
87. Zhang, X. *et al.* Methionine aminopeptidase 2 (MetAP2) inhibitor BL6 attenuates inflammation in cultured microglia and in a mouse model of Alzheimer's disease. *Molecules* **30**, (2025).
88. Dafre, A. L., Taguchi, T., Dayn, Y., Currais, A. & Maher, P. Gender-dependent cognitive and metabolic benefits due to glyoxalase 1 (Glo1) Overexpression in age-accelerated SAMP8 mice. *Antioxidants (Basel)* **14**, 946 (2025).
89. Berends, E. *et al.* Glyoxalase 1 overexpression improves neurovascular coupling and limits development of mild cognitive impairment in a mouse model of type 1 diabetes. *J. Physiol.* **602**, 6209–6223 (2024).
90. Samanta, S. *et al.* Synaptic mitochondria glycation contributes to mitochondrial stress and cognitive dysfunction. *Brain* **148**, 262–275 (2025).
91. Butovsky, O. *et al.* Identification of a unique TGF-β-dependent molecular and functional signature in microglia. *Nat. Neurosci.* **17**, 131–143 (2014).
92. Bedolla, A. *et al.* Adult microglial TGFβ1 is required for microglia homeostasis via an autocrine mechanism to maintain cognitive function in mice. *Nat. Commun.* **15**, 5306 (2024).
93. Shea, J. M. & Villeda, S. A. Microglia aging in the hippocampus advances through intermediate states that drive inflammatory activation and cognitive decline. *eLife* (2024) doi:10.7554/elife.97671.1.
94. Su, C., Miao, J. & Guo, J. The relationship between TGF-β1 and cognitive function in the brain. *Brain Res. Bull.* **205**, 110820 (2023).
95. Pan, S. *et al.* TGF-β1 is associated with deficits in cognition and cerebral cortical thickness in first-episode schizophrenia. *J. Psychiatry Neurosci.* **47**, E86–E98 (2022).
96. Brionne, T. C., Tesseur, I., Masliah, E. & Wyss-Coray, T. Loss of TGF-beta 1 leads to increased neuronal cell death and microgliosis in mouse brain. *Neuron* **40**, 1133–1145 (2003).
97. Buchman, A. S. *et al.* Glycoproteome-wide discovery of cortical glycoproteins that may provide cognitive resilience in older adults. *Neurology* **102**, e209223 (2024).
98. Zammit, A. R. *et al.* Cortical proteins and individual differences in cognitive resilience in older adults. *Neurology* **98**, e1304–e1314 (2022).
99. Ramos-Miguel, A. *et al.* Proteomic identification of select protein variants of the SNARE interactome associated with cognitive reserve in a large community sample. *Acta Neuropathol.* **141**, 755–770 (2021).
100. Zammit, A. R. *et al.* The temporal onset of associations of cortical proteins with cognitive resilience vary during late life. *Neurobiol. Dis.* **211**, 106927 (2025).
101. Bhatt, A. M. & Setty, S. R. G. STX1A localizes to the lysosome and controls its exocytosis. *bioRxiv* 2025.03.29.646068 (2025) doi:10.1101/2025.03.29.646068.
102. Kelly, M. P. *et al.* Phosphodiesterase 11A in brain is enriched in ventral hippocampus and deletion causes psychiatric disease-related phenotypes. *Proc. Natl. Acad. Sci. U. S. A.* **107**, 8457–8462 (2010).
103. Hegde, S. *et al.* Phosphodiesterase 11A (PDE11A), enriched in ventral hippocampus neurons, is required for consolidation of social but not nonsocial memories in mice. *Neuropsychopharmacology* **41**, 2920–2931 (2016).
104. Pilarzyk, K. *et al.* Loss of function of phosphodiesterase 11A4 shows that recent and remote long-term memories can be uncoupled. *Curr. Biol.* **29**, 2307-2321.e5 (2019).
105. Pilarzyk, K. *et al.* Conserved age-related increases in hippocampal PDE11A4 cause unexpected proteinopathies and cognitive decline of social associative memories. *Aging Cell* **21**, e13687 (2022).
106. Mahmood, S. U. *et al.* First optimization of novel, potent, selective PDE11A4 inhibitors for age-related cognitive decline. *J. Med. Chem.* **66**, 14597–14608 (2023).
107. Fitzgerald, G. S., Chuchta, T. G. & McNay, E. C. Insulin-like growth factor-2 is a promising candidate for the treatment and prevention of Alzheimer's disease. *CNS Neurosci. Ther.* **29**, 1449–1469 (2023).
108. Pascual-Lucas, M. *et al.* Insulin-like growth factor 2 reverses memory and synaptic deficits in APP transgenic mice. *EMBO Mol. Med.* **6**, 1246–1262 (2014).
109. Yu, X.-W., Pandey, K., Katzman, A. C. & Alberini, C. M. A role for CIM6P/IGF2 receptor in memory consolidation and enhancement. *Elife* **9**, (2020).

110. Duggan, M. R. *et al.* OMG! A proteomic determinant of neurodegenerative resiliency. *Mol. Neurodegener.* 1–18 (2026).
111. Buckley, R. F., Gong, J. & Woodward, M. A call to action to address sex differences in Alzheimer disease clinical trials. *JAMA Neurol.* **80**, 769–770 (2023).

GNPC V1 Full Membership List and Affiliations

- Gamal Abdel-Azim, Johnson & Johnson, Spring House, USA
- Charles H Adler, Mayo Clinic Arizona, Scottsdale, Arizona, USA
- Muhammad Ali, Washington University School of Medicine, Department of Psychiatry, St. Louis, 63110, MO, USA.; NeuroGenomics and Informatics Center, Washington University School of Medicine, St. Louis, 63110, MO, USA.
- Federica Anastasi, Barcelonaβeta Brain Research Center (BBRC), Pasqual Maragall Foundation, Barcelona, Spain; Hospital del Mar Research Institute, Barcelona, Spain; Centre for Genomic Regulation (CRG), Barcelona Institute of Science and Technology (BIST), Barcelona, Spain
- Alireza Atri, Banner Sun Health Research Institute, Sun City, Arizona, USA
- Thomas G Beach, Banner Sun Health Research Institute, Sun City, Arizona, USA
- Graham Bearden, Alzheimer's Disease Data Initiative, Kirkland, WA
- David Bennett, Rush Alzheimer's Disease Center, Department of Neurological Sciences, Chicago, IL, USA
- James D. Berry, Sean M. Healey and AMG Center for ALS, Neurology
- Merce Boada, Ace Alzheimer Center Barcelona, Universitat Internacional de Catalunya, 08029 Barcelona, Spain; Biomedical Research Networking Centre in Neurodegenerative Diseases (CIBERNED), National Institute of Health Carlos III, 28029 Madrid, Spain
- Merle Bode, Hertie Institute for Clinical Brain Research, Neurodegenerative Diseases, Tübingen; German Center of Neurodegenerative Diseases , Department of Neurodegenerative Diseases, Tübingen
- Bradley Boeve, Mayo Clinic, Neurology Department, Rochester, MN
- Niranjan Bose, Gates Ventures, Seattle, WA
- Veronica Bot, Stanford University, The Phil and Penny Knight Initiative for Brain Resilience, Stanford, CA, USA; Stanford University, Wu Tsai Neurosciences Institute, Stanford, CA, USA; Stanford University, Graduate Program in Biomedical Engineering, Stanford, CA, USA
- Hillary Bounds, Gates Ventures, Seattle, WA
- Adam L. Boxer, University of California, San Francisco, Neurology Department, San Francisco, CA
- Martin Bringmann, Johnson & Johnson, Spring House, USA
- Jeffrey M. Burns, University of Kansas Alzheimer's Disease Research Center, Kansas City, Kansas, USA; University of Kansas, Neurology, Kansas City, Kansas, USA
- Alfredo Cabrera-Socorro, Johnson & Johnson, NS TA, Beerse, Belgium
- Amanda Fernandez Cano, Ace Alzheimer Center Barcelona, Universitat Internacional de Catalunya, 08029 Barcelona, Spain; Biomedical Research Networking Centre in Neurodegenerative Diseases (CIBERNED), National Institute of Health Carlos III, 28029 Madrid, Spain
- Kaitlin B. Casaletto, University of California, San Francisco, Neurology Department, San Francisco, CA
- Richard J Caselli, Mayo Clinic Arizona, Scottsdale, Arizona, USA
- Yike Chen, Washington University School of Medicine, Department of Psychiatry, St. Louis, 63110, MO, USA.; NeuroGenomics and Informatics Center, Washington University School of Medicine, St. Louis, 63110, MO, USA.
- Matthew H.S. Clement, Alzheimer's Disease Data Initiative, Kirkland, WA
- Carlos Cruchaga, Washington University School of Medicine, Department of Psychiatry, St. Louis, 63110, MO, USA.; NeuroGenomics and Informatics Center, Washington University School of Medicine, St. Louis, 63110, MO, USA.; Washington University School of Medicine, Department of Neurology, St. Louis, 63110, MO, USA.
- Jeff Dage, Indiana Alzheimer's Disease Research Center, Indianapolis, IN; Indiana University School of Medicine, Department of Neurology, Indianapolis, IN
- Eric B. Dammer, Emory University School of Medicine, Atlanta, GA, USA; Emory University School of Medicine, Department of Biochemistry, Atlanta, GA, USA

- Sterre de Boer, Alzheimer Center Amsterdam, Neurology, Amsterdam UMC, Amsterdam, the Netherlands; Amsterdam Neuroscience, Amsterdam, the Netherlands
- Niels De Meirleir, Johnson & Johnson, NS TA, Beerse, Belgium
- Marta del Campo Milan, Barcelonaβeta Brain Research Center (BBRC), Pasqual Maragall Foundation, Barcelona, Spain; Hospital del Mar Research Institute, Barcelona, Spain
- Daisy Ding, Stanford University, The Phil and Penny Knight Initiative for Brain Resilience, Stanford, CA, USA; Stanford University, Wu Tsai Neurosciences Institute, Stanford, CA, USA; Stanford University, Graduate Program in Biomedical Engineering, Stanford, CA, USA
- Duc Duong, Emory University School of Medicine, Atlanta, GA, USA; Emory University School of Medicine, Department of Biochemistry, Atlanta, GA, USA
- Amelia Farinas, Stanford University, Graduate Program in Neuroscience, Stanford, CA, USA; Stanford University, The Phil and Penny Knight Initiative for Brain Resilience, Stanford, CA, USA; Stanford University, Wu Tsai Neurosciences Institute, Stanford, CA, USA
- Maria Victoria Fernandez, Ace Alzheimer Center Barcelona, Universitat Internacional de Catalunya, 08029 Barcelona, Spain
- Luigi Ferrucci, NIH/NIA, Translational Gerontology Branch, National Institute on Aging
- Caitlin A. Finney, Neurodegeneration and Precision Medicine Research Group, Westmead Institute for Medical Research, New South Wales, Australia; University of Sydney School of Medical Sciences, Faculty of Medicine and Health, New South Wales, Australia
- Lawrence Fourgeaud, Johnson & Johnson, NS TA, La Jolla, USA
- Mark Frasier, Michael J. Fox Foundation, New York, NY, USA
- Raquel Puerta Fuentes, Ace Alzheimer Center Barcelona, Universitat Internacional de Catalunya, 08029 Barcelona, Spain; PhD Program in Biotecnology, Faculty of Pharmacy and Food Sciences, University of Barcelona, 08028 Barcelona, Spain
- Jordan Fuller, Gates Ventures, Seattle, WA
- Su Gao, Indiana Alzheimer's Disease Research Center, Indianapolis, IN; Indiana University School of Medicine, Department of Biostatistics & Health Data Science, Indianapolis, IN
- John Gibbons, Rush Alzheimer's Disease Center, Department of Neurological Sciences, Chicago, IL, USA
- Pablo Garcia Gonzalez, Ace Alzheimer Center Barcelona, Universitat Internacional de Catalunya, 08029 Barcelona, Spain; Biomedical Research Networking Centre in Neurodegenerative Diseases (CIBERNED), National Institute of Health Carlos III, 28029 Madrid, Spain
- Gyujin Heo, Washington University School of Medicine, Department of Psychiatry, St. Louis, 63110, MO, USA.; NeuroGenomics and Informatics Center, Washington University School of Medicine, St. Louis, 63110, MO, USA.
- Hilary Heuer, University of California, San Francisco, Neurology Department, San Francisco,
- CA
- Timothy J. Hohman, Vanderbilt Memory & Alzheimer's Disease, Department Neurology, Vanderbilt University Medical Center, Nashville, TN ; Vanderbilt Genetics Institute, Vanderbilt Medical Center, Nashville, TN, USA
- Liping Hou, Johnson & Johnson, Spring House, USA
- Yen-Ning Huang, Indiana Alzheimer's Disease Research Center, Indianapolis, IN; Indiana University School of Medicine, Department of Radiology & Imaging Sciences, Indianapolis, IN
- Alina Isakova, Stanford University, The Phil and Penny Knight Initiative for Brain Resilience, Stanford, CA, USA
- Clifford R. Jack, Jr, Mayo Clinic, Radiology
- Erik C.B. Johnson, Emory University School of Medicine, Atlanta, GA, USA; Emory University School of Medicine, Department of Neurology, Atlanta, GA, USA
- Mika Kivimaki, University College London, UCL Brain Sciences, London, UK; University of Helsinki, Clinicum, Helsinki, Finland
- Emily Kogan, Johnson & Johnson, JRD DSDH, Cambridge, USA

- Roxanna Korologou-Linden, Ageing & Epidemiology (AGE) Research Unit, School of Public Health, Imperial College London, UK
- Jessica B Langbaum, Banner Alzheimer's Institute, Phoenix, Arizona, USA
- Argentina Lario-Lago, University of California, San Francisco, Neurology Department, San Francisco, CA
- Agustin Ruiz Laza, Ace Alzheimer Center Barcelona, Universitat Internacional de Catalunya, 08029 Barcelona, Spain; Biomedical Research Networking Centre in Neurodegenerative Diseases (CIBERNED), National Institute of Health Carlos III, 28029 Madrid, Spain; Glenn Biggs Institute for Alzheimer's & Neurodegenerative Diseases and Department of Microbiology, Immunology and Molecular Genetics, Long School of Medicine, University of Texas Health Science Center, San Antonio, TX 77204, USA
- Allan I. Levey, Emory University School of Medicine, Atlanta, GA, USA; Emory University School of Medicine, Department of Neurology, Atlanta, GA, USA
- Shuwei Li, Johnson & Johnson, Spring House, USA
- Inga Liepelt-Scarfone, Hertie Institute for Clinical Brain Research, Neurodegenerative Diseases, Tübingen; German Center of Neurodegenerative Diseases , Department of Neurodegenerative Diseases, Tübingen; IB Hochschule für Gesundheit und Soziales , Standort Stuttgart
- Shiwei Liu, Indiana Alzheimer's Disease Research Center, Indianapolis, IN; Indiana University School of Medicine, Department of Radiology & Imaging Sciences, Indianapolis, IN
- Menghan Liu, Washington University School of Medicine, Department of Psychiatry, St. Louis, 63110, MO, USA.; NeuroGenomics and Informatics Center, Washington University School of Medicine, St. Louis, 63110, MO, USA.
- Simon Lovestone, Johnson & Johnson, London, UK
- Lina Lu, Clinical Memory Research Unit, Department of Clinical Sciences Malmö, Lund University, Lund, Sweden
- Marta Marquie, Ace Alzheimer Center Barcelona, Universitat Internacional de Catalunya, 08029 Barcelona, Spain; Biomedical Research Networking Centre in Neurodegenerative Diseases (CIBERNED), National Institute of Health Carlos III, 28029 Madrid, Spain
- Caitlin P. McHugh, Alzheimer's Disease Data Initiative, Kirkland, WA
- Martine Meyer, Johnson & Johnson, NS TA
- Lefkos T. Middelton, Ageing & Epidemiology (AGE) Research Unit, School of Public Health, Imperial College London, UK
- Silke Miller, Johnson & Johnson, NS TA, La Jolla, USA
- Elizabeth Mlynarski, Johnson & Johnson, JRD DSDH, Spring House, USA
- Diederik Moechars, Johnson & Johnson, NS TA, Beerse, Belgium
- Patricia Moran-Losada, Stanford University, The Phil and Penny Knight Initiative for Brain Resilience, Stanford, CA, USA; Stanford University, Wu Tsai Neurosciences Institute, Stanford, CA, USA; Stanford University School of Medicine, Department of Neurology and Neurological Sciences, Stanford, CA, USA
- Kwangsik Nho, Indiana Alzheimer's Disease Research Center, Indianapolis, IN; Indiana University School of Medicine, Department of Radiology & Imaging Sciences, Indianapolis, IN
- Hamilton Oh, Stanford University, The Phil and Penny Knight Initiative for Brain Resilience, Stanford, CA, USA; Stanford University, Wu Tsai Neurosciences Institute, Stanford, CA, USA; Mount Sinai, Icahn School of Medicine at Mount Sinai, New York NY, USA
- Paige Opsahl, Gates Ventures, Seattle, WA
- Tamina Park, Indiana Alzheimer's Disease Research Center, Indianapolis, IN; Indiana University School of Medicine, Department of Radiology & Imaging Sciences, Indianapolis, IN
- Ronald C. Petersen, Mayo Clinic, Neurology, Rochester, MN
- Mukta Phatak, Alzheimer's Disease Data Initiative, Kirkland, WA
- Joni Lindbohm, MD, PhD, University College London, UCL Brain Sciences, London, UK; University of Helsinki, Clinicum, Helsinki, Finland
- Joseph Pick, Johnson & Johnson, Spring House, USA

- Yolande AL Pijnenburg, Alzheimer Center Amsterdam, Neurology Department, Amsterdam, the Netherlands; Amsterdam Neuroscience, Amsterdam, the Netherlands
- Michael Price, Michael J. Fox Foundation, New York, NY, USA
- Eric M Reiman, Banner Alzheimer's Institute, Phoenix, Arizona, USA
- Shannon Risacher, Indiana Alzheimer's Disease Research Center, Indianapolis, IN; Indiana University School of Medicine, Department of Radiology & Imaging Sciences, Indianapolis, IN
- Oliver Robinson, Ageing & Epidemiology (AGE) Research Unit, School of Public Health, Imperial College London, UK; Department of Epidemiology and Biostatistics, School of Public Health, Imperial College London, UK
- Julio C. Rojas, University of California, San Francisco, Neurology Department, San Francisco, CA
- Howard J. Rosen, University of California, San Francisco, Neurology Department, San Francisco, CA
- Jeffrey D. Rothstein, Johns Hopkins University, Robert Packard Center for ALS Research, Baltimore, MD, USA
- Tamsin Sargood, Johnson & Johnson, Global Development, UK
- Andrew J. Saykin, Indiana Alzheimer's Disease Research Center, Indianapolis, IN; Indiana University School of Medicine, Department of Neurology, Indianapolis, IN
- Claudia Schulte, Hertie Institute for Clinical Brain Research, Neurodegenerative Diseases, Tübingen; German Center of Neurodegenerative Diseases , Department of Neurodegenerative Diseases, Tübingen
- Weiwei Schultz, Johnson & Johnson, JRD DSDH, Titusville, USA
- Geidy E Serrano, Banner Sun Health Research Institute, Sun City, Arizona, USA
- Nicholas T. Seyfried, Emory University School of Medicine, Atlanta, GA, USA; Emory University School of Medicine, Department of Neurology, Atlanta, GA, USA; Emory University School of Medicine, Department of Biochemistry, Atlanta, GA, USA
- Todd Sherer, Michael J. Fox Foundation, New York, NY, USA
- Artur Shvetcov, Neurodegeneration and Precision Medicine Research Group, Westmead Institute for Medical Research, New South Wales, Australia; University of Sydney School of Medical Sciences, Faculty of Medicine and Health, New South Wales, Australia
- Chad Slawson, University of Kansas Alzheimer's Disease Research Center, Kansas City, Kansas, USA; University of Kansas, Biochemistry and Molecular Biology, Kansas City, Kansas, USA
- Emily Smith, Indiana Alzheimer's Disease Research Center, Indianapolis, IN; Indiana University School of Medicine, Department of Radiology & Imaging Sciences, Indianapolis, IN
- Adam M. Staffaroni, University of California, San Francisco, Neurology Department, San Francisco, CA
- Marc Suárez-Calvet, Barcelonaβeta Brain Research Center (BBRC), Pasqual Maragall Foundation, Barcelona, Spain; Hospital del Mar Research Institute, Barcelona, Spain; Hospital del Mar, Neurology Department, Barcelona, Spain
- Russell H. Swerdlow, University of Kansas Alzheimer's Disease Research Center, Kansas City, Kansas, USA; University of Kansas, Neurology, Kansas City, Kansas, USA
- Charlotte Teunissen, Neurochemistry Laboratory, Neurology Department, Amsterdam, the Netherlands; Amsterdam Neuroscience, Amsterdam, the Netherlands
- Terri G. Thompson, OnPoint Scientific, Inc, San Diego, CA, USA
- Qu Tian, NIH/NIA
- Betty M Tijms, Alzheimer Center Amsterdam, Neurology Department, Amsterdam, the Netherlands; Amsterdam Neuroscience, Amsterdam, the Netherlands
- Maarten Timmers, Johnson & Johnson, Beerse, Belgium
- Jigyasha Timsina, Washington University School of Medicine, Department of Psychiatry, St. Louis, 63110, MO, USA.; NeuroGenomics and Informatics Center, Washington University School of Medicine, St. Louis, 63110, MO, USA.
- Abolfazl Doostparast torshizi, Johnson & Johnson, Spring House, USA

- Sergi Valero, Ace Alzheimer Center Barcelona, Universitat Internacional de Catalunya, 08029 Barcelona, Spain; Biomedical Research Networking Centre in Neurodegenerative Diseases (CIBERNED), National Institute of Health Carlos III, 28029 Madrid, Spain
- Wiesje M van der Flier, Alzheimer Center Amsterdam, Neurology Department, Amsterdam, the Netherlands; Amsterdam Neuroscience, Amsterdam, the Netherlands; Epidemiology and Data Science, Amsterdam UMC
- Fernando Vieira, ALS Therapy Development Institute, Cambridge, MA, United States
- Natalia Vilor-Tejedor, Barcelonaβeta Brain Research Center (BBRC), Pasqual Maragall Foundation, Barcelona, Spain; Radboud University Medical Center, Department of Human Genetics, Nijmegen, Netherlands; Centre for Genomic Regulation (CRG), Barcelona Institute of Science and Technology (BIST), Barcelona, Spain
- Pieter Jelle Visser, Alzheimer Center Amsterdam, Neurology Department, Amsterdam, the Netherlands; Amsterdam Neuroscience, Amsterdam, the Netherlands; Alzheimer center Limburg, School for Mental Health and Neuroscience, Maastricht University
- Keenan A Walker, NIH/NIA, Laboratory of Behavioral Neuroscience, National Institute on Aging
- Julia D. Webb, University of California, San Francisco, Neurology Department, San Francisco, CA
- Laura M. Winchester, Oxford University, Department of Psychiatry, Oxford, UK
- Bryan K Woodruff, Mayo Clinic Arizona, Scottsdale, Arizona, USA
- Tony Wyss-Coray, Stanford University, The Phil and Penny Knight Initiative for Brain Resilience, Stanford, CA, USA; Stanford University, Wu Tsai Neurosciences Institute, Stanford, CA, USA; Stanford University School of Medicine, Department of Neurology and Neurological Sciences, Stanford, CA, USA
- Ying Xu, Washington University School of Medicine, Department of Psychiatry, St. Louis, 63110, MO, USA.; NeuroGenomics and Informatics Center, Washington University School of Medicine, St. Louis, 63110, MO, USA.
- Chengran Yang, Washington University School of Medicine, Department of Psychiatry, St. Louis, 63110, MO, USA.; NeuroGenomics and Informatics Center, Washington University School of Medicine, St. Louis, 63110, MO, USA.
- Mariet A. Younkin, Mayo Clinic, Neurology, Rochester, MN
