# Supplementary material for: A deep joint-learning proteomics model for diagnosis of six conditions associated with dementia
Source: Nat Med. 2026 Mar 31;32(5):1852–64. doi: 10.1038/s41591-026-04303-y (PMC13190262; doi:10.1038/s41591-026-04303-y)
Supplement: Supplementary file 2 — Reporting Summary [file 41591_2026_4303_MOESM2_ESM.pdf]

nature portfolio

Corresponding author(s): Jacob W. Vogel, Lijun An

Last updated by author(s): Feb 9, 2026

Reporting Summary

Nature Portfolio wishes to improve the reproducibility of the work that we publish. This form provides structure for consistency and transparency in reporting. For further information on Nature Portfolio policies, see our Editorial Policies and the Editorial Policy Checklist.

Statistics

For all statistical analyses, confirm that the following items are present in the figure legend, table legend, main text, or Methods section.

n/a	Confirmed	
☐	☒	The exact sample size (*n*) for each experimental group/condition, given as a discrete number and unit of measurement
☐	☒	A statement on whether measurements were taken from distinct samples or whether the same sample was measured repeatedly
☐	☒	The statistical test(s) used AND whether they are one- or two-sided<br>*Only common tests should be described solely by name; describe more complex techniques in the Methods section.*
☐	☒	A description of all covariates tested
☐	☒	A description of any assumptions or corrections, such as tests of normality and adjustment for multiple comparisons
☐	☒	A full description of the statistical parameters including central tendency (e.g. means) or other basic estimates (e.g. regression coefficient) AND variation (e.g. standard deviation) or associated estimates of uncertainty (e.g. confidence intervals)
☒	☐	For null hypothesis testing, the test statistic (e.g. *F*, *t*, *r*) with confidence intervals, effect sizes, degrees of freedom and *P* value noted<br>*Give P values as exact values whenever suitable.*
☒	☐	For Bayesian analysis, information on the choice of priors and Markov chain Monte Carlo settings
☒	☐	For hierarchical and complex designs, identification of the appropriate level for tests and full reporting of outcomes
☐	☒	Estimates of effect sizes (e.g. Cohen's *d*, Pearson's *r*), indicating how they were calculated

Our web collection on statistics for biologists contains articles on many of the points above.

Software and code

Policy information about availability of computer code

Data collection	The data is directly downloaded from GNPC database. No data collection software/code applicable.
Data analysis	All code are publicly available at: https://github.com/DeMONLab-BioFINDER/An_ProtAIDe-Dx;<br>We mainly used Python (v3.9.21), torch (v2.5.0), numpy (v2.0.2), scikit-learn (v1.6.1), scipy (v1.13.1), XGBoost (v2.1.4), and tabpfn (v2.1.0) for our analysis. For detailed information, please check our configuration file at: https://github.com/DeMONLab-BioFINDER/An_ProtAIDe-Dx/blob/main/replication/config/DeMONLab_ProtAIDe_env.yaml

For manuscripts utilizing custom algorithms or software that are central to the research but not yet described in published literature, software must be made available to editors and reviewers. We strongly encourage code deposition in a community repository (e.g. GitHub). See the Nature Portfolio guidelines for submitting code & software for further information.

Data

Policy information about availability of data

All manuscripts must include a data availability statement. This statement should provide the following information, where applicable:

- Accession codes, unique identifiers, or web links for publicly available datasets
- A description of any restrictions on data availability
- For clinical datasets or third party data, please ensure that the statement adheres to our policy

GNPC (https://www.neuroproteome.org/) is open-access. Pseudonymized BioFINDER-2 data will be shared by request from a qualified academic investigator for the

sole purpose of replicating procedures and results presented in the article and as long as data transfer is in agreement with EU legislation on the general data protection regulation and decisions by the Swedish Ethical Review Authority and Region Skåne, which should be regulated in a material transfer agreement.

Research involving human participants, their data, or biological material

Policy information about studies with human participants or human data. See also policy information about sex, gender (identity/presentation), and sexual orientation and race, ethnicity and racism.

Reporting on sex and gender	Sex was self-reported. All statistical analysis included sex as covariate.
Reporting on race, ethnicity, or other socially relevant groupings	Race and ethnicity were not included as confounding factors in this manuscript.
Population characteristics	Detailed information is given in Tables S1, S2 and S12.
Recruitment	The participants were either health aging people, patients with mild cognitive impairment, or with five of the most common incapacitating age-related neurological conditions in old age – AD, PD, neurovascular disease (represented here as stroke), ALS and frontotemporal dementia (FTD).
Ethics oversight	Ethical approvals were given by the IRBs of all participating cohorts in GNPC. All studies were approved by the Institutional Review Board (IRB) of Lund University and written informed consent or assent was obtained from all participants or their legally authorized representative.

Note that full information on the approval of the study protocol must also be provided in the manuscript.

Field-specific reporting

Please select the one below that is the best fit for your research. If you are not sure, read the appropriate sections before making your selection.

☒ Life sciences ☐ Behavioural & social sciences ☐ Ecological, evolutionary & environmental sciences

For a reference copy of the document with all sections, see nature.com/documents/nr-reporting-summary-flat.pdf

Life sciences study design

All studies must disclose on these points even when the disclosure is negative.

Sample size	We selected all GNPC (N=17,187) and BioFIDNER-2 (N=1,786) participants with SomaLogic 7k plasma proteomics data.
Data exclusions	We excluded subjects without SomaLogic 7k plasma proteomics data.
Replication	We replicated our model trained on GNPC on external clinical cohort BioFIDNER-2.
Randomization	We performed 10-fold cross validation with random seeds for randomization.
Blinding	Proteomic measurements were performed blinded to any demographics or clinical characteristics.

Reporting for specific materials, systems and methods

We require information from authors about some types of materials, experimental systems and methods used in many studies. Here, indicate whether each material, system or method listed is relevant to your study. If you are not sure if a list item applies to your research, read the appropriate section before selecting a response.

Materials & experimental systems

n/a	Involved in the study	
☐	☒	Antibodies
☒	☐	Eukaryotic cell lines
☒	☐	Palaeontology and archaeology
☒	☐	Animals and other organisms
☐	☒	Clinical data
☒	☐	Dual use research of concern
☒	☐	Plants

Methods

n/a	Involved in the study	
☒	☐	ChIP-seq
☒	☐	Flow cytometry
☐	☒	MRI-based neuroimaging

Antibodies

Antibodies used	Details about antibody-based proteomic technology is provided by SomaLogic with all details found here: https://somalogic.com/wp-content/uploads/2022/04/SL00000712_Rev-1_2022-03_SomaScan-Infographic-1.pdf
Validation	Extensive validation has been conducted by SomaLogic with all details of the SomaLogic 7k Assay available here: https://somalogic.com/poster/somascan-platform-confirmation-and-performance-validation/

Clinical data

Policy information about clinical studies

All manuscripts should comply with the ICMJE guidelines for publication of clinical research and a completed CONSORT checklist must be included with all submissions.

Clinical trial registration	BioFINDER-2: NCT03174938
Study protocol	Please see www.biofinder.se for BioFINDER studies
Data collection	BioFINDER-2 participants include a mix of population-based and memory clinic-based studies in Lund and Malmö, in Sweden and all imaging data was acquired at Skane University Hospital between April 2017 and December 2022.
Outcomes	The primary outcomes was that ProtAIDe-Dx drived proteomics signatures provided comparable performance to an automated diagnosis combining multiple neuroimaging and clinical biomarkers currently used in memory clinics.

Plants

Seed stocks	N/A. We could not unselect this tab.
Novel plant genotypes	N/A. We could not unselect this tab.
Authentication	N/A. We could not unselect this tab.

Magnetic resonance imaging

Experimental design

Design type	Structural MRI imaging.
Design specifications	Whole brain structural MRI scan.
Behavioral performance measures	No behavior performances measures during structural imaging.

Acquisition

Imaging type(s)	structural
Field strength	3
Sequence & imaging parameters	T1-weighted images were acquired on a Siemens Prisma scanner (Siemens Medical Solutions) with a 64-channel head coil using an MPRAGE sequence (in-plane resolution=1×1 mm2, slice thickness=1 mm, repetition time=1900 ms, echo time=2.54ms, flip-angle=9°).
Area of acquisition	Whole brain scan.

Diffusion MRI ☐ Used ☒ Not used

Preprocessing

Preprocessing software	FreeSurfer version 6.0

Normalization: Volume of white matter lesion (SAMSEG) was normalied by intracrainal volume.

Normalization template: Data extracted from single subject space.

Noise and artifact removal: N.A.

Volume censoring: N.A.

Statistical modeling & inference

Model type and settings: Predictive

Effect(s) tested: Prediction of diagnosis group of patients using structural MRI.

Specify type of analysis: ☐ Whole brain ☒ ROI-based ☐ Both

Anatomical location(s): AD-signature meta-ROI, which is the mean cortical thickness in the following individual ROIs: entorhinal, inferior temporal, middle temporal, and fusiform.

Statistic type for inference (See Eklund et al. 2016): N.A.

Correction: N.A.

Models & analysis

n/a	Involved in the study
☒	☐ Functional and/or effective connectivity
☒	☐ Graph analysis
☐	☒ Multivariate modeling or predictive analysis

Multivariate modeling and predictive analysis: We used SVM as machine learning to predict diagnosis group of patients.
